# Genomic insights into the secondary aquatic transition of penguins

**DOI:** 10.1038/s41467-022-31508-9

**Published:** 2022-07-19

**Authors:** Theresa L. Cole, Chengran Zhou, Miaoquan Fang, Hailin Pan, Daniel T. Ksepka, Steven R. Fiddaman, Christopher A. Emerling, Daniel B. Thomas, Xupeng Bi, Qi Fang, Martin R. Ellegaard, Shaohong Feng, Adrian L. Smith, Tracy A. Heath, Alan J. D. Tennyson, Pablo García Borboroglu, Jamie R. Wood, Peter W. Hadden, Stefanie Grosser, Charles-André Bost, Yves Cherel, Thomas Mattern, Tom Hart, Mikkel-Holger S. Sinding, Lara D. Shepherd, Richard A. Phillips, Petra Quillfeldt, Juan F. Masello, Juan L. Bouzat, Peter G. Ryan, David R. Thompson, Ursula Ellenberg, Peter Dann, Gary Miller, P. Dee Boersma, Ruoping Zhao, M. Thomas P. Gilbert, Huanming Yang, De-Xing Zhang, Guojie Zhang

**Affiliations:** 1 Villum Centre for Biodiversity Genomics, Section for Ecology and Evolution, Department of Biology, University of Copenhagen, Ole Maaløes Vej 5, 2200 Copenhagen, Denmark; 2 BGI-Shenzhen, Shenzhen, 518083 China; 3 Bruce Museum, Greenwich, CT 06830 USA; 4 Department of Zoology, Peter Medawar Building for Pathogen Research, University of Oxford, Oxford, OX1 3SZ UK; 5 Biology Department, Reedley College, Reedley, CA 93654 USA; 6 School of Natural Sciences, Massey University, Auckland, 0632 New Zealand; 7 Evolutionary & Organismal Biology Research Center, Zhejiang University School of Medicine, Hangzhou, 310058 China; 8 Center for Evolutionary Hologenomicss, The GLOBE Institute, Faculty of Health and Medical Sciences, University of Copenhagen, Øster Farimagsgade 5A, 1353 Copenhagen, Denmark; 9 Department of Natural History, NTNU University Museum, Norwegian University of Science and Technology, Erling Skakkes gate 47A, 7012 Trondheim, Norway; 10 Department of Ecology, Evolution, and Organismal Biology, Iowa State University, 2200 Osborn Dr., Ames, IA 50011 USA; 11 Museum of New Zealand Te Papa Tongarewa, PO Box 467, Wellington, 6140 New Zealand; 12 Center for Ecosystem Sentinels, Department of Biology, University of Washington, Seattle, WA 98195 USA; 13 Global Penguin Society, Puerto Madryn, 9120 Argentina; 14 CESIMAR CCT Cenpat-CONICET, Puerto Madryn, 9120 Chubut Argentina; 15 School of Biological Sciences, Faculty of Science, Engineering and Technology, University of Adelaide, North Terrace Campus, Adelaide, SA 5005 Australia; 16 Department of Ophthalmology, New Zealand National Eye Centre, Faculty of Medical and Health Sciences, University of Auckland, Private Bag 92019, Auckland, 1142 New Zealand; 17 Department of Zoology, University of Otago, Dunedin, 9054 New Zealand; 18 Centre d’Etudes Biologiques de Chizé (CEBC), UMR 7372 du CNRS-La Rochelle Université, 79360 Villiers-en-Bois, France; 19 British Antarctic Survey, Natural Environment Research Council, High Cross, Cambridge, CB3 0ET UK; 20 Department of Animal Ecology and Systematics, Justus-Liebig-Universität Giessen, Heinrich-Buff-Ring 26, 35392 Giessen, Germany; 21 Department of Biological Sciences, Bowling Green State University, Bowling Green, OH 43403 USA; 22 FitzPatrick Institute of African Ornithology, University of Cape Town, Rondebosch, 7701 South Africa; 23 National Institute of Water and Atmospheric Research Ltd., Private Bag 14901, Kilbirnie, Wellington 6241 New Zealand; 24 Department of Ecology, Environment and Evolution, La Trobe University, Melbourne, VIC 3086 Australia; 25 Department of Marine Science, University of Otago, Dunedin, 9016 New Zealand; 26 Research Department, Phillip Island Nature Parks, PO Box 97, Cowes, Phillip Island, Cowes, VIC 3922 Australia; 27 Division of Pathology and Laboratory Medicine, University of Western Australia, Crawley, WA 6009 Australia; 28 Institute for Marine and Antarctic Studies, University of Tasmania, Hobart, TAS 7001 Australia; 29 State Key Laboratory of Genetic Resources and Evolution, Kunming Institute of Zoology, Chinese Academy of Sciences, Kunming, 650223 China; 30 James D. Watson Institute of Genome Sciences, Hangzhou, 310029 China; 31 Guangdong Provincial Academician Workstation of BGI Synthetic Genomics, BGI-Shenzhen, Shenzhen, 518120 China; 32 Center for Computational and Evolutionary Biology & State Key Laboratory of Integrated Management of Pest Insects and Rodents, Institute of Zoology, Chinese Academy of Sciences, 1 Beichen West Road, Beijing, 100101 China; 33 College of Life Sciences, University of Chinese Academy of Sciences, 19 Yuquan Road, Beijing, 100049 China; 34 Innovation Center of Yangtze River Delta, Zhejiang University, 314102 Jiashan, China

**Keywords:** Biodiversity, Evolutionary genetics, Zoology

## Abstract

Penguins lost the ability to fly more than 60 million years ago, subsequently evolving a hyper-specialized marine body plan. Within the framework of a genome-scale, fossil-inclusive phylogeny, we identify key geological events that shaped penguin diversification and genomic signatures consistent with widespread refugia/recolonization during major climate oscillations. We further identify a suite of genes potentially underpinning adaptations related to thermoregulation, oxygenation, diving, vision, diet, immunity and body size, which might have facilitated their remarkable secondary transition to an aquatic ecology. Our analyses indicate that penguins and their sister group (Procellariiformes) have the lowest evolutionary rates yet detected in birds. Together, these findings help improve our understanding of how penguins have transitioned to the marine environment, successfully colonizing some of the most extreme environments on Earth.

## Abstract (web summary)

This study examines the tempo and drivers of penguin diversification by combining genomes from all extant and recently extinct penguin lineages, stratigraphic data from fossil penguins and morphological and biogeographic data from all extant and extinct species. Together, these datasets provide new insights into the genetic basis and evolution of adaptations in penguins.

## Introduction

Penguins are one of the most iconic groups of birds, serving as both a textbook example of the evolution of secondarily aquatic ecology and as sentinels for the impacts of global change on ecosystem health^1^. Although often associated with Antarctica in the popular imagination, penguins originated more than 60 million years ago (Mya), evolving wing-propelled diving and losing the capacity for aerial flight long before the formation of polar ice sheets^2^. Over time, penguins evolved the suite of morphological, physiological, and behavioral features that make them arguably the most uniquely specialized of all extant birds. These adaptations have allowed penguins to colonize some of the most extreme environments on Earth.

Previous phylogenetic studies have yielded insights into penguin evolution, yet have been limited by sampling issues (e.g., number of lineages incorporated and quality of molecular markers^3–7^). Genomic studies have shed light on the diversification of extant penguins^7–9^ but have not integrated extinct species. Because nearly three-quarters of known penguin species are represented only by fossils (e.g., ^2,3^), sampling extinct species is crucial for improving phylogenetic resolution and dating accuracy, reconstructing biogeographic events, and understanding the environmental context in which key adaptations arose. While several studies have included fossil penguins, these utilized only mitochondrial genomes and/or small numbers of nuclear genes (e.g., ^3–6^), limiting their ability to disentangle confounding processes, such as historical and ongoing introgression and incomplete lineage sorting.

**Fig. 1:**
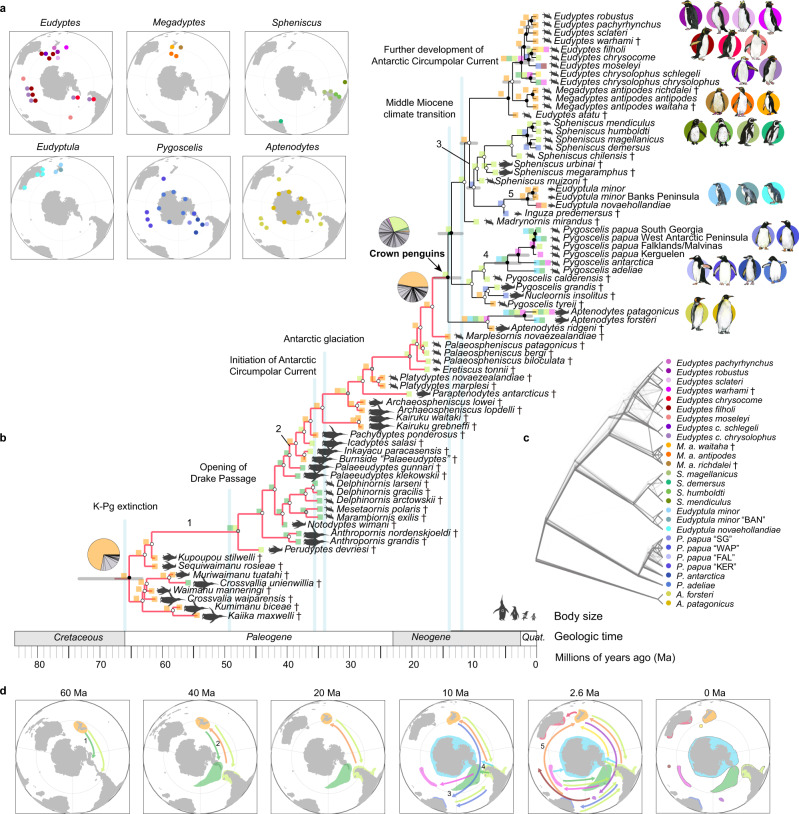
Phylogeny and biogeography of penguins. **a** Breeding range of extant/recently-extinct penguins. Colors of circles correspond to species identities shown in subplot **b**, **c**. Note, *Eudyptula novaehollandiae* and *Megadyptes antipodes antipodes* colonized New Zealand <800 years ago, so those expanded ranges are not shown. **b** Total-evidence maximum clade credibility tree incorporating ancestral range estimation from BioGeoBEARS under the best-fitting model (DEC+J+X). † indicates extinct taxa. Silhouettes in cladogram indicate approximate body size. The gray rectangles at the nodes among extant penguins represent 95% confidence intervals of the corresponding estimated divergence times. Circles at the nodes are colored to indicate posterior probability: black (>0.95), gray (0.75–0.95), white (<0.75). The single most probable ancestral range is indicated at each node using squares (colors represent the ranges in **d**) with the exception of three key nodes (pie charts, gray represents multiple ranges). Nodes are marked with a number corresponding to potential dispersal events. Major geological events are indicated. **c** Densitree of 500 random RAxML gene trees, summarizing gene discordance. **d** Paleomaps showing major inferred dispersal vectors for penguins across the Cenozoic. Arrows show one possible biogeographic scenario interpreted from the ancestral area reconstructions. Numbers correspond to numbered nodes in **b**. Source data is provided as a Source Data file.

Here, we take a comprehensive approach to inferring the tempo and drivers of penguin diversification by combining genomes from all extant and recently-extinct penguin lineages (27 taxa) (Table 1), stratigraphic data from fossil penguins (47 taxa), and morphological and biogeographic data from all species (extant and extinct) (Fig. 1 and Supplementary Fig. 1; Supplementary Data 1) into a single framework for Bayesian phylogenetic analysis. This combined approach, using the fossilized birth-death process with sampled ancestors^4^ (see supplementary methods) offers a more complete understanding of speciation and biogeographic events over the entire history of penguin evolution. It extends our insights beyond the ~15–20 million year (Ma) history of crown penguins to include the ~50 Ma interval during which only stem penguins existed. Within this phylogenetic framework, we highlight key genes involved in marine adaptations, compare evolutionary rates in penguins to those of other birds, and reconstruct the demographic histories of individual species. Together, these extensive datasets provide new insights into the evolution of extreme ecological preferences and the genetic basis for the adaptations that enabled penguins to occupy these niches.

**Table 1:** Penguin names.

Taxa	Common name
*Aptenodytes forsteri*	Emperor penguin
*Aptenodytes patagonicus*	King penguin
*Eudyptes chrysocome*	Southern rockhopper
*Eudyptes filholi*	Eastern rockhopper
*Eudyptes chrysolophus chrysolophus*	Macaroni penguin
*Eudyptes chrysolophus schlegeli*	Royal penguin
*Eudyptes moseleyi*	Northern rockhopper
*Eudyptes pachyrhynchus*	Fiordland penguin
*Eudyptes robustus*	Snares penguin
*Eudyptes sclateri*	Erect-crested penguin
*Eudyptula minor*	New Zealand little penguin
*Eudyptula minor* Banks Peninsula	NZ white-flippered penguin (BAN)
*Eudyptula novaehollandiae*	Australian fairy penguin
*Megadyptes antipodes antipodes*	Yellow-eyed penguin
*Pygoscelis adeliae*	Adélie penguin
*Pygoscelis antarctica*	Chinstrap penguin
*Pygoscelis papua* West Antarctic Peninsula	Gentoo penguin WAP
*Pygoscelis papua* Falklands/Malvinas	Gentoo penguin FAL
*Pygoscelis papua* Kerguelen	Gentoo penguin KER
*Pygoscelis papua* South Georgia	Gentoo penguin SG
*Spheniscus demersus*	African penguin
*Spheniscus humboldti*	Humboldt penguin
*Spheniscus magellanicus*	Magellanic penguin
*Spheniscus mendiculus*	Galápagos penguin
*Megadyptes antipodes richdalei*	Chatham Islands *Megadyptes* penguin
*Megadyptes antipodes waitaha*	Waitaha penguin
*Eudyptes warhami*	Chatham Islands crested penguin

## Results

### Climate change drove evolution, biogeography, and demography

Phylogenetic results (Fig. 1 and Supplementary Fig. 2) confirm previous findings, recovering *Aptenodytes* (king and emperor penguins) as the sister clade to all other crown penguins, with brush-tailed (*Pygoscelis*) penguins in turn sister to two clades uniting the banded (*Spheniscus*) and little (*Eudyptula*) penguins and the yellow-eyed (*Megadyptes*) and crested (*Eudyptes*) penguins^6,7,9^. Biogeographical reconstructions (Fig. 1, Supplementary Figs. 3–4 and Supplementary Data 1) support a Zealandian origin for penguins^6,7^. Stem penguins radiated extensively in Zealandia before dispersing to South America and Antarctica multiple times, following the eastward-flowing direction of the Antarctic Circumpolar Current (ACC) (Fig. 1). Crown penguins most likely arose from descendant lineages in South America, before dispersing back to Zealandia at least three times. Interestingly, at least two such dispersals occurred before the inferred onset of the ACC system, suggesting that early stem penguins were not dependent on currents to disperse over long distances. A second pulse of speciation coincides with the onset of the ACC, though understanding whether this pattern is real or an artifact of fossil sampling requires more collecting from early Eocene localities. We infer an age of ~14 Ma for the origin of crown penguins, which is more recent than the ~24 Ma age recovered in genomic analyses, not including fossil taxa^7^ (Supplementary Fig. 2b) and coincides with the onset of global cooling during the middle Miocene climate transition^4,10^ (Supplementary Fig. 3a). This young age suggests that expansion of Antarctic ice sheets and the onset of dispersal vectors such as the Benguela Current^11^ during the middle to late Miocene facilitated crown penguin dispersal and speciation, as hinted at by fossil evidence^12^.

**Fig. 2:**
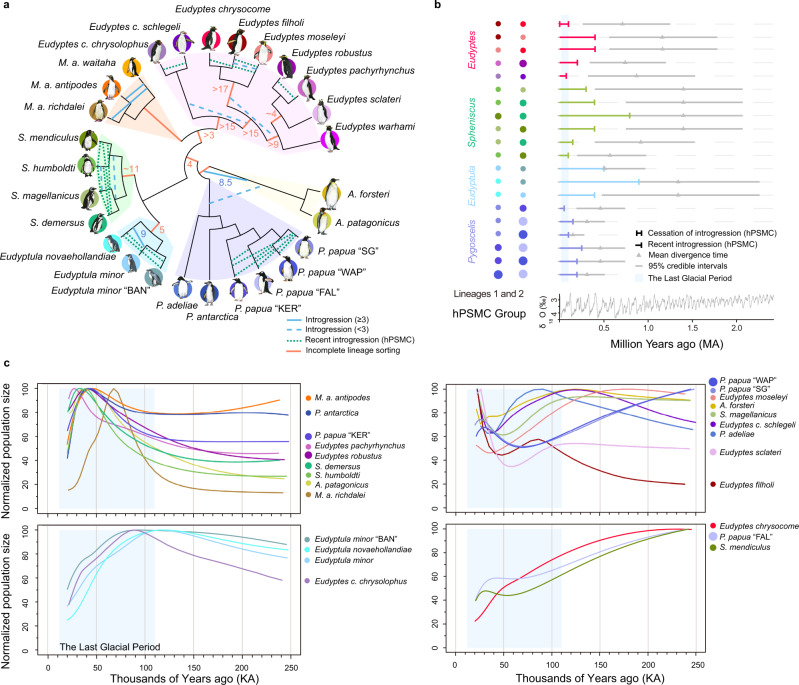
Incomplete lineage sorting, introgression events, and demographic history among penguins. **a** Model of incomplete lineage sorting (ILS) and introgression events estimated from QuIBL and hybrid pairwise sequentially Markovian coalescent (hPSMC) results. hPSMC was only run for 20 species pairs (see **b**). Numbers on branches represent the proportion (%) of ILS (orange branches) or introgression (blue lines, blue dashed lines, and blue dotted lines) detected by QuIBL. Proportions <3% were marked with blue dashed lines. Aqua dotted lines represent the ongoing gene flow detected by hPSMC. **b** Estimated divergence times and time intervals during which gene flow ceased between closely related lineages (see Supplementary Data 2 for details). Each circle represents one species in plot a. Gray lines represent the 95% credible intervals for the divergence times from the Bayesian total-evidence dating tree. Composite oxygen stable isotope (δ^18^O) data were modified from^10^ to show the climate fluctuations. **c** Normalized inferred population size (as a percentage of the maximum population size for each species between 20 and 250 Kya) trends for four groups of species showing similar patterns during the LGP based on PSMC results (full PSMC results shown in Supplementary Fig. 11). WAP = West Antarctic Peninsula, SG = South Georgia, KER = Kerguelen, FAL = Falkland/Malvinas and BAN = Banks Peninsula. Source data is provided as a Source Data file.

Incongruences between species trees and gene trees were identified, e.g., alternate topologies occurred at high frequencies (>10%) for several internal branches (Fig. 1c; Supplementary Fig. 5). These patterns indicate that gene tree discordance may be caused by incomplete lineage sorting (ILS) or introgression events. By quantifying ILS and introgression via branch lengths from over 10,000 gene trees, we found that the rapid speciation within crown penguins was accompanied by >5% ILS content within the ancestors of *Spheniscus*, *Eudyptula*, *Eudyptes,* and several subgroups within *Eudyptes* (Fig. 2a). Our dated tree provides a temporal framework for this rapid radiation: the four extant *Spheniscus* taxa are all inferred to have split from one another within the last ~3 Ma, and likewise the nine extant *Eudyptes* taxa likely split from one another in that same time (Fig. 1b). Many closely related penguin species/lineages are known to hybridize in the wild (see supplementary methods). Consistent with this, multiple analyses suggest that introgression also contributes to species tree—gene tree incongruence (Supplementary Figs. 6–9 and Supplementary Data 2; also see Supplementary Methods for further details). This could explain the most notable conflict in previous phylogenetic results, which showed inconsistency over whether *Aptenodytes* alone^7^ or *Aptenodytes* and *Pygoscelis* together^4,5^ represent the sister clade to all other extant penguins. Introgression was detected between the ancestor of *Aptenodytes* and the ancestor of other extant penguins, and is inferred to have occurred when the range of these ancestors overlapped in South America (Fig. 2a and Supplementary Data 2). Introgression (>9%) was also detected between *Eudyptula novaehollandiae* and *Eudyptula minor*, and several introgression events were especially pervasive in *Eudyptes* (Fig. 2a and Supplementary Fig. 6).

Many extant penguin lineages began to diverge within the last 3 Ma (Fig. 1). To obtain insight into this recent phase of penguin diversification, we inferred post-speciation introgression events and estimated the time when gene flow from introgression ceased between 20 pairs of closely related lineages (see Supplementary Methods). Our results provide further evidence for recent introgression between all sampled pairings (Fig. 2b) except for *Eudyptes chrysocome* and *E. filholi*, whose ranges are geographically disparate (Fig. 1a). Almost all species exhibit a genomic signature of a period of physical isolation during the Last Glacial Period (LGP) with increased climate fluctuation and environmental uncertainty, followed by postglacial contact and introgression as Earth warmed once again (Supplementary Figs. 8–9). This strongly supports the hypothesis that penguins were impacted by ecosystem-wide, climate-driven refugia/recolonization cycles in the Southern Ocean^13,14^, a pattern also observed in other marine taxa during the Last Glacial Maximum (e.g.,^15^). As ice volumes increased during the LGP high-latitude penguin species were likely forced into isolated mid-latitude refugia. As climate warmed from the late Pleistocene to Holocene, these species moved back towards the poles, recolonizing landmasses and islands as they became habitable once again, and, notably, experiencing secondary contact with one another (e.g., on small sub-Antarctic islands).

Today, penguins are under threat from climate change and environmental disruption (see Supplementary Methods for further citations) and half of all extant species are considered either Endangered or Vulnerable (IUCN red list categories). Understanding how past climate events have impacted penguin population size during the LGP is crucial in inferring how penguin populations may respond to future climate change. We estimated the effective population size for all recent penguin taxa except for *E. warhami* and *M. a. waitaha* (where data were too limited, Supplementary Data 2) (Fig. 2c, Supplementary Figs. 10–11 and Supplementary Data 2). These analyses provide a window into long-term population histories (very recent trends cannot be accurately recovered with these methods^16^). Four demographic patterns emerge for this critical time interval, illuminating disparate responses of penguins to glacial-interglacial cycles (Fig. 2c). The most prevalent pattern is shared by nine lineages (*Aptenodytes patagonicus*, *Pygoscelis antarctica, P. papua* “KER”, *S. demersus, S. humboldti M. a. antipodes*, *M. a. richdalei*, *Eudyptes robustus* and *E. pachyrhynchus*), all of which show evidence of population expansion coincident with the beginning of the LGP, followed by population decline towards the end of the LGP. In contrast to this pattern, nine lineages (*A. forsteri, P. adeliae, P. papua* “WAP”, *P. papua* “SG”, *S. magellanic*us, *E. moseleyi*, *E. filholi*, *E. chrysolophus schlegeli*, and *E. sclateri*) show evidence of population decline coincident with the beginning of the LGP, followed by population expansion towards the end of the LGP. Almost all of the remaining lineages show strong evidence of persistent long-term declines in populations from the early LPG to the end of LPG. All three *Eudyptula* taxa and *Eudyptes chrysolophus chrysolophus* underwent a steep population decline spanning the LGP, while three taxa (*P. papua* “FAL”, *S. mendiculus,* and *E. chrysocome*) show evidence of continual population decline across the last 250 thousand years (ka).

Interestingly, taxa that increased in population size towards the end of the LGP (e.g., *A. forsteri, P. adeliae, S. magellanicus, E. filholi, E. moseleyi, E. sclateri,* and *E. schlegeli* are typically migratory, and tend to forage offshore (>50 km; see Supplementary Data 1^17^), while taxa that decreased towards the end of the LGP (e.g., *S. humboldti, S. demersus, M. a. antipodes* and likely *M. a. richdalei*) tend to be residential, and forage inshore; see Supplementary Data 1. Taxa that disperse farther may have overcome local impacts of global climate cooling during the LGP (e.g., changes in sea-ice extent, prey abundance and terrestrial glaciation, however see^18^) largely by relocating to lower latitudes (e.g.,^14^), whereas locally-restricted taxa may have been more prone to sudden population collapses.

### Penguins have the slowest evolutionary rates among birds

**Fig. 3:**
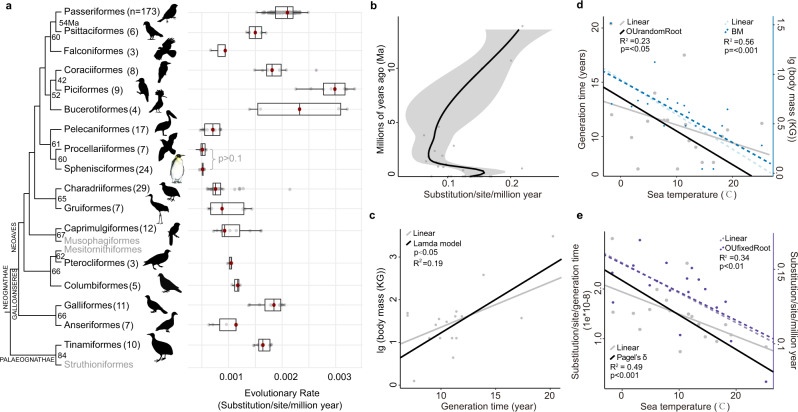
Evolutionary rates in birds. **a** Evolutionary rate in avian orders based on a ~19 Mbp alignment of highly conserved genome regions. Sphenisciformes and Procellariiformes have the lowest evolutionary rate among modern bird orders (One-sided Wilcoxon Rank sum test, *P* values < 0.05 for all pairs except for Sphenisciformes and Procellariiformes (*P*-values > 0.1)). Numbers at the tips represent the sample size in each group. Numbers at nodes represent the divergence times (Ma) between each order and its sister taxon and red dots within the boxplots indicate average values. We did not attempt to estimate the evolutionary rates for orders containing less than three sampled species (gray font; Musophagiformes, Mesitornithiformes, and Struthioniformes). Boxplots show the median with hinges at the 25th and 75th percentile and whiskers extending 1.5 times the interquartile range. Some bird images were downloaded from phylopic.org and were licensed under the Creative Commons (CC0) 1.0 Universal Public Domain Dedication. **b** Evolutionary rates inferred for extant penguin lineages at internal nodes from the maximum clade credibility tree, calculated using a 500 Mbp genome alignment. Gray shadows represent the 95% credible intervals. **c**–**e** Correlations between **c**, body mass and generation time (*P* value < 0.05), **d** generation time (gray dots, solid lines, *P* value < 0.001) or body mass (blue dots, dashed lines *P* value < 0.05) and average sea surface temperature, **e** substitution per site per generation time (gray dots, solid lines, *P* value < 0.001) or substitution per site per million years (purple dots, dashed lines *P* value < 0.01) and body mass among 18 penguins, estimated using phylogenetic correlation - Phylogenetic Generalized Least Squares Regression with the best-fitting model identified by Akaike Information Criterion. Correlations with linear models were shown with black lines. Source data is provided as a Source Data file.

The integrated evolutionary speed hypothesis (IESH) proposes that temperature, water availability, population size, and spatial heterogeneity influence evolutionary rate^19^. Life history traits also impact the evolutionary rate, but such relationships remain incompletely understood in birds^20^. Penguins are long-lived, large-bodied, and produce few offspring, thus providing an ideal case study in how life history may impact evolutionary rate. We tested the IESH using three proxies for evolutionary rate: substitution rate, P and K2P distances between lineages and their ancestors (Supplementary Fig. 12 and Supplementary Data 3). We found that penguins and their sister group (Procellariiformes) had the lowest evolutionary rates of the 17 avian orders sampled by^21^ (Fig. 3a, Supplementary Fig. 13, and Supplementary Data 3). Because other aquatic orders also show slow rates (e.g., the aquatic Anseriformes show a significantly slower rate than their terrestrial sister group Galliformes), we hypothesize that the rate in penguins represents the culmination of a gradual slowdown associated with increasingly aquatic ecology. Intriguingly, we detected a trend toward decreasing rate over the first ~10 Ma of crown penguin evolution, followed by a marked uptick ~2 Ma, which suggests the onset of glacial-interglacial cycles contributed to a recent increase in evolutionary rates in penguins (Fig. 3b).

Extant penguin lineages show a wide range of individual rates, and phylogenetic correlation analyses (phylogenetic generalized least squares regression) shed light on potential factors influencing this disparity (Fig. 3c–e and Supplementary Data 3). Extant penguins showed a significant negative correlation between body mass and average sea surface temperature (Fig. 3d). Despite species from warmer regions having shorter generation times (Fig. 3d), a significant negative correlation was found between evolutionary rate and average sea surface temperature (Fig. 3e), suggesting that temperature may influence penguin evolutionary rates by regulating selective pressures, but not only through its effect on metabolism^22^. This result is in parallel with studies that show speciation rates to be higher in polar environments than in the tropics, pointing towards faster rates of evolution and more opportunities for divergence at high latitudes^23,24^. We propose that these patterns together reflect the signature of climate oscillations on high latitude species: polar penguins (e.g. *A. forsteri*/*P. adeliae*) were likely forced into more northerly refugia during ice ages, subsequently recolonizing Antarctica during interglacials^14^. These events may have led to faster evolutionary rates as these lineages underwent population contraction-expansion cycles and were periodically forced to adapt to new environments.

### Putative molecular adaptations unique to penguins

**Fig. 4:**
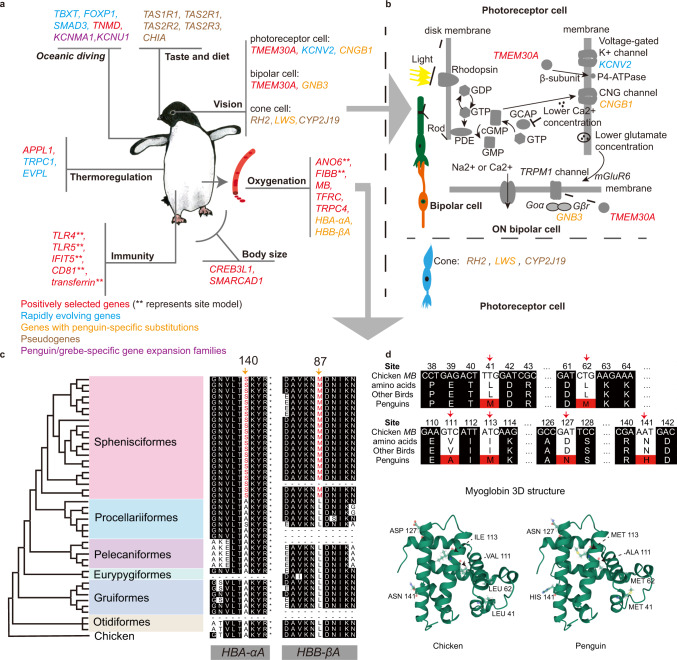
Adaptive genes in extant penguin lineages. **a** Genes with unique evolutionary signals in penguins and their putative adaptive function. **b** Gene regulatory pathways related to light transmission. **c** Phylogenetic tree of 45 avian species showing two mutation sites (*HBA-αA*, A140S, and *HBB-βA*, L87M) of hemoglobin genes in penguins (marked in red) and outgroups. **d** Positive selection at multiple sites (41, 62, 111, 113, 127, 141) on the bLCA of extant penguins for *MB* gene and the structural effects of amino acid substitutions in the chicken *MB* gene. Molecular models of the chicken *MB* gene and the *MB* gene with penguin-specific substitutions may affect the stabilization of *MB*. Source data is provided as a Source Data file.

**Fig. 5:**
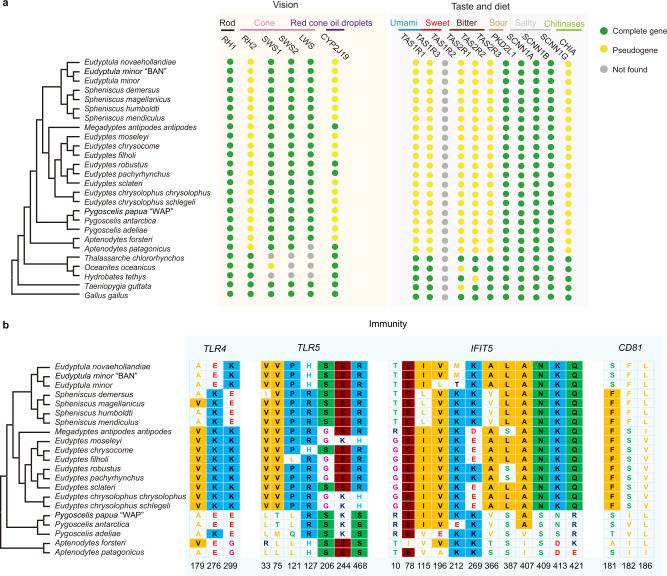
Pseudogenes and site alignments for vision, taste, diet, and immunity genes. **a** Presence/absence of vision, taste, and dietary genes in penguins. Phylogenetic tree of penguins and select outgroups indicate which species have complete or pseudogenes, related to vision (opsins; *RH1*, *RH2*, *SWS1*, *SWS2*, *LWS,* and *CYP2J19*), taste (umami; *TAS1R1*, *TAS1R3*, sweet; *TAS1R2*, bitter; *TAS2R1*, *TAS2R2*, *TAS2R3*, sour; *PKD211*, salty; *SCNN1A*, *SCNN1B*, *SCNN1G*) and diet (chitinase; *CHIA*). “Not found” indicates genes that could not be assembled. **b** Phylogenetic tree of penguins showing alignments of positively selected sites for four genes related to immunity (*TLR4*, *TLR5*, *IFIT5,* and *CD81*). Sites are shown below the alignment. The background colors are displayed for sites that have 50% conservation. Source data is provided as a Source Data file.

As penguins became increasingly adapted to a flightless diving ecology, they encountered novel selection pressures that required modifications to their locomotory strategy, thermoregulation, sensory perception, and diet. We tested whether these phenotypic changes have been facilitated through the evolution of the underlying protein-coding genes (Supplementary Data 4) by identifying positively selected genes (PSGs), rapidly evolving genes (REGs), and pseudogenes that relate to specific adaptations including thermoregulation, oceanic diving, oxygenation, underwater vision, shifts in diet and taste, body size and immunity (see Figs. 4, 5 and Supplementary Methods for additional details and citations). These genes either differ in all penguins compared with other birds, differ in the genus *Aptenodytes* compared with other penguins, or are under distinct selective pressures within penguins (Supplementary Data 4). In the branch leading to the last common ancestor (bLCA) of penguins, 27 PSGs (false discovery rate [FDR] *q* < 0.05) and 13 REGs (FDR *q* < 0.05) were detected. In the bLCA of *Aptenodytes*, 25 PSGs (FDR *q* < 0.05) and 3 REGs (FDR *q* < 0.05) were detected. In the bLCA of penguins and four flightless/nearly flightless birds (*Nannopterum harrisi*, *Rhynochetos jubatus*, *Zapornia atra,* and *Laterallus rogersi*, see Supplementary Fig. 16a), five PSGs (FDR *q* < 0.05) and 38 REGs (FDR *q* < 0.05) were detected. Within penguins, 275 PSGs (FDR *q* < 0.01) were detected (Supplementary Data 4). We related the gene pathways and known functions of 15 PSGs and six REGs to penguin-specific adaptations (Fig. 4a). We also highlight five genes containing penguin-specific substitutions, seven pseudogenes, and two gene expansions (Fig. 4a, Supplementary Figs. 14, 15).

We identified three REGs that are shared by penguins and other flightless/nearly flightless birds. These genes are likely associated with the shortening, rigidity, and increased density of the forelimb bones which contribute to the flipper-like wing of penguins (Fig. 4a). *TBXT* and *FOXP1* are related to the development of articular cartilage, tendons, and limb bones^25,26^. *SMAD3* is involved in the transforming growth factor-beta signaling pathway, which is important for maintaining articular cartilage and stimulating osteogenesis and bone formation^27^. Perhaps most interestingly, *TNMD*, a PSG, is expressed during the differentiation and developmental phase of limb tendon, ligament, and collagen fibrils, and loss of *TNMD* can result in reduced tenocyte density^28^. We hypothesize that *TNMD* may be key to the nearly wholesale replacement of penguin distal wing musculature by tendons, which stiffens and reduces heat loss to the high surface area flipper (Supplementary Fig. 16a-d). We also identified two genes *KCNU1* and *KCNMA1* that are related to calcium sequestration to be expanded in the genomes of both penguins and grebes (*Podiceps cristatus* and *Podilymbus podiceps*) (Fig. 4a, Supplementary Fig. 15). These genes likely contribute to the high bone density characteristic of these taxa, which helps reduce buoyancy for deep diving.

Penguins have densely-packed waterproof feathers, thick skin, and a layer of subcutaneous fat enabling them to thermoregulate in cold environments. We identified four genes under selective pressure in common ancestors of penguins that are related to thermoregulation (Supplementary Data 4). These genes (*APPL1*, *TRPC1*, *EVPL*) showed evidence of positive selection or rapid rates of evolution on the bLCA of extant penguins but not in other birds (Fig. 4a). The white adipose tissue of penguins is important for survival in the cold, acting as an insulative layer and an energy reserve, particularly prior to catastrophic moult^29^. We hypothesize several of these genes contribute to white adipose fat storage and hence survival in cold environments. *APPL1* (Supplementary Fig. 16e) and *TRPC1* are related to glucose levels and fatty acid breakdown through adiponectin^30,31^.

Penguins function under hypoxic conditions during deep dives in part via myoglobin concentration and utilizing anaerobic metabolism^32,33^. We identified seven genes related to oxygenation that are under positive selection or have penguin-specific substitutions in penguins. Transferrin Receptor 1 (*TFRC)* shows a positive selection in penguins (Supplementary Fig. 16f). Previous experimental work in cells has reported that *TFRC* messenger RNA is expressed in an oxygen-dependent manner^34^. Importantly, *TFRC* is a top candidate gene for the hypoxia response of domesticated cattle^35^. We hypothesize that *TFRC* has contributed to a convergent adaptation to withstanding hypoxia in penguins. Interestingly, *FIBB* and *ANO6*, which are involved in blood coagulation, showed a signal of positive selection in *Aptenodytes*, but not in other genera (Supplementary Fig. 17). Among all penguins, *Aptenodytes* have the capacity for the deepest diving (>500 m depth)^36^, and thus, these gene variants may enable these species to dive to extreme depths. While none of the hemoglobin genes were PSGs (P-value: >0.05), we observed that *HBA-αA* (A140S) and *HBB-βA* (L87M) genes (Fig. 4c and Supplementary Fig. 18) show penguin-specific amino acid substitutions that are highly conserved across all penguin species, making them candidate molecular adaptations for surviving deep oceanic dives under hypoxic conditions (see also ref. ^37^). *MB* is an oxygen-binding myoglobin gene that shows positive selection at multiple sites both between penguins and other birds and among penguins (Fig. 4d and Supplementary Fig. 16g), suggesting that these penguin-specific substitutions may impact the stability of the resulting myoglobins, as seen in extreme deep-diving cetaceans^38^. While cormorants and petrels also undertake deep (>70 m) dives, we did not observe selection for *TFRC* and hemoglobin genes in these groups (Fig. 4c). Another PSG, *TRPC4*, is involved in the cardiovascular system^39^. Specifically, *TRPC4* may help widen blood vessels to decrease blood pressure during deep dives^40^.

Penguins frequently forage in low light, and exhibit specializations for vision in dim, blue-green marine environments^41,42^. Morphological research has shown that at least some penguins are cone trichromats with only three functional cone photoreceptor types, blue-shifted long-wavelength visual pigments, and no red oil droplets^41^. Genomic data support trichromatism in all penguins, in contrast to most other birds which are tetrachromats. The inactivation of the green cone opsin gene (*RH2*) in the stem penguin lineage is inferred by a 12-base pair (bp) deletion, which encompasses the codon for the critical chromophore-binding lysine (K296^43^) (Fig. 4a and Supplementary Fig. 19a). As all penguins share this deletion, reduced color vision must have occurred in the penguin stem lineage, similar to secondarily aquatic mammals^44^. Although penguins lack green cones, the functional orthologs of the remaining visual opsins in penguins strongly indicate the retention of violet (*SWS1*), blue (*SWS2*), and red (*LWS*) cones, plus rods (*RH1*) (Fig. 5a). This genetic signature is concordant with our experiments on *Pygoscelis papua* (see Supplementary Methods), which demonstrate a capacity for ultraviolet light perception at 365 nm, likely conferred by the SWS1 opsin. Furthermore, the peak wavelength sensitivity (λ_max_) of penguin *LWS* opsins show evidence of shifts in spectral sensitivity to better match ambient underwater light. Relative to key avian model species (e.g., *Taeniopygia guttata*, *Columba livia*, *Gallus gallus*) and Procellariiformes, penguins possess substitutions at five key tuning sites in *LWS*, four of which (A180, F277, A285, and S308) are associated with blue-shifting this pigment^45^ (Supplementary Fig. 19b). This suggests that this opsin has been fine-tuned for marine foraging, as observed in cetaceans^44^. *CYP2J19*, which encodes a carotenoid ketolase responsible for producing red oil droplets in avian cones^46^, has been inactivated in most penguins (Supplementary Data 4). Colored oil droplets are thought to fine-tune color vision^46^, though this comes at the cost of decreased visual sensitivity. Deactivation of *CYP2J19* likely allows for higher retinal sensitivity when foraging in dim light conditions, as seen in nocturnal owls and kiwis^46^. Beyond these key genes, we note that two scotopic photoresponse genes, *TMEM30A* (PSG) and *KCNV2* (REG), show evidence of selection in penguins, and two others, *CNGB1* and *GNB3*, each have a site mutation unique to penguins (Supplementary Fig. 19c, d). These genes play an important role in the transmission of light (Fig. 4b), and may further enhance visual sensitivity at low light levels, as mutations or loss of these genes impact the result in a reduced scotopic photoresponse^47,48^.

A wholesale reduction in gustation capacity appears to have accompanied the shift to underwater prey capture and consumption in penguins. We verified that penguins only retain genes associated with detecting sour and salty tastants, and lack functional copies of genes linked to umami, sweet and bitter tastants^49^ (Figs. 4a and 5a). The mutational loss of capacity for umami taste in penguins is puzzling, given the continued consumption of amino acid-rich prey. Intriguingly, the loss of umami has also been reported in secondarily aquatic mammals^50^. Potential explanations include a lower reliance on taste when swallowing food whole or weakened ability to taste prey due to cold temperatures and the sodium content of seawater (reviewed in^50^).

A strong genomic indicator of diet is presented by chitinases that are expressed in the gastrointestinal tract^51^. The chitinase genes (*CHIA*s) exist as several paralogs, and the retention or loss of these paralogs in mammals has been correlated with diet^51^. Retention of intact *CHIA*s correlates with a higher degree of insectivory, and *CHIA* losses tend to occur in lineages that undergo dietary shifts to carnivory or herbivory. We examined *CHIA*s in penguins, and in contrast to most examined birds, which have one to four intact *CHIA*s^52^, penguins have a single pseudogenized *CHIA*. At first glance, it is perplexing that penguins would lose *CHIAs*, as many species consume large amounts of crustaceans. Fossil evidence, however, reveals that stem penguins focused primarily on larger prey items like fish and squid, and that adaptations for capturing smaller planktonic prey arose as recently as the Pliocene^6^. We propose that the two inactivating mutations shared by extant penguins (Fig. 5) evolved during a ~50 Ma interval during which stem penguins consumed little or no arthropod prey.

Co-evolution between hosts and pathogens is pervasive in vertebrates. Given the range of different climatic niches occupied by penguins, and the differences in pathogen assemblages to which they are undoubtedly exposed, penguins may have undergone significant adaptation to local pathogen pressures^53^. Accordingly, we detected 51 PSGs in penguins that have a role in immunity (Supplementary Data 4). Several of these genes might be under positive selection corresponding to host-pathogen co-evolution. For instance, we confirm previous reports^53,54^ that the bacterial-recognizing Toll-like receptors *TLR4* and *TLR5* (Figs. 4a and 5b) are positively selected in penguins. Moreover, the positively selected sites located proximal (<5 Å) to the lipopolysaccharide-binding site in *TLR4* (codon 276, homologous to chicken codon 302^55^) and at a flagellin-binding site in *TLR5* (codon 33^56^) (Fig. 5b) are both in domains crucial for bacterial recognition. In addition, we detected several other pattern-recognition receptors, such as *IFIT5*, that are also under positive selection in penguins (Fig. 4a). *IFIT5* is a cellular detector of viral RNA^57^, and we found a cluster of positively selected sites located in a connecting helix forming part of the RNA-binding cleft (codons 407, 409, 413, and 421, corresponding to human codons 412, 414, 418 and 426^58,59^) (Fig. 5b). This may imply that penguin *IFIT5* has undergone adaptation to different viral RNA motifs in response to viral pathogen pressure. We also found evidence of positive selection at viral targets of cell entry. For example, *CD81* is a co-receptor required for glycoprotein-mediated hepatitis C viral entry into cells in mammals^60^, and positive selection has been reported at the glycoprotein interface in bat *CD81*^61^. We also found a cluster of positively selected sites in the hepatitis C glycoprotein interface in penguin *CD81* (sites 181, 182, and 186, corresponding to human sites 180, 181, and 185, and penguin site 86, corresponding to human site 185) (Fig. 5b). This may suggest that penguins have experienced co-evolution with a viral pathogen that relies on *CD81* for cell entry. Finally, we detected positive selection in penguin transferrin, which is part of the “nutritional” immune system that sequesters iron from iron-scavenging pathogens^62^. Outbreaks of diphtheritic stomatitis in *Megadyptes antipodes* have caused increasing chick mortality and are hypothesized to be related to increasing susceptibility to *Corynebacterium* as a secondary infection^63^ potentially triggered by chick malnutrition due to changes in diet, and potentially iron intake. The co-evolutionary arms race to sequester and scavenge iron has also been detected in mammals and fishes (e.g.,^64^). Taken together, these observations illustrate that immune genes have undergone diversification in penguins. Furthermore, many positively selected sites were clustered in regions known to be involved in pathogen binding, which provides evidence for extensive host-pathogen co-evolution during the diversification of penguins into novel pathogen environments.

Extant penguins range from ~1 kg in *Eudyptula* spp. to 40 kg in *Aptenodytes forsteri*, but giant fossil penguins exceeded 100 kg^65^. We found two genes associated with large body size that are under positive selection in *Aptenodytes* compared to all other penguin lineages (Fig. 4a). *CREB3L1* is important during bone development, and vertebrates lacking *CREB3L1* have underdeveloped growth^66^. *SMARCAD1* is related to the skeleton and plays a role in transcriptional regulation, maintenance of chromosome stability, and various aspects of DNA repair. Vertebrates with mutant *SMARCAD1* also have underdeveloped growth^67^. We hypothesize that these genes have contributed to the large body size of *Aptenodytes*. Although genetic data are inaccessible for stem penguins, the recovery of *Aptenodytes* as sisters to all other extant penguins and the large size of many stem penguins (e.g., *Kumimanu* and *Kairuku*) suggests positive selection in these genes could be ancestral for crown penguins with selection relaxed in non-*Aptenodytes* taxa.

## Discussion

Our comprehensive study encompassing all extant and many fossil penguins provides a new window into the processes that have shaped >60 Ma of evolution. Our phylogenomic analyses confirm the Zealandian origin of penguins, extensive radiation before dispersal to South America and Antarctica, and the second pulse of speciation at the onset of the ACC. Our study reveals new evidence that penguin speciation events were driven by changes in global climate and oceanic dispersal, leading to allopatric speciation across the Southern Hemisphere. Recent speciation in *Eudyptes*, *Megadyptes, Spheniscus,* and *Eudyptula* has been rapid, with a complicated history of gene flow and ILS that make species boundaries within these taxa difficult to untangle (e.g.,^5,14^). Importantly, the mechanisms that have shaped penguin diversification in the past (e.g., development of major current systems, geological uplift of oceanic islands) remain important for taxa that appear to still be in the process of speciation today (e.g., within *Pygoscelis papua* and between *Eudyptes chrysolophus chrysolophus/E. C. schlegeli*, *E. pachyrhynchus/E. robustus*, *E. chrysocome/E. filholi/E. moseleyi,* and *Spheniscus* spp.^5,14,68^).

By comparing our penguin genomes to >300 other avian genomes, we demonstrate that penguins and Procellariiformes have the lowest evolutionary rates observed among birds to date. These low evolutionary rates seem to belie the profound adaptations penguins show for a secondary aquatic existence, but a synthetic reading of the fossil record and the genomic data suggests that penguins rapidly acquired many of the key features associated with their aquatic life very early in their diversification and rates of change slowed towards the present. Genomic signals of molecular adaptations with evidence of positive selection or penguin-specific substitutions were identified in a variety of genres, including genes associated with oceanic diving, thermoregulation, oxygenation, underwater vision, taste, and immunity. Though the overall evolutionary rate in penguins is slow, we identified higher evolutionary rates in crown penguin ancestors than in extant penguins and shifts in rates in individual lineages over the past 14 Ma.

While evolutionary rates and sea surface temperatures appear to be negatively correlated, evolutionary rates and body mass are positively correlated, suggesting that large-bodied species inhabiting colder climates are more equipped to adapt to new environments during climate events. Indeed, our demographic results reveal that penguins have had a complicated history, shaped by climatic oscillations, which has led to population crashes in those species reliant on restricted niches and ecologies. Genomic evidence highlights how some penguin populations collapsed during previous climatic shifts^13,14^, and the risks of future collapses are ever-present as penguin populations across the Southern Hemisphere are faced with rapid anthropogenic climate change^69^. While our analyses suggest that ocean temperature may regulate certain selection pressures, the current pace of warming combined with limited refugia in the Southern Ocean will likely far exceed the adaptive capability of penguins^70^. Over 60 Ma these iconic birds have evolved to become highly specialized marine predators, and are now well adapted to some of the most extreme environments on Earth. Yet, as their evolutionary history reveals, they now stand as sentinels highlighting the vulnerability of cold-adapted fauna in a rapidly warming world.

## Methods

### Genome sequencing, assembly, and annotation

We analyzed 27 genomes comprising all extant and recently-extinct penguin species, subspecies, and major lineages. 21 of the high-coverage genomes have been published by members of our consortium for this project^8,9^. To supplement the dataset, we sequenced three high-coverage genomes from the remaining *Pygoscelis papua* lineages from Falkland Islands/Malvinas “FAL”, Kerguelen Island “KER” and South Georgia “SG” (see^68^), and partial genomes from the recently-extinct *Eudyptes warhami*, *M. a. richdalei* and *M. a. waitaha* (see ref. ^5^ and citations within). See Supplementary Methods for more detail on sample collection, extraction, sequencing, assembly and sex chromosomes. As such, we present the most comprehensive genomic dataset spanning all modern penguins, and to the best of our knowledge, present the first genomic dataset encompassing an entire multi-species vertebrate order. To compare our penguin genomes to other bird genomes, we obtained 361 bird genomes recently released by^20^ as part of the B10K project (https://b10k.genomics.cn), representing 36 orders and 218 families.

### Additional data on modern and fossil penguins

We expanded the morphological dataset of^6^ by incorporating additional fossil penguin species and seven additional characters. The final matrix comprised 72 fossil and extant penguin taxa, two outgroup taxa, and 281 morphological characters (Supplementary Data 5). The average sea surface temperatures were obtained from spot locations from each lineage (Supplementary Data 3). Generation times of each extant lineage were obtained from the IUCN. For *M. a. richdalei* we used the *M. a. antipodes* generation time (Supplementary Data 2) (see Supplementary Methods).

### Phylogenomic inference and divergence time estimation

We combined all penguin genomes with the morphological matrix to resolve the timing and drivers of >60 million years of penguin evolution. In doing so, we update previous phylogenies (e.g., ^4,5,7,9^) to include genomes and morphology from all penguin taxa, including all major *P. papua* lineages and recently-extinct taxa. To explore the diversification of penguins, we undertook multiple phylogenomic analyses encompassing different subsets of taxa (Fig. 1, Supplementary Figs. 2–4 and Supplementary Software).

We aligned and merged our genomes to the 363-bird alignments from the B10K project^20^. The final alignments were extracted and multiple hits were filtered out for downstream analyses. We then created four alignments accounting for different subsets of taxa: (1) all putative species, subspecies and lineages (27 penguin taxa + 5 outgroups in total); (2) all extant lineages (24 penguin taxa + 5 outgroups in total), removing *Eudyptes warhami*, *M. a. richdalei* and *M. a. waitaha* from the former alignment; (3) all putative species and subspecies, removing *P. papua* “FAL”, “KER” and “SG” lineages (21 penguin taxa + 5 outgroups in total); and (4) only putative species (19 penguin taxa in total), further removing *Eudyptula minor* “BAN” and *Eudyptes chrysolophus schlegeli*. We also created one large genome alignment with all 385-bird taxa (not including *Eudyptes warhami*, *M. a. richdalei* and *M. a. waitaha*) (see Supplementary Methods).

To verify the phylogenomic relationships of modern penguins, we ran coalescent-based and concatenation-based phylogenies accounting for the different subsets of taxa described above (see Supplementary Methods). The topology for all clades was strongly supported and identical using all methods (Supplementary Fig. 2 and Supplementary Data 5), except for the placement of *Eudyptes warhami* among *Eudyptes* lineages in a single phylogeny.

We estimated the divergence time between modern taxa using the calibration points in ref. ^5^ (Supplementary Data 5), except we removed *Pygoscelis calderensis* based on recent revisions of topology^7,9^. We also added a “Crown Procellariiformes” (which is a sister to penguins) calibration point to calibrate the divergence between albatross and storm petrels. We also added three tip dates for extinct taxa, using the fossils *Madrynornis mirandus*, *Spheniscus muizoni,* and the fossil specimen NMNZ S.046318 (*Eudyptes* sp.) (see Supplementary Methods). All trees shared the same topology with our initial analyses, with the exception of the placement of the extinct *Megadyptes antipodes waitaha*, and had similar divergence times with each other (Supplementary Fig. 2b) We then generated a Bayesian total-evidence dating tree using the fossilized birth-death process (Fig. 1), expanding^4^ by including more species, genome data, and updating the morphology. We also calculated the genetic distances between our modern penguin genomes (Supplementary Fig. 12).

### Ancestral range estimation

We estimated the ancestral distribution of penguins with the total-evidence dated phylogenomic tree and twelve models, expanding on^6,7^ and following^6^. We used ten geographical areas and six-time slices, and normalized distances against the shortest pairwise distance in the time slice in this analysis. We then undertook standard model-testing (Likelihood Ratio Test and Akaike information criterion) to identify the best-fitting model for our data. We also used a Biogeographical Stochastic Mapping method to account for the apparent dispersal/vicariance/etc events. See Supplementary Methods for more details.

### Quantifying introgression and ILS between taxa

Controversy still remains regarding taxonomic boundaries between some closely related penguin taxa (See Supplementary Methods for more details). We undertook multiple analyses to assess the discordance of gene trees and levels of ILS and introgression (Supplementary Data 6). We first calculated the frequency of gene tree discordance for each internal branch and summarized the topologies for three different gene tree data sets. We assessed levels of ILS and introgression by quantifying them via internal branch lengths between all species (Supplementary Software). We tested the direction of introgression among lineages and assessed what genomic regions have introgressed, by analyzing 16 five-species combinations with symmetric phylogenies (Supplementary Data 2). We also examined introgression, by selecting different taxa from different genera and some closely related lineages/species. Finally, we assessed the cessation of gene flow between six closely related penguin groups. See Supplementary Methods for more details.

### Demographic history of penguins

We undertook analyses of demographic history by profiling heterozygosity across each genome (Supplementary Fig. 10), and undertaking analyses of effective population size (*N*_*e*_) over the last 1 Ma. As the number of heterozygous sites for *M. a. waitaha* and *Eudyptes warhami* remained too low, we only present analyses for *M. a. richdalei*. We used the species divergence time tree as an estimation of the mutation rate and detailed the divergence times in Supplementary Data 2. We focussed on the last 500 Kya, a period encompassing dramatic glacial/interglacial cycles (see Supplementary Methods).

### Comparison of evolutionary rate

The evolutionary rate between penguins and other birds was compared using both genomic distance and rate comparisons (Supplementary Fig. 12). We calculated P and K2P distances between taxa following the formulas: *P* distance = *p* + *q* and K2P-distance = −1/2ln((1-2*p*-*q*)*sqrt(1-2*q*)). Here, *p* is the proportion of transitions while *q* is the proportion of transversions between two genomes. We also estimated the evolutionary rate of penguins using the substitution rate (substitution per site per year) = substitution per site/divergence time. The correlation relationship between the substitution rate and sea surface temperature for extant penguins was tested using a phylogenetic generalized least squares (PGLS) regression (Fig. 3 and Supplementary Data 3). We also conducted PGLS regression analysis to determine the correlation relationship between sea surface temperature and body mass or generation time (Supplementary Software). We also compared the genome size among birds to check whether the genome size has a correlation with the proportion of repeat elements (Supplementary Data 3). See Supplementary Methods for more details.

### Putative molecular adaptations

We undertook comparative genomic analyses across all extant penguin taxa to identify genes and regulatory changes contributing to the remarkable morphological and physiological variation within penguins. We do not include *Eudyptes warhami*, *M. a. richdalei,* and *M. a. waitaha* or additional *P. papua* lineages (“FAL”, “SG”, “KER”) in these analyses. Our analyses expand on previous analyses that have only examined *A. forsteri* and *P. adeliae* (e.g., ^8,49^), or those that have relied on only on-site analysis for penguins (e.g., ^7^).

To understand the adaptive evolution of specific phenotypes in the branch leading to the last common ancestor of penguins, we identified positively selected genes, rapidly evolving genes, and evolutionarily conserved genes for extant penguins under a branch model and a branch-site model (see Supplementary Methods). We obtained orthologous genes against the chicken genome for 44 bird species including penguins, retaining a total of 8716 high-confidence orthologous genes. These genes were used to conduct a multiple sequence alignment. We then detected positively selected genes/rapidly evolving genes in the branch leading to the last common ancestor of penguins and detected positively selected genes/rapidly evolving genes in the branches of the last common ancestor of penguins plus four flightless/nearly flightless birds (see Supplementary Methods for more details). Genes with a false discovery rate adjusted *P*-value less than 0.05 were treated as candidates for positive selection or rapid evolution (Supplementary Data 4). To reveal more characteristics in penguins, we predicted whether an amino acid substitution site may have an impact on the biological function of a protein, by comparing penguins to the 23 other birds, and scanning for premature stop codons in each gene alignment. We also examined specific genes individually. In addition, we annotated and undertook further qualitative comparisons of these genes identified in penguins with over 300 other avian species to explore what happens in other birds (Supplementary Data 7). See Supplementary Methods for more details. While transcriptional evidence to support adaptive inferences is highly important, such data remains unrealizable in our study due to cultural and ethical hurdles.

### Behavioral study of gentoo penguin vision

As a representative of penguins, we undertook a behavioral study on captive *P. papua* at SEALIFE Kelly Tarlton’s Aquarium, Auckland, New Zealand to examine their ability to see in the ultraviolet (UV) spectrum. A Tank007 TK566 black OEM 365 nm torch (Shenzhen Grandoor Electronic Co., Ltd., China) was projected onto the snow in the enclosure, and penguins were observed to determine whether they would follow the movements of the torch’s UV projection. At least five penguins appeared to be able to follow the torch’s projection. No such interest was displayed when the torch was turned off, demonstrating that *P. papua* are able to see in the near UV spectrum (Supplementary Movie 1).

### Reporting summary

Further information on research design is available in the Nature Research Reporting Summary linked to this article.

## Supplementary information

Supplementary Information

Description of Additional Supplementary Files

Supplementary Movie 1

Supplementary Data 1

Supplementary Data 2

Supplementary Data 3

Supplementary Data 4

Supplementary Data 5

Supplementary Data 6

Supplementary Data 7

Supplementary Software

Reporting summary

## Data Availability

The sequencing data and genome assemblies generated in this study have been deposited in the NCBI database under BioProject PRJNA722815 and PRJNA556735, as well as the CNSA of the CNGBdb database under the accession number CNP0000605. Appendix datasets (BioGeoBEARS results and PSMC results) have been deposited on Figshare [10.6084/m9.figshare.c.5535243.v1]. Supplementary data files and source data generated in this study are provided in the Supplementary Information and Source Data file. The following datasets were also used in this study: CNSA accession number CNP0000505, and NCBI Genbank accession number NP_990272, NP_001071646, NP_001071647. Source data are provided in this paper.

## Source data

Source data

## Code availability

Analyses were performed using open-source software tools and the detailed parameters for each tool are shown in the relevant methods in Supplementary Information. The custom scripts and codes used in this study are also available in Supplementary Software files.
