# Supplementary material for: Genomic insights into the secondary aquatic transition of penguins
Source: Nat Commun. 2022 Jul 19;13:3912. doi: 10.1038/s41467-022-31508-9 (PMC9296559; doi:10.1038/s41467-022-31508-9)
Supplement: Supplementary file 1 — Supplementary Information [file 41467_2022_31508_MOESM1_ESM.pdf]

SUPPLEMENTARY INFORMATION FOR

Genomic insights into the secondary aquatic transition of penguins

Theresa L. Cole[1,35,*], Chengran Zhou[2,35], Miaoquan Fang[2,35], Hailin Pan[2], Daniel T. Ksepka[3], Steven R. Fiddaman[4], Christopher A. Emerling[5], Daniel B. Thomas[6], Xupeng Bi[2,7], Qi Fang[2], Martin R. Ellegaard[8,9], Shaohong Feng[2,7], Adrian L. Smith[4], Tracy A. Heath[10], Alan J. D. Tennyson[11], Pablo García Borboroglu[12,13,14], Jamie R. Wood[15], Peter W. Hadden[16], Stefanie Grosser[17], Charles-André Bost[18], Yves Cherel[18], Thomas Mattern[17], Tom Hart[4], Mikkel-Holger S. Sinding[8], Lara D. Shepherd[11], Richard A. Phillips[19], Petra Quillfeldt[20], Juan F. Masello[20], Juan L. Bouzat[21], Peter G. Ryan[22], David R. Thompson[23], Ursula Ellenberg[13,24,25], Peter Dann[26], Gary Miller[27,28], P. Dee Boersma[12], Ruoping Zhao[29], M. Thomas P. Gilbert[8,9], Huanming Yang[2,30,31], De-Xing Zhang[32,33], Guojie Zhang[1,2,7,29,34,*]

[35]These authors contributed equally

*Correspondence: tesscole1990@gmail.com (T.L.C.), guojiezhang@zju.edu.cn (G.Z.)

THIS PDF FILE INCLUDES

Supplementary Methods

Supplementary Figures

Supplementary References

Description of Additional Supplementary Files

Supplementary Methods

Source organism

Pygoscelis papua blood was obtained from New Island, Falkland Islands/Malvinas (*n* = 2; Research License No: R15/2017; Date: 2018/2019 season), Pointe Morne, Kerguelen Island (*n* = 1; Collection permit and animal ethics permissions provided by the TAAF: Arrêté n° 2012-119 du 29 Octobre 2012 Autorisant la réalisation du programme 394 de l'IPEV, Oiseaux Plongeurs; Date: 2012-2013) and Bird Island, South Georgia (*n* = 2; Permit: GSGSSI permit, BAS 2001-02; Date: 2005/2006 season). Whole genomic DNA was extracted using the HiPure Blood DNA Midi Kit II at BGI (Hong Kong), and diluted to 20 µL by Tris-EDTA buffer. The quality and quantity of each DNA extraction was assessed by first estimating the concentration of 1µL DNA extraction on a Microplate Reader, and the DNA fragment size was evaluated by pulse gel electrophoresis.

Work by [1,2,3,4] comprising ancient DNA (short mitochondrial sequences, mitochondrial genomes) and skeletal comparisons of bones has uncovered three recently extinct penguins (*Eudyptes warhami*, *Megadyptes antipodes richdalei* and *M. a. waitaha*) from New Zealand and the New Zealand Chatham Islands. Based on morphology, locality and stratigraphic age, we took small subsamples (*M. a. richdalei*: S.29384.35; *M. a. waitaha*: S.42156.6; *Eudyptes warhami*: S.29384.37) of bone from specimens in the Museum of New Zealand Te Papa Tongarewa collection. To minimise further damage to the collection, the samples chosen were already damaged and were not type specimens. A permit to export the samples permanently was obtained from the New Zealand Ministry for Culture and Heritage. Samples were sent to the GLOBE Institute at the University of Copenhagen for ancient DNA extraction.

Genome sequencing, assembly and annotation

We analysed 24 high-coverage genomes and three partial genomes comprising all extant and recently extinct penguin species, subspecies and major lineages. 21 of the high-coverage genomes have previously been published by members of our consortium: *Aptenodytes forsteri* and *Pygoscelis adeliae* genomes have been available since 2014[5], and 19 genomes encompassing all extant species and subspecies were published as an early-release dataset for this project in 2019[6], spanning: *Aptenodytes patagonicus*, *Pygoscelis antarctica*, *P. papua* West Antarctic Peninsula ("WAP") lineage, *Eudyptes chrysolophus chrysolophus*, *E. c. schlegeli*, *E. moseleyi*, *E. filholi*, *E. chrysocome*, *E. pachyrhynchus*, *E. robustus*, *E. sclateri*, *Megadyptes antipodes antipodes*, *Eudyptula novaehollandiae*, *E. minor*, *E. minor* Banks Peninsula/white-flippered lineage ("BAN"), *Spheniscus demersus*, *S. magellanicus*, *S. humboldti* and *S. mendiculus*. To supplement our dataset, we also sequenced three high-coverage genomes from the remaining major *P. papua* lineages, comprising the Falkland Islands/Malvinas ("FAL"), Kerguelen Island ("KER") and South Georgia ("SG") as discussed in [7,8,9] as well as three partial genomes for all known recently extinct taxa, *Eudyptes warhami*, *Megadyptes antipodes richdalei* and *M. a. waitaha* described in [1,2]. We also used BUSCO (v. 3.0.2)[10] and Augustus (v3.3.1)[11] to evaluate the three assembled penguin genomes with the avian database aves odb9 (Supplementary Data 1). As such, we present the most comprehensive genomic dataset spanning all modern penguins, and to the best of our knowledge, present the first genomic dataset encompassing an entire multi-species vertebrate order.

***Pygoscelis papua* genomes**

We sequenced high-coverage genomes for the remaining three major lineages of *Pygoscelis papua*[7,8,9]. Following quality control, a single sample per lineage was chosen for genomic library construction. As the DNA was of high molecular weight, we constructed a single 10X Genomics genomic library for each lineage following the 10X Genomics protocol, and sequenced each library on a BGISEQ-500 to produce up to 100X coverage. Genomes were assembled using Supernova v2.0.0[12] and evaluated following [6] (Supplementary Data 1).

Extinct penguin genomes

Bone samples for the three extinct penguins, *Eudyptes warhami*, *Megadyptes antipodes richdalei* and *M. a. waitaha* were UV-radiated on two sides for 30 seconds, using Crosslinker 1000 (Fischer Scientific) to reduce surface contamination. Approximately 100 mg bone was retrieved from each sample using a Dremel drill and was subsequently crushed using a mortar into fragments <1 mm. Between work on each sample, the laminar flow bench and all tools were washed in 5% bleach solution and UV-radiated for 30 seconds. DNA was extracted from the crushed bone fragments utilizing a pre-digestion step as recommended by [13], with lysis buffer containing 0.45 M EDTA (pH 8.0) and 0.25 mg/ml proteinase K, rotating at 37°C for 15 minutes. The supernatant was then removed and 4 ml lysis buffer were added, rotating at 37°C for a further 24 hours. The DNA was purified using SPRI-beads and then eluted in 70 μl EBT buffer. One extraction negative was included. 32 μl of each DNA extraction was built into double-stranded libraries using a Blunt-End-Single-Tube protocol specifically designed for aDNA[14]. The extraction negative was included as one library negative. 10 μl of each library was amplified in 50 μl reactions and amplified using 15 – 21 cycles, using a 6 bp dual-indexing approach[15]. The optimal number of PCR cycles was determined by quantitative PCR (qPCR) (MxPro 3000). The amplified libraries were purified using SPRI beads and quantified on TapeStation 2200 using HS tapes. The amplified indexed sample libraries were then pooled in equimolar amounts and sequenced on the BGI platform DNBSEQ using SR 100 bp mode. Base calling was performed in-house at BGI. Only sequences with correct indexes were retained. Fastq files were processed using the PALEOMIX pipeline (v1.2.12)[16]. Adapters and low quality reads (Q <20) were removed using AdapterRemoval2 (v2.2.0)[17], retaining reads >25 bp length. Trimmed and filtered reads were mapped to the *Megadyptes antipodes antipodes* genome reference using BWA (v0.1.17)[18] with seed disabled to allow for better sensitivity as recommended for ancient DNA[19] as well as removing unmapped reads. Only reads with mapping quality (mapQ) >20 were retained and PCR duplicates were removed. MapDamage v2.0.9[20] was used to evaluate the authenticity of the retained reads (Supplementary Fig. 1 and Data 1). The consensus genome sequences were generated using SAMtools mpileup (v1.8)[21] with parameter "--skip-indels" and BCFtools (v1.8)[22] with "consensus" command, in addition, each missing site was masked with letter "N" and all heterozygous loci were masked with the reference loci. As the ancient DNA was degraded, resulting in low-coverage genomes (*Megadyptes antipodes richdalei* 2.71-fold coverage, *Megadyptes antipodes waitaha* 0.05-fold coverage and 1.08-fold for *Eudyptes warhami*), we do not include these penguins in all the analyses (e.g. the positive selection gene analyses), and we only tentatively studied *Megadyptes antipodes richdalei* in the PSMC analysis.

Additional bird genomes for comparative genomics

To compare our penguin genomes to other bird genomes, we obtained the 361 bird genomes recently released by [23] as part of the B10K project (https://b10k.genomics.cn). These genomes represent 36 bird orders and 218 bird families.

Morphological data of modern and fossil penguins

Morphological data

We expanded the morphological dataset of [24], including a morphological tree and the morphological matrix by incorporating several additional fossil penguin species, including *Crossvalia waiparensis*, *Crossvallia unienwillia*, *Kupoupou stilwelli*, and *Kaiika maxwelli,* and seven additional morphological characters. The full matrix contains 72 fossil and extant penguin taxa, two outgroup taxa, and 281 morphological characters (Supplementary Data 5). We removed the two outgroups and used the same morphological patterns for the lineages from the same species in the downstream phylogenetic analyses.

Body size data of extant penguin taxa was obtained by average body mass of male and female "arrival at colony" values from [25] unless otherwise indicated (see Supplementary Data 3).

Sex identification

Penguin genomes were sexed by annotating chromodomain helicase DNA binding protein 1 gene (*CHD1*) (Supplementary Data 1). *CHD1Z* is present in both females and males while *CHD1W* is only present in females. Sequences of *CHD1* from *Gallus gallus* (Genbank accession number: NP_990272 [https://www.ncbi.nlm.nih.gov/protein/NP_990272.2/]) and *CHD1Z* and *CHD1W* from *Taeniopygia guttata* (Genbank numbers: NP_001071646 [https://www.ncbi.nlm.nih.gov/protein/NP_001071646], NP_001071647 [https://www.ncbi.nlm.nih.gov/protein/NP_001071647]) were downloaded from NCBI Refseq and mapped to our penguin genomes using tBLASTn (v2.2.26)[26]. Genewise (v2.4.1)[27] was used to annotate the coding regions for target hits. Protein sequences were then aligned to the NR database for annotation using the web version of BLAST available in NCBI.

Average sea surface temperature data

The average sea surface temperatures (calculated from a maximum of 20 spot locations obtained from near breeding colonies during both summer and winter seasons) was obtained for most lineages from https://earth.nullschool.net (Supplementary Data 3).

Generation times

Generation time of each extant penguin lineage was obtained from the IUCN, defined as the mean age of reproduction. For *Megadyptes antipodes richdalei* we use the same generation time as *Megadyptes antipodes antipodes* (Supplementary Data 2).

Phylogenomic inference

We combined all 27 penguin genomes comprising all extant and recently extinct species, subspecies and major lineages with a morphological matrix encompassing 74 extant and extinct penguins to resolve the timing and drivers of >60 million years of penguin evolution. In doing so, we update previous phylogenies (e.g.[1,6,28,29,30,31,32,33,34,35]) to include genomes and morphology from all taxa, including all major *Pygoscelis papua*

lineages and recently-extinct taxa. Previous studies have been limited in the sample coverage; e.g. only three studies have captured all extant penguin species (not including *Pygoscelis papua* lineages) in a single phylogeny ([34,35]: cytochrome oxidase subunit 1; [1]: mitogenomes; [6]: high-coverage genomes). Furthermore, while [31] used low-coverage resequenced penguin genomes, their dataset was incomplete, lacking *Eudypes robustus*, *Eudyptula minor*, *Eudyptula minor* "BAN" lineage and the extinct penguins *Eudyptes warhami*, *Megadyptes antipodes richdalei* and *M. a. waitaha*. In addition, while >50 fossil penguins have been described[36,37], only a handful of studies have combined morphological and molecular data under a single phylogenetic framework (e.g.[24,30,33]).

To explore the diversification of penguins using our comprehensive dataset, we undertook multiple phylogenomic analyses encompassing different subsets of taxa and inferred several phylogenomic trees (Fig. 1 and Supplementary Fig. 2). For some phylogenetic analyses we use *Thalassarche chlororhynchos*, *Oceanites oceanicus*, *Oceanodroma tethys*, *Taeniopygia guttata* and *Gallus gallus* as outgroups.

Genome alignments

We aligned our genomes using Cactus (v49e80082)[38]. All genomes were masked by RepeatMasker (v4.0.7)[39]. The guide tree for the penguin alignments was derived from [6] with four *Pygoscelis papua* penguins as a quadtree. Cactus was then performed with "AWS" and default parameters. The result of Cactus was merged to 363-bird alignments from the B10K project[23] using "halAppendSubtree" and the guide tree from the same project, with a bridge alignment aligned from the constructed most recent common ancestor (MRCA) of penguins and the constructed MRCA of Procellariiformes from Cactus. The final alignments were extracted by hal2maf, and multiple hits in the Cactus alignments were filtered out using the synteny-based pipeline previously used in the B10K project[23]. The alignment sequences were then used for downstream analyses.

We then created four alignments accounting for different subsets of taxa (based on taxonomic uncertainties discussed in [1,7]) to infer the phylogeny of penguins: 1) we included all putative species, subspecies and lineages (27 penguin taxa + 5 outgroups in total), obtaining a 574 Mbp length alignment with all extant species in each position; 2) we included all extant lineages (24 penguin taxa + 5 outgroups in total), removing the extinct *Megadyptes antipodes waitaha*, *M. a. richdalei* and *Eudyptes warhami* from the former alignment; 3) we extracted alignments to include all putative species and subspecies, removing the *Pygoscelis papua* "FAL", "KER" and "SG" lineages (21 penguin taxa + 5 outgroups in total), obtaining a 1.1 Gbp alignment with at least 24 lineages in each position; and 4) we included only putative species (19 penguin taxa in total), further removing the *Eudyptula minor* "BAN" lineage and *Eudyptes chrysolophus schlegeli* obtaining a 586 Mbp alignment with all species in position. In addition, we also created one large genome alignment for the evolutionary rate analysis with all 385-bird taxa (not including the extinct penguins *Megadyptes antipodes waitaha*, *M. a. richdalei* and *Eudyptes warhami*) in each position.

Initial phylogenomic analyses of modern penguins

To verify the phylogenomic relationships of modern penguins, we ran initial coalescent-based phylogenies using MP-EST (v2.0)[40] and ASTRAL-III[41], and concatenation-based phylogenies using ExaML (v3.0.21)[42], accounting for the different

subsets of taxa as described above.

We first inferred gene trees with genome alignments for coalescent-based methods. All genome alignments were split into 100 kbp non-overlapping windows for the downstream phylogenetic analyses while the first alignment was also split into 50 kbp non-overlapping genome windows. RAxML (v8.2.12)[43] was used to infer the best tree for each 100 kbp genome window from each alignment under the GTR+GAMMA substitution model following the parameters used in [6]. The highest-scoring maximum likelihood tree was inferred from 20 independent tree searches beginning from random starting tree topologies with 500 bootstrap replicates for each locus. In addition, IQtree (v1.6.12)[44] was used to infer trees using the GTR model and maximum likelihood with 1000 ultrafast bootstrap replicates for 11,485 50 kbp genome windows from the most complete alignment (32 taxa in total). Each gene tree was rooted with *Gallus gallus.*

MP-EST was conducted with rooted gene trees from RAxML. We performed three independent runs with different starting seeds to infer the species tree and bootstrap topology. Each run was completed with ten independent tree searches and the tree with the highest log likelihood score was retained. Following this, the highest-scoring tree amongst all three different runs was represented as the final tree for this strategy. ASTRAL-III was performed with gene trees inferred from multiple genome alignment datasets and methods (RAxML and IQtree) using default parameters. The concatenation-based method ExaML was also performed to infer the species tree following recent studies[6,45] with 21 full maximum likelihood tree searches and a GTR+GAMMA substitution model.

For species trees with coalescent units inferred from all coalescent methods, branch lengths were re-estimated in coalescent units of substitutions per site by constraining whole genome alignments to the tree topology using the "-f E" option in ExaML and bootstrap values were plotted using the " -f b -z bootstraps.nwk -t tree" option in RAxML.

The topology for all clades was strongly supported (bootstrap support: 100), and identical using all methods (Supplementary Fig. 1, Supplementary Data 5), except for the placement of *Eudyptes warhami* among *Eudyptes* lineages in the MP-EST phylogeny. The relationships of *Eudyptes warhami* and *Megadyptes antipodes richdalei* to other New Zealand endemic penguins show these lineages as sister to all other New Zealand species in their respective clades (*Eudyptes* and *Megadyptes*), contrasting [1], which found *Eudyptes warhami* to be sister to *E. sclareri*, and *Megadyptes antipodes richdalei* to be sister to *M. a. antipodes*. As the ASTRAL tree and ExaML tree have identical topology, we used the ASTRAL tree in the units of substitution per site as the representative phylogenetic tree.

Divergence time estimation using genome data

We used BEAST2[46,47] with a relaxed log normal clock model under the birth-death process to estimate the divergence time between modern taxa using four fossil node calibration points and three tip calibration points as outlined in [1] (Supplementary Data 5). We removed the fossil calibration *Pygoscelis calderensis* which constrained *Aptenodytes-Pygoscelis* in [1], as the placement of *Aptenodytes* as sister to all other extant penguins has since been revised[6,31]. We added one 'Crown Procellariiformes' (which is sister to penguins) calibration point to calibrate the divergence between the sampled

albatross and storm petrels (based on the oldest secure albatross fossil, *Tydea septentrionalis*), with a lognormal prior distribution mean of 11.8 (in real space), standard deviation of 1.0 and offset of 30 Ma. We also added three tip dates for three extinct taxa, using 1) the fossil *Madrynornis mirandus* to calibrate crown Spheniscidae, with a uniform prior distribution, a maximum age constraint of 25.2 Ma and a minimum age constraint of 9.7 Ma; 2) the fossil *Spheniscus muizoni* to calibrate both *Spheniscus* and *Eudyptula*, with a lognormal prior distribution mean 4.4 (in real space), standard deviation of 1.0, and offset of 9.2; and 3) the fossil specimen NMNZ S.046318 (*Eudyptes* sp.) to calibrate *Eudyptes-Megadyptes*, with a lognormal prior distribution mean 7.4 (in real space), standard deviation of 1.0, and offset of 3.06. In total, all 27 penguin lineages and five outgroups were used in this analysis . We ran BEAST2 three times with random initial trees and three alignments. Each alignment consisted of 50 k randomly selected single bases from the whole genome alignments (WGA). We conducted each run with 300 million Markov chain Monte Carlo (MCMC) generations and sampled every 200 generations. The stationarity and convergence (ESS values >200) of each run were assessed in Tracer (v1.7.1)[48] after discarding the first 30% of the MCMC chain as burn-in. Trees were then summarized using TreeAnnotator in BEAST2. All trees shared the same topology with our initial analyses, with the exception of the placement of *Megadyptes antipodes richdalei*, and had similar divergence times with each other (Supplementary Fig. 2b). *Megadyptes antipodes waitaha* had the same placement as in the mitochondrial genome phylogeny from [1].

Divergence time estimation of all penguins

We generated a Bayesian total-evidence dating tree using the fossilized birth-death process[49,50] in BEAST2 (Fig. 1), expanding[30] by including more species, genome data, and updating the morphology. We incorporate genomes from all modern penguins and morphology spanning crown and fossil penguins (Supplementary Data 5). All tip calibration points for fossils were shown in Supplementary Data 1.

Phylogenetic analyses were conducted for two subsets of taxa; 1) all 27 genomes and 47 fossil penguins; and 2) 25 genomes (excluding the *Pygoscelis papua* "KER", "SG", and "FAL" lineages and the *Eudyptula minor* "BAN" lineage) and 47 fossil penguins. Outgroup species were not added for this analysis as they could introduce biases for some of the priors[30,]. We used a random starting tree and accounted for uncertainty in the ages of each of the 47 fossils by sampling from uniform distributions bounded by the minimum and maximum ages for each taxon. We followed [30] model and prior selections for the fossilized birth-death process and log-normal relaxed clock. We additionally selected a Mkv model[51] with gamma-distributed rate heterogeneity for the morphological data. For the first subset of taxa, we performed three individual runs with three different 50 Kbp alignments without any constraint, and five individual runs with 50 Kbp alignments constraining most crown penguins (except for the genus *Aptenodytes*) as a monophyletic group. Each alignment consisted of 50 k randomly selected single bases from the WGA. For the second subset of taxa, we performed five runs with five different 50 Kbp alignments, constraining crown penguins as previously mentioned above. We conducted each run with 300 million MCMC generations and sampled every 200 generations. We determined that the chains converged on the same stationary distribution by visually assessing sampled parameters using Tracer. The first 30% of the MCMC samples were discarded as burn-in and the trees were summarized using TreeAnnotator (following [30]). Highest posterior distributions (HPD) values were extracted from the final tree using ape[52] and ggtree (v2.4.2)[53] in R v3.6.3

(Supplementary Data 1). The ape package was also used to plot the numbers of lineages through time (Supplementary Fig. 3a) and the $\delta^{18}O$ temperature scale was cited from [54].

For our total-evidence phylogenetic tree, when we did not constrain "*Eudyptes*, *Megadyptes*, *Spheniscus*, *Eudyptula*, *Pygoscelis*, *Inguza*, *Nucleornis* and *Madrynornis*" as a monophyletic group, we failed to recover *Aptenodytes* as sister to all extant penguin species instead *Spheniscus* was placed as sister to all extant penguins with high posterior probability. This result contrasts our previous phylogenetic analyses (Supplementary Fig. 2a) and the dated tree with constraints (Fig. 1), and recent phylogenetic studies on penguins (e.g.[31,6]). We suspect that this result is due to *Spheniscus* having a number of primitive morphological features that may draw it towards the root of the crown penguin tree, even though our molecular data clearly shows that *Spheniscus* is not sister to all other extant penguins (Supplementary Fig. 2a).

The relationships among all extant penguin taxa from all dating results have high posterior probabilities (>0.90) and are identical to the genome-based phylogenetic trees (Supplementary Fig. 2) after constraining the monophyletic group. The extinct *Megadyptes antipodes waitaha*, however, is sister to all other *Megadyptes*, with high support (Fig. 1), corroborating the topology of node-dating results (Supplementary Fig. 2b) and the mitochondrial phylogeny in [1]. The relationships of other extinct taxa (including both crown and stem penguins) with low posterior probability values do change in different runs. For example: *Madrynornis* may be sister to "*Spheniscus*, *Eudyptula* and *Inguza*" or "*Spheniscus*, *Eudyptula*, *Inguza*, *Eudyptes* and *Megadyptes*" and *Eudyptes atatu* may be sister to *Eudyptes* and *Megadyptes* or may belong to *Eudyptes* or *Megadyptes*. We compared the topologies of dated trees (Fig. 1) with former analyses to check the positions for the extinct taxa with well resolved taxonomic relationships in the crown (e.g. *Inguza*, which should be sister to *Eudyptula*). Though some small conflicts occur in our total-evidence tree (Fig. 1) compared with past parsimony results (e.g. [55]) or previously published total-evidence analyses (e.g.[30,34]), these relationships have low posterior probability values (Supplementary Data 5).

Pairwise genetic distance between penguin species

We calculated genetic distances between our 27 modern penguin genomes. To process such large genomic datasets, we calculated the number of transitions, transversions and equal sites for each two-taxa pair in 100 Kbp windows from the original WGA using a modified DiStats (v1.0) in parallel[56], and summarized the number of transitions, transversions and equal sites from all windows (Supplementary Data 1). The effective alignment length for each pair should equal the length of non-missing sites in the alignment, or the sum of the number of transitions, transversions and equal sites. K2P and P distances were then calculated using the summarised numbers based on their formulas.

Ancestral range estimation

We used BioGeoBEARS (v1.1.1)[57] in R v.3.6.3 to estimate the ancestral distribution of penguins. By including our comprehensive dataset, we expand on [24] and [31]. We used the total evidence dated phylogenomic tree and occupied areas following [24]. For the time stratification, we use six time slices (75, 40, 20, 2.6, 0.7 and 0.4 Ma) to account for different geographic areas being available for colonization, or changes in the tectonic positions of geographical areas through time. Distances were normalized against the shortest pairwise distance in the time slice, with the dispersal probability multiplied by

distance^X (+X models) for the distance matrix. We used ten geographical areas (A – J) outlined by [9] (Supplementary Fig. 3c); A) Australia (excluding Macquarie Island); B) New Zealand and nearby islands, including CI; C) Macquarie Island; D) Continental Antarctica and near-shore islands including within the Ross Sea; E) Antarctic Peninsula, South Orkney Islands, South Georgia, South Sandwich Islands and Bouvet Island; F) South America, Falkland Islands/Malvinas and the Galápagos archipelago; G) Gough Island. and Tristan da Cunha; H) Africa; I) Crozet Islands, Prince Edward and Marion Islands, Heard and Kerguelen Islands; J) St. Paul and Amsterdam Islands. We implemented 12 models: 1) dispersal-extinction cladogenesis (DEC); 2) dispersal vicariance analysis (DIVA-LIKE); 3) Bayesian inference of historical biogeography for discrete areas (BAYAREA-like) and their +J models (long-distance or "jump" dispersal, founder effect) and +X models (Dispersal probability a function of distance) (Appendix 1). We undertook standard model-testing (Likelihood Ratio Test and Akaike information criterion (AIC). The P-value of the Likelihood Ratio Test in Supplementary Data 1 demonstrated that we can reject the models without "+J". AIC and AICc model weights (Supplementary Data 1) show the relative probability of each pair. In the third pair, the DEC+J model could not be rejected. The DEC+J+X model was the best fitting model for our data (Fig. 1, Supplementary Fig. 3b and Supplementary Data 1). Maps were created and modified based on the map datasets from [58] and [59] to reveal the ancestral origin and subsequent dispersals of penguins across the Cenozoic using R with mapast[58], ggplot2 (v3.3.5)[60] and maproj (v1.2)[61] (Fig. 1 and Supplementary Fig. 4).

Incomplete fossil records may introduce biases in a biogeographical study. To account for the apparent dispersal/vicariance/etc events, we used a Biogeographical Stochastic Mapping (BSM) method. Using BSM the individual stochastic maps constitute possible histories, allowing stochastically mapped events to be counted. We performed BSM using our BioGeoBEARS results under the DEC+J+X model as input data to infer 1000 stochastic mapping trees and count the number for each event (Supplementary Fig. 3d).

Quantifying introgression and ILS between taxa

Controversy still remains regarding taxonomic boundaries between some closely related penguin taxa[1,62,63,64]: 1) *Eudyptes chrysolophus chrysolophus*/*E. C. schlegeli*[1,31,34,35,65,66]; 2) *E. chrysocome*/*E.filholi*/*E.moseleyi*[1,31,35,66,67,68,69,70], 3) *Megadyptes* penguins[1,2], 4) *Eudyptula* penguins[63,71,72] and 5) within *Pygoscelis papua* lineages[7,8,9]. Therefore, we undertook multiple analyses to assess 1) the discordance of gene trees; 2) the most recent timing of hybridization between taxa; 3) levels of incomplete lineage sorting (ILS); 4) introgression between taxa; and 5) the direction of gene flow between taxa, focusing on the five contested species groups (Supplementary Data 6).

Gene tree discordance was detected in the penguin phylogenetic analysis using Discovista (v1.0)[73] and Densitree[74] between the gene trees inferred from RAxML or IQtree and the species tree. Gene tree discordance results indicate the possibility of ILS and/or introgression events during penguin evolution. We therefore quantified the patterns of introgression and ILS between species using the tree-based method QuIBL[75,76,77]. To provide more evidence on introgression patterns, we further used DFOIL[78] and ABBA-BABA to determine the introgression related genome regions at the intra-genus or inter-genus levels that have also been determined by QuIBL. Finally,

we used hPSMC[79] to calculate the most recent timing of introgression events for some closely related lineages (subspecies included). Details can be found below.

Discordance of gene trees

Incongruences between penguin gene trees and species trees may be caused by ILS and/or introgression events. We therefore calculated the frequency of gene tree discordance for each internal branch using DiscoVista[73]. We used the Astral tree as the species tree and summarised the topologies for three different gene trees datasets using two input sets from the former phylogenetic construction; 1) 5743 gene trees inferred from the 100 Kbp windows of the first WGA using RAxML (Supplementary Fig. 5b); 2) 11,485 gene trees inferred from the 50 Kbp windows using IQtree (Supplementary Fig. 5c); and 3) gene trees were inferred by randomly selecting 5 kb in a 50 kb region through the WGA (windows with missing taxa were discarded) before reconstructing 10,615 trees with IQtree using the GTR model with 1000 bootstrap replicates (Supplementary Fig. 5d). We also plotted 500 randomly selected gene trees from RAxML trees and IQtree trees with the Densitree package[74] in R (Fig. 1c). The relative frequency results from three datasets indicated that discordances occurred at several internal branches (e.g. the most recent common ancestor of *Eudyptula minor*/*E. minor* "BAN" lineage or the *Pygoscelis papua* lineages; Supplementary Fig. 5).

ILS and introgression between penguins

We assessed levels of ILS and introgression by quantifying them via internal branch lengths using QuIBL (v1.0)[75] between all species. 100 runs of QuIBL were conducted and 500 trees were randomly selected from 10,615 trees inferred by IQtree using 5 Kb alignments to mitigate the linkage between loci for each run.

For each run, we ran QuIBL to calculate the non-ILS and ILS proportions and Bayesian information criterion values (BIC) under the "ILS-only" model and the "ILS + introgression" model for all triplets taken from the input trees. QuIBL examines and compares the internal branch length of a given triplet from a gene tree to the genome-wide distribution of branch length in that triplet under coalescent theory to identify if it is introgressed. The number of steps was set to 50 and *Gallus gallus* set as the outgroup for rooting all triplets in parameters. Triplets with the identical topologies as the FBD species tree were discarded from the following analyses. The two alternative topologies of each three species combinations were taken into account for discordance analysis. We then defined a preferred model for each of the three species combinations. If the absolute difference value of the delta BIC values under two models for one triplet is greater than 10, the model with the lower delta BIC value is a better fit for this triplet. If the absolute delta BIC is smaller than 10, the "ILS-only" model should be the preferred one. For visualization, we estimated the average proportions of ILS and introgression for each triplet based on the mixing proportions under the preferred model from all QuIBL results (Supplementary Data 2). We then calculated the average ratios for each two taxon pairs. Trees were plotted using ggtree packages in R v. 3.5.2 after removing the average ratios below 0.05% (Supplementary Fig. 7 and Supplementary Data 2). We used a parsimony principle described in former analyses[76,77] to summarize the possible ILS and introgression events (Fig. 2a). If ILS/introgression events were detected between all lineages from clade A and all lineages from clade B, then we assume that there is an ILS event among the ancestral lineages of clade A and B, or an introgression event between the ancestral lineages (Supplementary Data 6).

We also used DFOIL (v1.0)[78] to test the direction of introgression among lineages and to assess what genomic regions have introgressed. We analysed 16 five-species combinations with symmetric phylogenies (Supplementary Fig. 6a; Supplementary Data 2). The extinct penguins *Megadyptes antipodes waitaha*, *M. a. richdalei* and *Eudyptes warhami* were removed from this analysis due to their low genome coverage. We divided the raw whole genome alignment of all penguins and one outgroup taxon, *Thalassarche chlororhynchos*, into 100 Kbp non-overlapping windows, with the recommended length of input data, and excluded the windows if five or more taxa have more than 50% gaps in the windows. We then performed DFOIL with the "dfoil" model for each window for each combination and summarized the results using dfoil_analyze.py, available in DFOIL (Supplementary Fig. 6a; Supplementary Data 2). Significance for each window was then tested using a chi-square test. Introgressed windows with numbers more than 10 were retained.

We also conducted an ABBA-BABA test using DFOIL with the "dstat" mode for 17 four-taxon combinations with default parameters (Supplementary Data 2 and 6). Among 17 combinations, we selected different taxa from different genera to examine possible introgression between different genera, as well as some closely related lineages/species that could not be tested by the DFOIL five-taxon test, to examine whether gene flow occured between them. We extracted the 100 Kbp non-overlapping genome windows for each group for the ABBA-BABA test (Supplementary Fig. 6b). We also tried to recover the introgression events in the last common ancestor of taxa if the descendant lineages shared similar probabilities (Supplementary Data 2).

Timing the cessation of introgression events

We used hPSMC (v1.0)[79] to assess the cessation of gene flow between six closely related penguin groups: 1) *Eudyptula* lineages; 2) *Pygoscelis papua* lineages; 3) *Eudyptes chrysolophus chrysolophus*/*E. c. schlegeli*; 4) *Eudyptes robustus*/*E. pachyrhynchus*; 5) *E. chrysocome*/*E. filholi*/*E. moseleyi*; 6) *Spheniscus* penguins. We did not analyze *Megadyptes* because the coverage for the ancient genomes was too low.

For each species pair from the 20 hPSMC groups, hPSMC was run by mapping the clean reads from one penguin genome to the reference genome of the other penguin genome using Sentieon BWA-mem (v0.2.0)[80] or BWA-mem based on our available computing resource with default parameters. We generated the haploid consensus sequences using SAMtools mpileup (v.1.8) and Pu2fa, with a minimal mapping quality of 30 and a minimal base quality of 30. We then combined the concatenated haploid fasta and the reference genome into a pseudo-diploid sequence file using the psmcfa_from_2_fastas.py in hPSMC toolsuite with a bin value of 10. We also used different genome references for two pairs in this step to determine the possible biases from references. We ran PSMC using the pseudo-diploid sequence file as input data and visualized this using an average mutation rate per generation time and average generation time of each species pair. The pre-divergence population size (N_e) for each pair was estimated visually based on the plot. We then ran simulations using the estimated pre-divergence N_e to infer the confidence intervals with hPSMC_quantify_split_time.py available in the hPSMC tool suite, specifying the time windows based on the divergence times from the dated tree (Fig. 1). After checking convergence, the divergence time range was estimated based on the narrowest range of

non-overlapping simulations surrounding the intersectional simulations between 1.5x and 10x of the pre-divergence N_e in the plot (Supplementary Data 2). The demographic history of the pseudodiploid genomes derived from different mapping reference genomes is nearly identical (Supplementary Figs. 8-9, Supplementary Data 2 and 6).

As different methods have different limitations, e.g. DFOIL can only be used for five-species combinations with symmetric phylogenies while ABBA-BABA can only be used for four-taxon combinations, and they cannot be used to detect the ILS proportions between taxa. We therefore used the QuIBL results for the discussion between species with the support and supplementation of the DFOIL and ABBA-BABA introgression test results. We also used the hPSMC results for the discussion between closely related lineages for the timing of their cessation of introgression events.

Demographic history of penguins

As penguins are sensitive indicators of climate and environmental change[81,82,83,84,85,86,87,88,89,90,91,92,93] and are the most threatened group of seabirds after Procellariiformes[94], we undertook analyses of demographic history. We used two methods to explore demography in penguins: 1) profiling heterozygosity across each genome, and 2) undertaking Pairwise Sequentially Markovian Coalescent (PSMC)[95] analyses of effective population size (N_e) over the last 1 Ma.

Profiling heterozygosity across the genome

We used 100 kb sliding windows to calculate heterozygosity for penguin genomes and for avian genomes from [23]. After removing gaps, heterozygosity was calculated as heterozygous sites per genome size (Supplementary Data 2). For avian genomes, heterozygous sites were calculated by filtering the SNPs distance below 10 bp, filtering the SNPs depth below 1/3 mean depth or over 2x mean depth and filtering the SNPs root-mean-square mapping quality lower than 25 (Supplementary Data 2). One sided T-tests or Wilcoxon-tests were performed for heterozygosity rate comparison for penguins and other avian orders after the tests for normal distribution (Shapiro-Wilk's test) and homogeneity (Bartlett's test) in R (Supplementary Data 2). Heterozygosity of penguins was lower than the combined heterozygosity in 344 other birds (one sided Wilcoxon Rank sum test, P-value <0.0001), but penguins do not show a significant low level for heterozygosity rates at bird order levels (Supplementary Fig. 10 and Table 2).

Demographic responses to climate change

We undertook PSMC analyses of N_e over the last 1 Ma. We used Picard (v1.115) for manipulating each species BAM file, which was generated by BWA. Then we called SNPs for all extant penguin genomes (Supplementary Data 2 and Data 4) using HaplotypeCaller implemented in GATK (v4.0.5.2)[96]. There were very few SNPs for *Megadyptes antipodes antipodes* (197,533) and *Spheniscus mendiculus* (250,589). For the *Eudyptes warhami*, *Megadyptes antipodes richdalei* and *Megadyptes antipodes waitaha*, we followed [97] and retained transitions. We extracted reads from the BAM file, and trimmed 5 bp off the read's head and tail using SOAPnuke (v1.5.6)[98]. We used BWA to map the reads against *Eudyptes sclateri* (for *E. warhami*), or *Megadyptes antipodes antipodes* (for *M. a. richdalei* and *M. a. waitaha*). We called SNPs using SAMtools (v1.4.1) with a minimum base quality equal to 30 and a minimum mapping quality equal to 37 (--min-BQ 30 --min-MQ 37). We only retained 798 heterozygous sites for *M. a. waitaha*, 25,8422 for *Eudyptes warhami* and 1,063,459 for *Megadyptes*

antipodes richdalei. We then retained heterozygous sites of depth ≥10 and removed transitions. We were only able to retain 38 heterozygous sites for *M. a. waitaha*, 887 heterozygous sites for *Eudyptes warhami* and 6717 heterozygous sites for *Megadyptes antipodes richdalei*. We then set different depths (≥3-10) to retain heterozygous sites. Using a depth ≥3, we retained 798 heterozygous sites for *M. a. waitaha*, 258,422 heterozygous sites for *Eudyptes warhami* and 1,063,459 heterozygous sites for *Megadyptes antipodes richdalei*. As the number of heterozygous sites for *M. a. waitaha* and *Eudyptes warhami* remained too low for PSMC, we only present PSMC for *Megadyptes antipodes richdalei*. However, we are aware that the amount of data retained (1,063,459 SNPs) is low, but given we undertook 5bp trimming and BaseQ30 & MapQ37, we believe our results for *M. a. richdalei* remain useful for our study. We used the species divergence time tree as an estimation of the mutation rate in PSMC and detail the divergence times in Supplementary Data 2. We ran PSMC focusing on the last 500 Kya, by setting -p to 4+100*1+4+6+10, and the other parameters are -N30 -t15 -r5. This period encompasses dramatic glacial/interglacial cycles (Appendix 2). As PSMC tends to generate biased estimates for more recent population histories[95], we do not focus our discussion on the Last Glacial Maximum (18 - 25 Kya). For species that diverged <3 Ma, N_e prior to the divergence represents the population size of the common ancestor. Generation times were obtained from the IUCN, defined as the mean age of reproduction (Supplementary Data 2). For *M. a. richdalei* we use the same generation time as *M. a. antipodes*. However, based on contrasting generation times used in previous studies (e.g.[31,99,100]), and because our generation times are calculated on limited/non-existent field data (e.g. for *Eudyptes sclateri* and *E. warhami*) we assume our generation times may have an error of approximately 40%. We therefore re-calculated PSMC based on upper and lower generation bounds within the error margin (Supplementary Fig. 11a). To investigate patterns among our data during the Last Glacial Period, we converted N_e for each taxa to a percentage of the maximum N_e (Supplementary Fig. 11b). Our results suggest that penguins were impacted by ecosystem-wide, climate-driven refugia/recolonization cycles in the Southern Ocean[87,99,100], a pattern also observed in other marine taxa, including southern elephant seals[101] and kelp[102] during the Last Glacial Maximum.

Comparison of evolutionary rate

Evolutionary rate among bird orders

Previous studies have proposed that temperature, water availability, population size, and spatial heterogeneity may influence evolutionary rate[103]. Life history traits also impact evolutionary rate[104,105], but such relationships remain incompletely understood in birds[106]. Penguins are long-lived, large-bodied and produce few offspring, thus providing an ideal case study in how life history may impact evolutionary rate[107]. The evolutionary rate between penguins and other birds was compared using several methods (Supplementary Fig. 12 and Supplementary Software). First, we manually merged the species tree of 363 bird taxa from [23], provided by the B10K consortium, and the species tree of extant penguins inferred from this study. We then constructed the whole genome alignment for all extant penguins plus the 361 bird taxa (as discussed in the genome alignment section, resulting in a 19 Mb non-missing alignment). Sequences for the recent common ancestor of all 385 taxa and the latest common ancestor of all Neoaves were also constructed using Cactus.

P and K2P distance comparison was calculated using the p and k2p distances between birds and their ancestors (Supplementary Fig. 12; Supplementary Data 3), and compared among bird orders using the Shapiro test and one-sided Wilcoxon Rank sum test (Supplementary Fig. 13). The "Tip to root" rate comparison method was undertaken using branch lengths for the species tree, which were re-estimated in units of substitution per site by constraining the whole genome alignment to the tree topology using ExaML v.3.0.21, using the option -f E. The "tip to root" rate was then calculated with the substitution per site, extracted from the inferred phylogenomic tree using the R packages ape (v5.5) and RRphylo (v2.5.8)[108], and the divergence time was extracted for each bird order with at least three taxa from the published dated tree[109]. The substitution per site and rates were then compared with the Shapiro test and one-sided Wilcoxon test, respectively (Fig. 3a, Supplementary Fig. 13 and Table 3). Orders with conflict topologies were then removed from the analysis.

Evolutionary rate of penguins

We calculated the evolutionary rate of penguins using the following formula: substitution rate (substitution per site per year) = substitution per site / divergence time. We extracted the substitution per site from the ASTRAL-ExaML tree (Supplementary Fig. 2b) using the R package ape and ggtree for each lineage, and then extracted the divergence time from the dated tree (Fig. 1). The correlation relationship between the substitution rate and sea surface temperature for extant penguins was also tested using a phylogenetic generalized least squares (PGLS) regression with R phylolm (v2.6.2)[110] to reduce the effects of the phylogeny (Fig. 3; Supplementary Data 3). To test the Evolutionary Speed Theory[111], we also conducted PGLS regression analysis to determine the correlation relationship between sea surface temperature and body mass or generation time. Model selection was conducted using the BIC test (Supplementary Data 3). We also calculated the rate for each branch inside the tree and regarded it as the rate of the common ancestor of penguins. The trend line of the evolutionary rate for extant penguins and the ancestors of extant penguins was then plotted using R ggplot2 with "geom_smooth(method = loess)" (Fig. 3b; Supplementary Data 3).

Genome size and repeat elements

We compared the genome size among birds to check whether the genome size has a correlation with the proportion of repeat elements (Supplementary Data 3). Firstly, we downloaded the data of repeat elements for 361 other birds from [23]. We then conducted repeat annotation analysis for penguins using the repeat annotation process used in [23]. We identified tandem repeats using Tandem Repeats Finder (v4.07b)[112] and transposable elements using RepeatMasker (v4.0.7) and RepeatModeler v1-0-8[113] for each penguin genome. All repeat elements were then classified into seven categories (DNA, long interspersed nuclear element [LINE], short interspersed nuclear element [SINE], long terminal repeat [LTR], other, unknown, tandem repeat) according to classification in repeat databases. Finally, we measured the linear relationship between genome size and the percentage of repeat elements using a linear regression with R lm function and a PGLS regression with R phylolm and the best-fitting model "OUrandomRoot": the Ornstein-Uhlenbeck model with the ancestral state at the root having the stationary distribution (Supplementary Data 3).

Putative molecular adaptations

We undertook comparative genomic analyses across all extant penguin taxa to identify

genes and regulatory changes contributing to the remarkable morphological and physiological variation within penguins[37,114,115]. We do not include *Eudyptes warhami*, *Megadyptes antipodes richdalei* or *M. a. waitaha*, or additional *Pygoscelis papua* lineages (Falkland/Malvinas, South Georgia, Kerguelen) in these analyses. Our analyses expand on previous analyses that have only examined *Aptenodytes forsteri* and *Pygoscelis adeliae* (e.g.[5,116]), or those that have relied only on a site analysis for penguins (e.g.[31]).

Comparing penguins to other birds

To understand the adaptive evolution of specific phenotypes in the branch leading to the last common ancestor of penguins, we identified positively selected genes, rapidly evolving genes and evolutionarily conserved genes for extant penguins under a branch model and a branch-site model using PAML v4.9[117,118].

The branch model is used to detect if a given gene evolves faster on the target branch. The branch model considers that the omega values for all sites in the gene are the same, and compares two models; 1) a null model, where all branches have one omega value; and 2) a model where the target branch has one omega value, and all other branches have another omega value. Using this model, we compare the likelihood of the two models. We also calculated the P-value using the chi-square test (where the degree of freedom is 1) and P-values were adjusted. If the q-value is ≤0.05, and the omega value on the target branch is higher than the background value, the gene is considered to be a rapidly evolving gene. If the omega value on the target branch is >1, it is considered to be a positive selected gene.

The branch site model is used to detect whether a gene has a positive selection site on a certain clade. This model considers that the foreground branch has one omega value, and all other branches have another omega value. This model compares the two models; 1) the first model is model=2 NSsites=2, which divides the omega values into three categories (<1, =1 and >1); and 2) the second model is consistent with the former, except that omega is fixed to 1 as a null model. We calculated the posterior probability of a positive selection site by Bayes Empirical Bayes (BEB) method. We compared the likelihood of the two models, and used the chi-square test to calculate the P-value, and P-values were adjusted. If the q value is ≤0.05 and there exists reliable positive selection sites (the upstream and downstream 5 amino acids were perfectly matched), then it is considered to be a positive selected gene.

To do this, we obtained orthologous genes against the *Gallus gallus* genome using Cactus for 44 species: all penguins (excluding *Eudyptes warhami*, *Megadyptes antipodes richdalei*, *M. a. waitaha* and additional *Pygoscelis papua* lineages ['FAL', 'KER' and 'SG']), *Thalassarche chlororhynchos*, *Oceanites oceanicus*, *Fregetta grallaria*, *Hydrobates tethys*, *Pelecanoides urinatrix*, *Calonectris borealis*, *Fulmarus glacialis*, *Urile pelagicus*, *Phalacrocorax carbo*, *Nannopterum harrisi*, *Nannopterum auritus*, *Nannopterum brasilianus*, *Rhynochetos jubatus*, *Eurypyga helias*, *Zapornia atra*, *Atlantisia rogersi*, *Heliornis fulica*, *Psophia crepitans*, *Grus americana*, *Aramus guarauna*, *Chlamydotis macqueenii*, *Ardeotis kori* and *Lophotis ruficrista*. We retained a total of 8716 high-confidence orthologous genes. These genes were then used to conduct a multiple sequence alignment using PRANK (v170427)[119] and GUIDANCE (v2.02)[120]. We then performed the codeml algorithm for the branch and the branch-site

models implemented in PAML by testing two different branches; 1) we detected positively selected genes/rapidly evolving genes in the branch leading to the last common ancestor of penguins; and 2) we detected positively selected genes/rapidly evolving genes in the branches of the last common ancestor of penguins plus four flightless/nearly flightless birds (*Nannopterum harrisi*, *Rhynochetos jubatus*, *Zapornia atra* and *Laterallus rogersi*). The likelihood ratio test of hypotheses through a comparison of implemented models to calculate the P-value was undertaken in order to detect positive selection at amino acid sites or genes. The P-values of genes were then corrected using the Benjamini-Hochberg adjustment. Finally, genes with a false discovery rate adjusted P-value <0.05 were treated as candidates for positive selection or rapid evolution (Supplementary Data 4).

To reveal more characteristics in penguins, we also used PROVEAN (v1.1.5)[121] to predict whether an amino acid substitution site may have an impact on the biological function of a protein, by comparing penguins to the 23 other birds, and scanning for premature stop codons in each gene alignment.

We also examined some specific genes individually, such as hemoglobins, gustatory genes, opsins and transient receptor potential (*TRP*) genes, with well characterised functions (e.g. gene regulatory pathways related to light transmission[122]). We used the *Gallus gallus* protein coding sequence to re-annotate these genes in penguins and other birds using BLAST and genewise software, and then followed the same steps as above for analysis.

In addition, we checked whether the positively selected genes that were found by [31] were positively selected in our dataset, following the same steps as outlined above for the branch, branch-site and pseudogene analysis.

In the branch leading to the last common ancestor of penguins, 28 positively selected genes (false discovery rate [FDR] q <0.05) and 13 rapidly evolving genes (FDR q <0.05) were detected. In the branch leading to the last common ancestor of penguins and the four flightless/nearly flightless birds, five positively selected genes (FDR q <0.05) and 38 rapidly evolving genes (FDR q <0.05) were detected.

Oceanic diving

We identified three rapidly evolving genes that are shared by penguins and other flightless/nearly flightless birds. These are likely associated with the shortening, rigidity and increased density of the forelimb bones. *TBXT* and *FOXP1* are related to development of articular cartilage, tendons and limb bones[123,124,125,126,127,128,129,130]. *SMAD3* is involved in the transforming growth factor beta signaling pathway, which is important for maintaining articular cartilage and stimulating osteogenesis and bone formation[131]. *TNMD*, a positively selected gene, is expressed during the differentiation and developmental phase of limb tendon, ligament and collagen fibrils, and loss of *TNMD* can result in reduced tenocyte density[132,133].

Calcium is important for high bone density. We annotated genes related to calcium sequestration, comparing penguins, *Podiceps cristatus* and *Podilymbus podiceps*, species that have high bone density, to other birds. We also used OrthoMCL (v1.4)[134] to identify ortholog groups in penguins and other birds. Compared to other birds,

penguins, *Podiceps cristatus* and *Podilymbus podiceps* tend to exhibit more gene duplication of the family "SLO"-type large-conductance channels (*KCNMA1, KCNT1, KCNT2, KCNU1*), which play important roles in calcium-activated potassium channels and increase in intracellular calcium ion concentrations, and we inferred a phylogenetic tree for these genes using PhyML (v3.0)[135] (Supplementary Fig. 15). These genes may contribute to high bone density characteristic of these taxa, which helps reduce buoyancy for deep diving[136].

Thermoregulation

We identified four genes under selective pressure in common ancestors of penguins that are related to thermoregulation. These genes (*APPL1*, *TRPC1*, *EVPL*) showed evidence of positive selection or rapid rates of evolution on the branch leading to the last common ancestor of extant penguins but not in other birds (Fig. 4a). The white adipose tissue of penguins is important for survival in the cold, acting as an insulative layer and an energy reserve, particularly ahead of catastrophic moult[137]. Several of these genes may contribute to white adipose fat storage. *APPL1* and *TRPC1* are related to glucose levels and fatty acid breakdown through adiponectin[138,139].

Oxygenation

Among aquatic birds, penguins are the most specialised and adapted for prolonged diving[140,136], and function under hypoxic conditions in part via myoglobin concentration and utilizing anaerobic metabolism[140,141,142]. Of particular interest are the oxygen-binding globins, hemoglobin and myoglobin[141,143,144]. We retrieved sequences of the hemoglobin genes in 44 avian genomes using the *Gallus gallus* hemoglobin sequences as queries (*HBA*-αA, *HBA*-αB, *HBA*-π, *HBB*-βA, *HBB*-βB, *HBB*-ε, *HBB*-ϱ) (Supplementary Fig. 18). As the myoglobin gene was a positively selected gene (P-value: 0.0135) in the node of the penguin ancestor, we selected the sites with a posterior probability >0.99 by using the BEB method for calculating posterior probabilities for site classes that are implemented for the branch-site model (Fig. 4d). We obtained the *Gallus gallus* myoglobin protein and hemoglobin protein to predict protein 3D structure by changing these sites with the penguin-specific substitutions. We used the latest protein structure prediction software AlphaFold2[145] and 5 models which were used during CASP14 to predict the structure of two myoglobin proteins respectively. The per-residue local distance difference test (pLDDT) scores of both proteins reached 96, indicating that the prediction accuracy with the high confidence. While hemoglobin is made up of four polypeptide subunits, Alphafold2 cannot predict multi-chain structure. Therefore, we used the structural bioinformatics web server SWISS-MODEL (v2021.5)[146] to predict the protein structure of hemoglobin proteins. *Gallus gallus Hb* and penguin *Hb* had QMEAN values of 0.81 and 0.80, respectively. The sequence logos were created by Weblogo[147]. The different molecular models showed that the specific amino acid substitutions may be responsible for the increased *Mb*-O2 and *Hb*-O2 affinity in penguins (Supplementary Data 7). We identified seven genes related to oxygenation that are under positive selection or have penguin-specific substitutions in penguins. The Transferrin Receptor 1 hypoxia-regulated gene (*TFRC)* shows positive selection in penguins[148]. Previous experimental work in cells has reported that *TFRC* messenger RNA is expressed in an oxygen-dependent manner[149]. *TFRC* is a top candidate gene for the hypoxia response of domesticated cattle[150]. While none of the hemoglobin genes were positively selected genes (P-value: >0.05), we observed that HBA-αA (A140S) and HBB-βA (L87M) genes (Fig. 4c and Supplementary Fig. 18)

show penguin-specific amino acid substitutions that are highly conserved across all penguin species. *MB* is an oxygen-binding myoglobin gene that shows positive selection at multiple sites both between penguins and other birds and among penguins (Fig. 4d). These penguin-specific substitutions may impact the stability of the resulting myoglobins, as seen in extreme deep-diving (>1000 m) cetaceans[144,151]. Another positively selected gene, *TRPC4*, is involved in the cardiovascular system[152].

Underwater vision
Penguins frequently forage in low light, and exhibit specializations for vision in dim, blue-green marine environments[153,154,155,156]. We retrieved sequences of the *TRP* channel superfamily, which are known to be involved in sensations including pain, temperature, taste, pressure and vision, in our penguin genomes and 11 other avian genomes: *Gallus gallus*, *Manacus manacus*, *Geospiza fortis*, *Opisthocomus hoazin*, *Balearica regulorum*, *Alectura lathami*, *Rhea americana*, *Struthio camelus*, *Mesitornis unicolor* and *Columbina picui*. We then annotated 26 *TRP* genes in those birds, and used PAML to test for positively selected genes along the penguin ancestral lineage. Five rapidly evolving *TRP* genes (*TRPC1*, *TRPC4*, *TRPML1*, *TRPM5* and *TRPM1*) and one positively selected *TRP* gene (*TRPC4*: P-value <0.05) were detected in penguins. In addition, two scotopic photoresponse genes, *TMEM30A* and *KCNV2*, show evidence of selection in penguins, and two others, *CNGB1* and *GNB3*, each have a site mutation unique to penguins (Fig. 4a and Supplementary Fig. 19). These genes play an important role in the transmission of light (Fig. 4b), and may further enhance visual sensitivity at low light levels, as mutations or loss of these genes impact the result in a reduced scotopic photoresponse[157,158]. PSGs were then annotated by KOBAS (Supplementary Data 4).

Comparing specific penguin taxa to other penguins
We also undertook branch and branch-site analyses to compare specific penguin taxa/genera to other penguin taxa/genera, based on interesting morphological and physiological characteristics. To do this, we only selected two petrels as outgroups (*Oceanites oceanicus* and *Hydrobates tethys*), and we retained a total of 10,805 orthologous protein-coding genes. We used the branch and branch-site models to identify positively selected genes and rapidly evolving genes in the target branches (Supplementary Data 4). For example, we tested the branch leading to the common ancestor of *Aptenodytes* which include the two penguins with the largest body size, and those that are the deepest divers; 2) the branches of *Spheniscus humboldti* and *S. mendiculus* that inhabit the warmest climates; 3) the branch leading to the common ancestor of *Eudyptes*, as those penguins all have a unique yellow crest; and 4) the branch leading to the ancestor of *Eudyptes*/*Aptenodytes*/*Megadyptes*, as all those genera have plumage colored with a yellow pigment that is known only from these penguins[159]. We were unable to relate any of the genes found in the crest, yellow pigment or warm adaptation analyses (Supplementary Data 4) to those adaptations.

Large body size in Aptenodytes
In the bLCA of *Aptenodytes,* 25 PSGs (FDR q <0.05) and 3 REGs (FDR q <0.05) were detected. We found two genes associated with large body size that are under positive selection in *Aptenodytes* compared to all other penguin lineages (Fig. 4a). *CREB3L1* is important during bone development, and vertebrates lacking *CREB3L1* have underdeveloped growth[160]. *SMARCAD1* is related to the skeleton and plays a role in

transcriptional regulation, maintenance of chromosome stability and various aspects of DNA repair. Vertebrates with mutant *SMARCAD1* also have underdeveloped growth[161].

Comparisons among penguin species

In order to detect positive selection within the penguin lineage, without *a priori* predictions about the branches along which selection occurred, the site models in PAML were employed. The alignments used for the branch-site analyses were pruned to leave only penguin sequences, and were then realigned using a codon alignment in PRANK. Next, PAML site models M1a (Nearly Neutral) and M2a (Positive Selection) were fitted to the alignments. Likelihood ratio tests were performed on pairs of log-likelihood values and P-values obtained from the χ^2 distribution with df = 2. To control for multiple hypothesis testing, q-values were computed based on the distribution of P-values using the qvalue package in R (v. 4.0.2), and a q-value threshold of q <0.01 was considered significant. Alignments for all genes identified as positively selected were manually checked to confirm the absence of alignment error, assembly error or non-codon-based variation that could falsely be interpreted as positive selection. Identities of sites under selection were obtained using the BEB procedure and were considered significant at posterior probability >0.9.

Within penguins, 275 positively selected genes (FDR q <0.01) were detected (Supplementary Data 4).

Oxygenation

Of the positively selected genes, *FIBB* and *ANO6,* which are involved in blood coagulation[162,163], showed a signal of positive selection in *Aptenodytes*, but not other genera (Supplementary Fig. 17). Among all penguins, *Aptenodytes* have the capacity for the deepest diving (>500 m depth), 22 min duration[143], and gene variants may therefore enable these species to dive to extreme depths.

Host-pathogen interactions

We detected 51 positively selected genes in penguins that have a role in immunity (Supplementary Data 4). Several of these genes might be under positive selection corresponding to host-pathogen co-evolution. For instance, we confirm previous reports[164] that the bacterial-recognizing Toll-like receptors *TLR4* and *TLR5* (Fig. 4a and 5b) are positively selected in penguins[164]. Moreover, the positively selected sites located proximal (<5Å) to the lipopolysaccharide binding site in *TLR4* (codon 276, homologous to chicken codon 302[165]) and at a flagellin-binding site in *TLR5* (codon 33[166]) (Fig. 5b), are both in domains crucial for bacterial recognition. We detected several other pattern-recognition receptors, such as *IFIT5*, that are also under positive selection in penguins (Fig. 4a). *IFIT5* is a cellular detector of viral RNA[167], and we found a cluster of positively selected sites located in a connecting helix forming part of the RNA-binding cleft (codons 407, 409, 413 and 421, corresponding to human codons 412, 414, 418 and 426[168,169]) (Fig. 5b). We also found evidence of positive selection at viral targets of cell entry. For example, *CD81* is a co-receptor required for glycoprotein-mediated hepatitis C viral entry into cells in mammals[170], and positive selection has been reported at the glycoprotein interface in bat *CD81*[171]. We also found a cluster of positively selected sites in the hepatitis C glycoprotein interface in penguin *CD81* (sites 181, 182 and 186, corresponding to human sites 180, 181 and 185, and penguin site 86, corresponding to human site 185) (Fig. 5b). Finally, we detected positive selection in penguin transferrin,

which is part of the "nutritional" immune system that sequesters iron from iron-scavenging pathogens[172]. Outbreaks of diphtheritic stomatitis in *Megadyptes antipodes antipodes* has caused increasing chick mortality, and is hypothesised to be related to an increasing susceptibility to corynebacterium as a secondary infection[173] potentially triggered by chick malnutrition due to changes in diet[174], and potentially iron intake. The co-evolutionary arms race to sequester and scavenge iron has also been detected in mammals[175] and fishes[176,177].

Pseudogene analyses
We built upon analyses of *Pygoscelis adeliae* and *Aptenodytes forsteri*[5], and a retinal micro-spectrophotometric study in *Spheniscus humboldti*[153]. We ran a discontiguous megablast with opsin gene reference sequences encompassing exons, introns and flanking regions against our penguin assemblies in NCBI's Whole Genome Shotgun (WGS) collection. We aligned all penguin sequences against their respective reference sequence using MUSCLE v. 3.5[178] in Geneious v. 2019.2.3[179]. For several genes (e.g. *LWS*, *SWS1*, *SWS2*), the assemblies were of poor quality, as demonstrated for previous genome assemblies[180], so we mapped the short sequencing reads against individual exons + flanking sequence in Geneious to fill gaps in the assemblies. We then examined all gene sequences for traditional inactivating mutations: start codon mutations, premature stop codons, frameshift indels and splice site mutations. Furthermore, we searched for disabling mutations shared by more than one taxon, given that these provide evidence of pseudogenization in a common ancestor.

Underwater vision
Morphological research has shown that at least some penguins are cone trichromats with only three functional cone photoreceptor types, have blue-shifted long-wavelength visual pigments and lack red oil droplets[153]. Genomic data support trichromatism in all penguins. The inactivation of the green cone opsin gene (*RH2*) in the stem penguin lineage is inferred by a 12 base pair (bp) deletion, which encompasses the codon for the critical chromophore-binding lysine (K296; [181]) (Fig. 4a and Supplementary Fig. 19a). As all penguins share this deletion, reduced color vision must have occurred in the penguin stem lineage, similar to secondarily aquatic mammals[182]. In addition to lacking green cones, the functional orthologs of the remaining visual opsins in penguins strongly indicate the retention of violet (*SWS1*), blue (*SWS2*) and red (*LWS*) cones, plus rods (*RH1*) (Fig. 5a). Furthermore, the peak wavelength sensitivity (λ_{max}) of penguin *LWS* opsins show evidence of shifts in spectral sensitivity to better match ambient underwater light. Penguins possess substitutions at five key tuning sites in *LWS*, four of which (A180, F277, A285, and S308) are associated with blue-shifting this pigment[183]. This suggests that this opsin has been fine-tuned for marine foraging, as observed in cetaceans[182]. *CYP2J19*, which encodes a carotenoid ketolase responsible for producing red oil droplets in avian cones[184,185], has also been inactivated in most penguins.

Taste and diet
Gustation has been well-characterized in vertebrates[186], with taste modalities existing for sweet, umami, sour, salty and bitter perception. In addition, a study on taste in five penguins[116] found evidence that the umami receptor has been lost in this clade. We targeted the following genes pertaining to gustation: *TAS1R1*, *TAS1R2*, *TAS1R3*, *TAS2Rs*, *PKD2L1*, *SCN1A*, *SCNN1B* and *SCNN1G*. We used chicken protein sequences

as a reference for genes *TAS1R2, TAS2Rs, PKD2L1*, *SCN1A*, *SCNN1B* and *SCNN1G*, and re-annotated these genes in all penguins and outgroups using Genewise. We expanded on [116] by analysing umami taste receptor genes (*TAS1R1* and *TAS1R3*) in all penguins, and ran a discontiguous megablast with reference sequences encompassing exons, introns and flanking regions against our assemblies in NCBI's WGS collection and proceeded as described for opsin genes above (but without short-read mapping step). We verified that penguins only retain genes associated with detecting sour and salty tastants, and lack functional copies of genes linked to umami, sweet and bitter tastants[187] (Figs. 4a and 5a).

A strong genomic indicator of diet is presented by chitinases expressed in the gastrointestinal tract. These chitinases belong to several paralogs of acidic mammalian chitinase genes (*CHIA*s), and vary such that they correlate with diet[186,187]. We examined *CHIA*s in penguins using the same procedures as described for opsins and taste (umami) receptors above. In contrast to most examined birds, which have one to four intact *CHIA*s[188], penguins have a single pseudogenized *CHIA*. At first glance it is perplexing that penguins would lose *CHIAs*, as many species consume large amounts of crustaceans[189]. Fossil evidence, however, reveals that stem penguins focused primarily on larger prey items like fish and squid[24], and that adaptations for capturing smaller planktonic prey arose as recently as the Pliocene[24]. We propose that the two inactivating mutations shared by extant penguins (Fig. 5) evolved during a ~50 Ma interval during which stem penguins consumed little or no arthropod prey.

Comparison analyses
We obtained an ortholog genes dataset based on the whole genome alignment of all birds used in this study and extracted the positively selected genes and genes with penguin-specific substitutions from this dataset based on the gene annotation. We then made qualitative comparisons of these genes to compare their protein sites between penguins and other birds, and then summarised these using Weblogo. It is important to note that in some birds, some genes may be absent due to missing data, so that the number of taxa might be different for different comparisons (Supplementary Data 7).

Behavioural study of gentoo penguin vision
As a representative of penguins, we undertook a behavioural study on captive gentoo penguins at SEALIFE Kelly Tarlton's Aquarium, Auckland, New Zealand to examine their ability to see in the ultraviolet (UV) spectrum (SEALIFE ethics approval SL(G) AR001, and as non-native to New Zealand not subject to the provisions of the New Zealand Wildlife Act 1953). A Tank007 TK566 black OEM 365 nm torch (Shenzhen Grandoor Electronic Co., Ltd., China) was projected onto the snow in the penguin enclosure and penguins were observed to determine whether they would follow the movements of the torch's UV projection. The experiment was recorded using a GoPro (GoPro Inc., California, USA). The spectral output of the torch was measured using a USB2000+ spectrometer (Ocean Optics Inc., Florida, USA), which confirmed maximum output at approximately 365 nm, tapering to no output by 390 nm. None of the human observers were able to see the UV torch projection but at least five *Pygoscelis papua* penguins appeared to be able to follow the torch's projection. One penguin in particular was recorded following the torch projection being moved rapidly back and forth for an extended period of time. No such interest was displayed by any penguin when the torch was turned off despite it still being moved to and fro. This

experiment demonstrates that *P. papua* is able to see in the near UV spectrum (Supplementary Movie 1).

Supplementary Figures

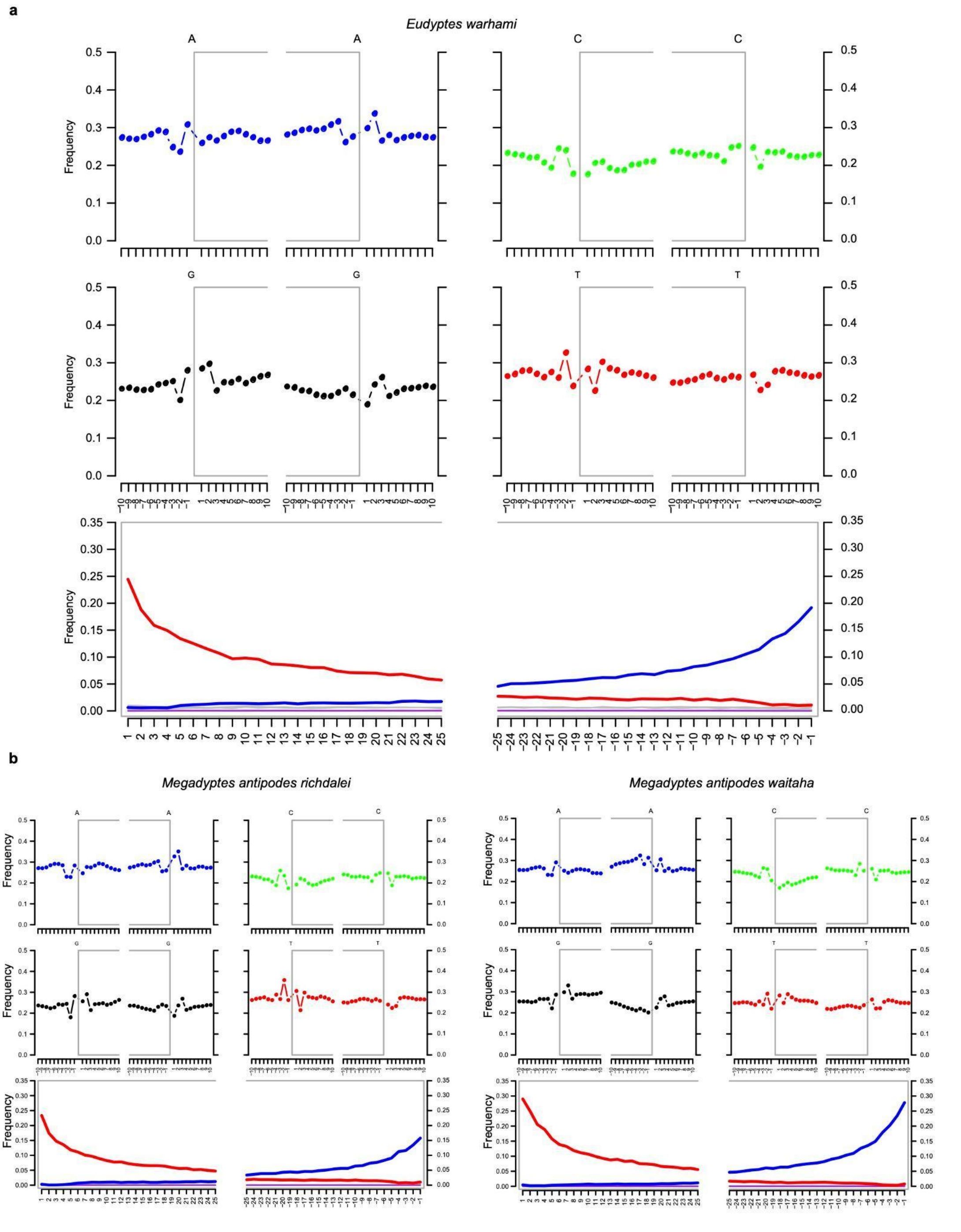

Supplementary Fig. 1 Authenticity of the uniquely mapped reads from three extinct penguins. a, From *Eudyptes warhami*. **b,** From *Megadyptes antipodes richdalei* and *Megadyptes antipodes waitaha*. Source data is provided as a Source Data file.

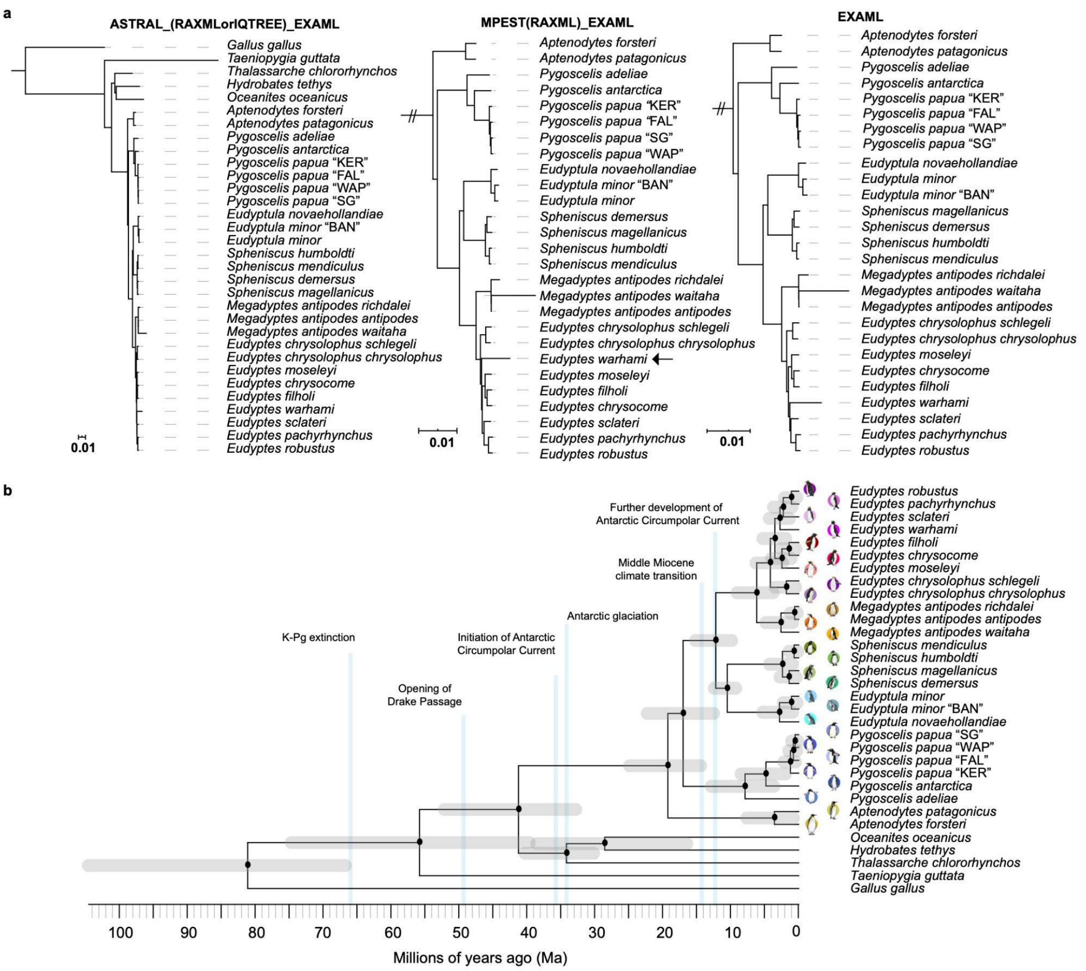

Supplementary Fig. 2 Phylogenetic tree construction. a, Phylogenomic reconstructions of penguins inferred by different methods using all genome data. The topology and support (bootstrap support: 100) was identical using ASTRAL-III, MP-EST, and ExaML. The majority of the dated trees have the same placement for *Eudyptes warhami* (Fig. 1) as in the ASTRAL tree and ExaML tree, while the MPEST tree supports a different relationship within *Eudyptes*. **b,** Calibrated phylogenetic tree of modern penguin taxa using genome data. All nodes had posterior probability >0.95. The 95% credible intervals for node ages are shown with transparent grey bars. In both (a) and (b) WAP West Antarctic Peninsula, SG South Georgia, KER Kerguelen, FAL Falkland/Malvinas, and BAN Banks Peninsula. Source data is provided as a Source Data file.

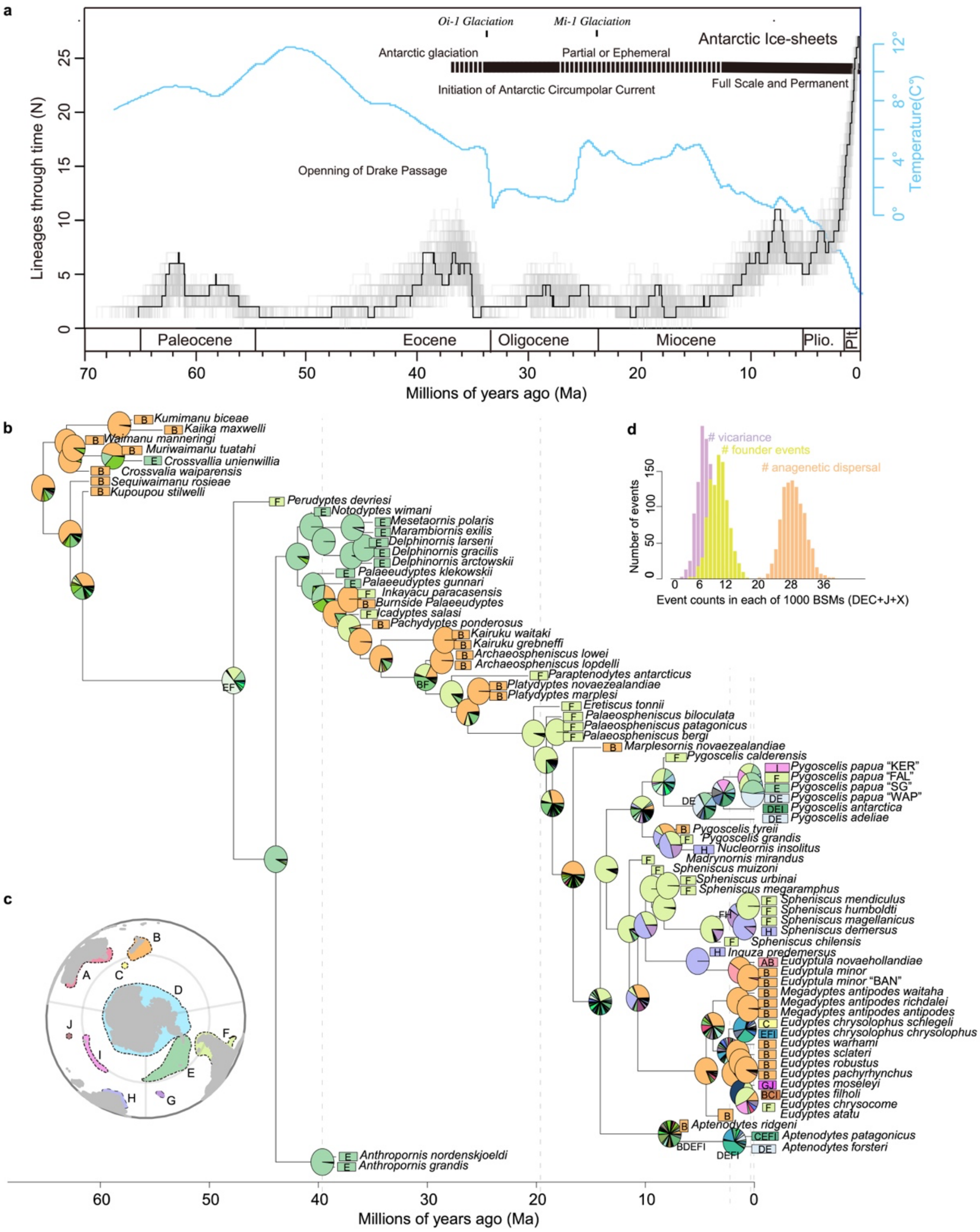

Supplementary Fig. 3 Lineages through time and ancestral range estimations of penguins under the best-fitting model (DEC+J+X). a, Lineages through time and global temperature. Global temperature trends and climate events were modified from 54. Penguins showed a persistent capacity of adaptation to climate cooling as all major diversification peaks occur during major cooling periods. Note: The incomplete fossil sampling may introduce biases to the lineages through time plot. **b,** Ancestral range estimations of penguins. Relative probabilities of each ancestral reconstruction are indicated with pie charts at each node. WAP West Antarctic Peninsula, SG South Georgia, KER Kerguelen, FAL Falkland/Malvinas, and BAN Banks Peninsula. **c,** Map of areas modified from [24]. **d,** Histograms of event counts from Biogeographical Stochastic Mapping. Most biogeographical events comprise dispersals, with only a few vicariant events. The high number of allopatric speciation events was expected given the large size of our regions. Source data is provided as a Source Data file.

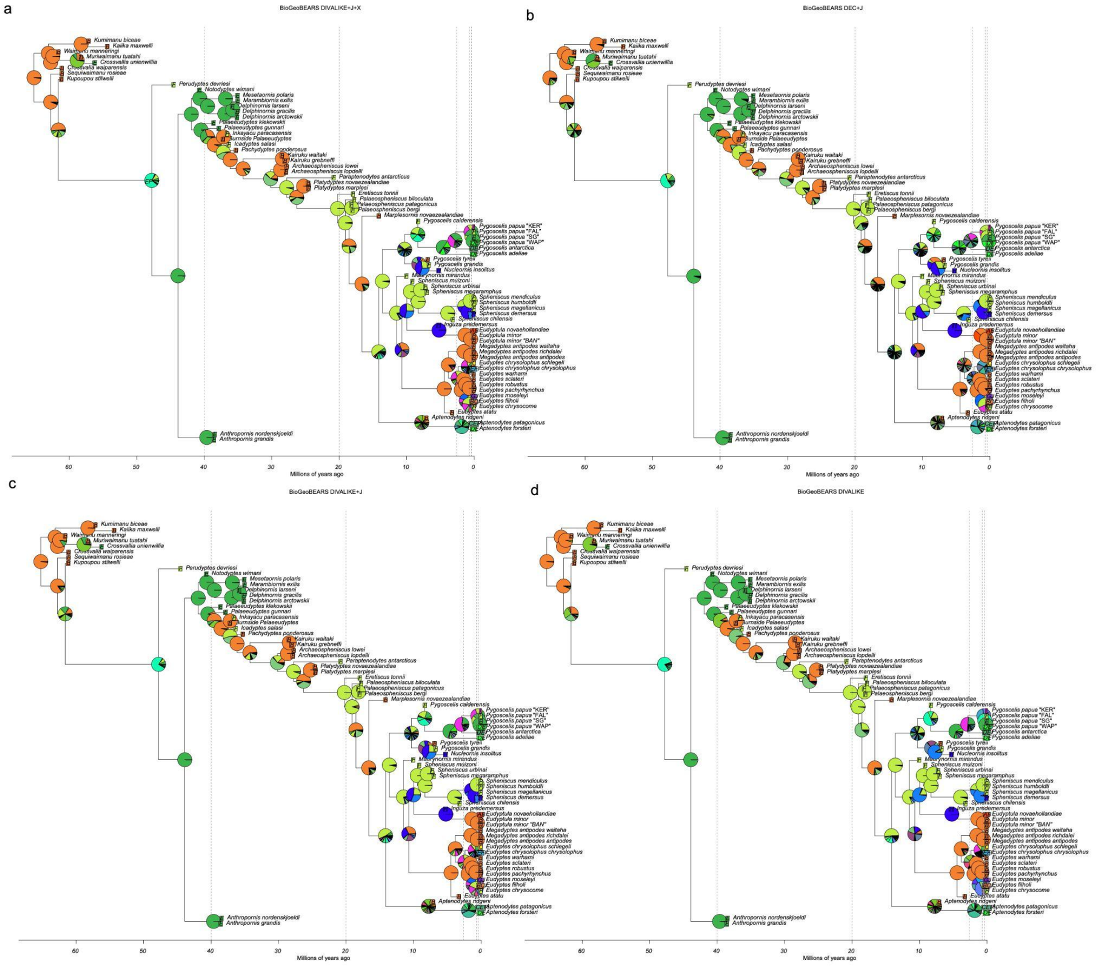

Supplementary Fig. 4 Ancestral range estimations of penguins under the preferred models. The relative probabilities of each ancestral reconstruction are indicated with pie charts at each node following the coding described in Supplementary Fig. 3c. Estimations under **(a)** DIVALIKE + j + x, **(b)** DEC+j, **(c)** DIVALIKE + j and **(d)** DIVALIKE models consistently suggest that stem penguins evolved in New Zealand, with the largest probability. Source data is provided as a Source Data file.

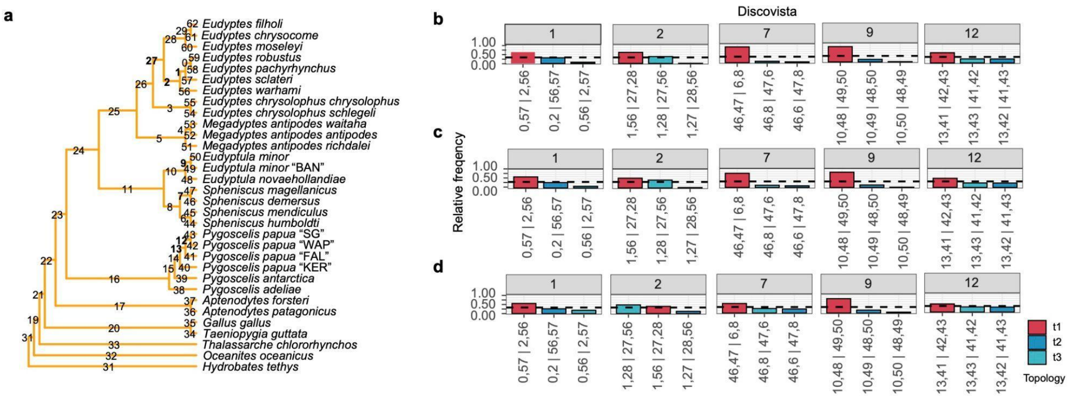

Supplementary Fig. 5 Discordance between species tree and gene trees. a, Consensus tree with the number of internal branches. The number of each subfigure in (b-d) indicates the label of the related internal branch on the tree in (a). b, Relative frequency distribution. The frequencies of the three topologies around each internal branch were calculated using DiscoVista, with gene trees inferred from 100 kb windows using RaxML. The main topology is shown with a red bar and the two alternative topologies are shown with blue bars. On the x-axis, the exact definition of each quartet topology is provided using the neighboring branch labels separated by "|". If the discordance is purely due to ILS, then it is expected that the second and third hypotheses will have similar frequencies. c, Relative frequency distribution inferred from 50 kb windows using IQtree. d, Relative frequency distribution inferred from 5 kb windows using IQtree. Source data is provided as a Source Data file.

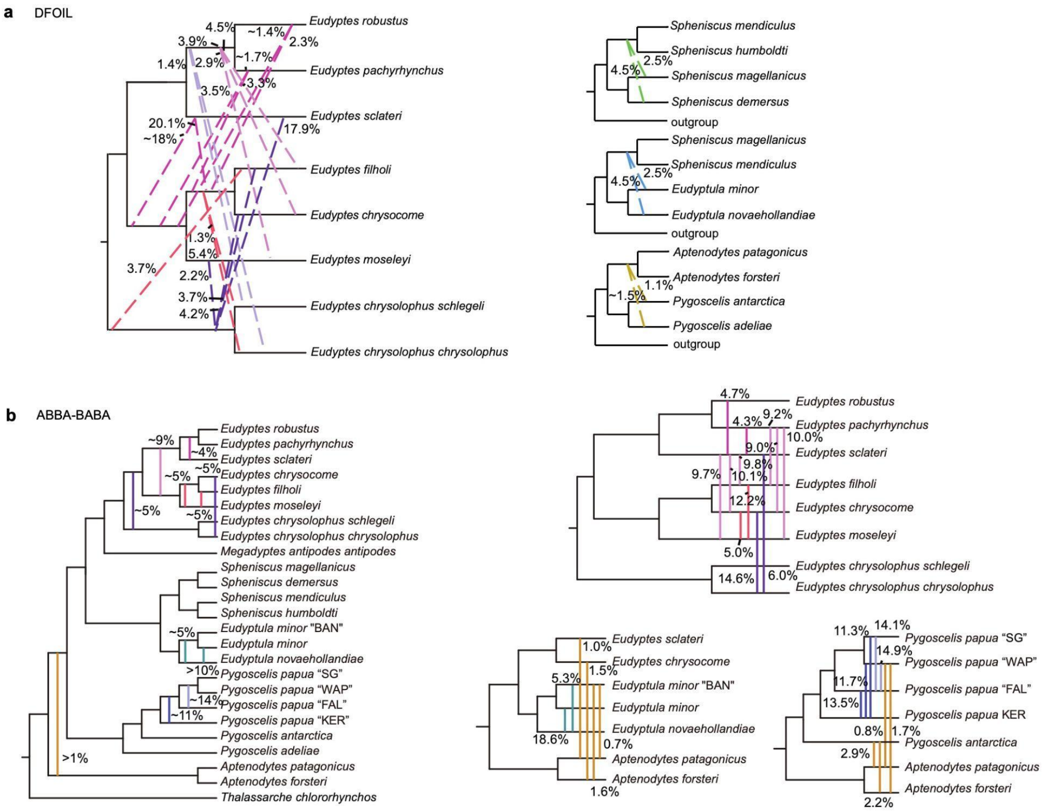

Supplementary Fig. 6 Introgression results detected by DFOIL and ABBA-BABA test. a, DFOIL test results. Introgression was detected within the genus *Eudyptes*, within the genus *Spheniscus*, between *Spheniscus* and *Eudyptula* and between *Pygoscelis* and *Aptenodytes*. b, Summary of introgression (left) recovered from ABBA-BABA test result according to the parsimony principle and the ABBA-BABA test results (right) of introgression among all combinations. Source data is provided as a Source Data file.

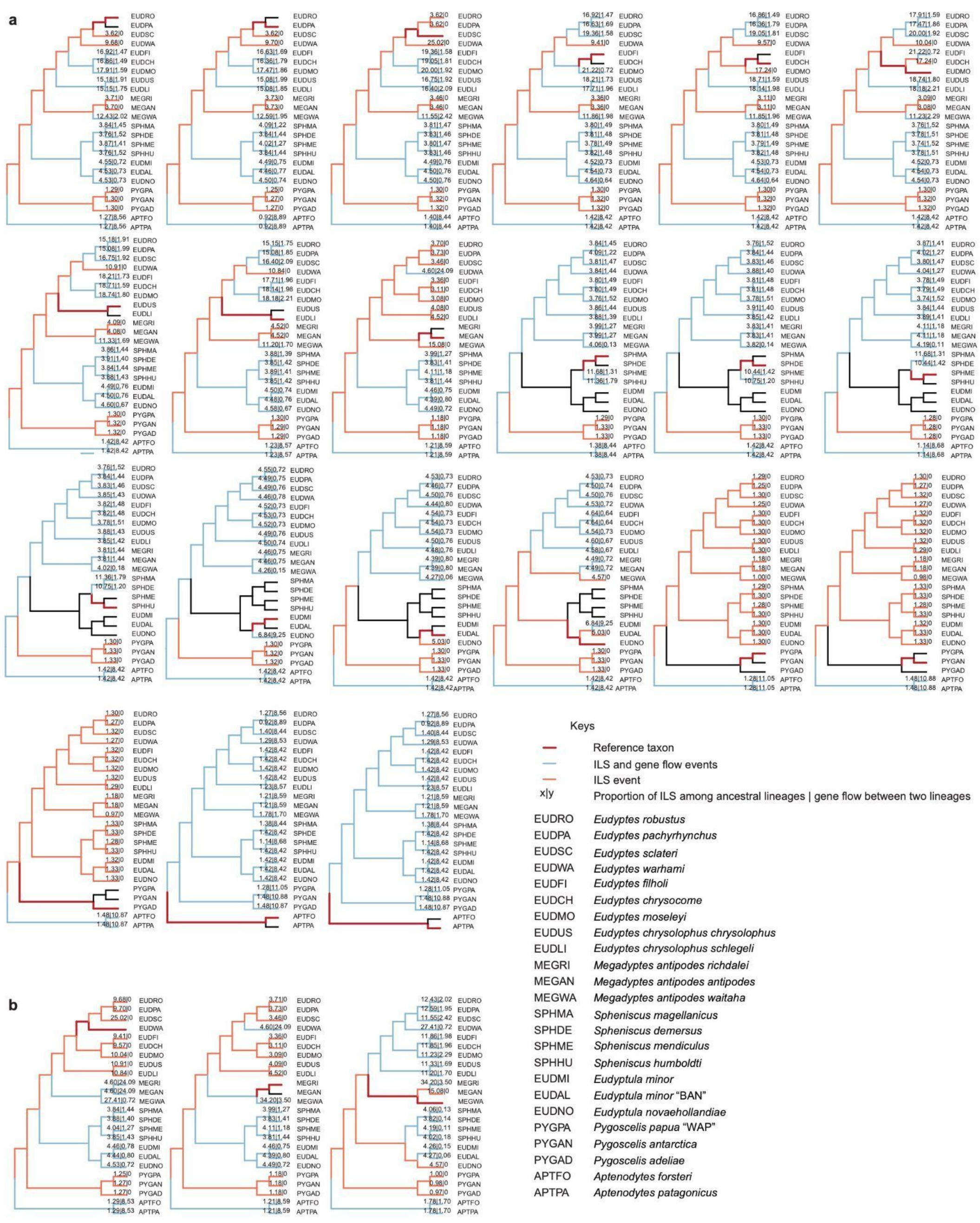

Supplementary Fig. 7 QuIBL results for each lineage. Results for the reference lineages from (a) extant lineages, and (b) extinct lineages. Average proportions of incomplete lineage sorting (ILS) within the ancestors or the introgression between the reference lineage and other lineages identified by QuIBL are shown near the tips of the branches. Reference lineage for each tree was marked with the red branch line. Source data is provided as a Source Data file.

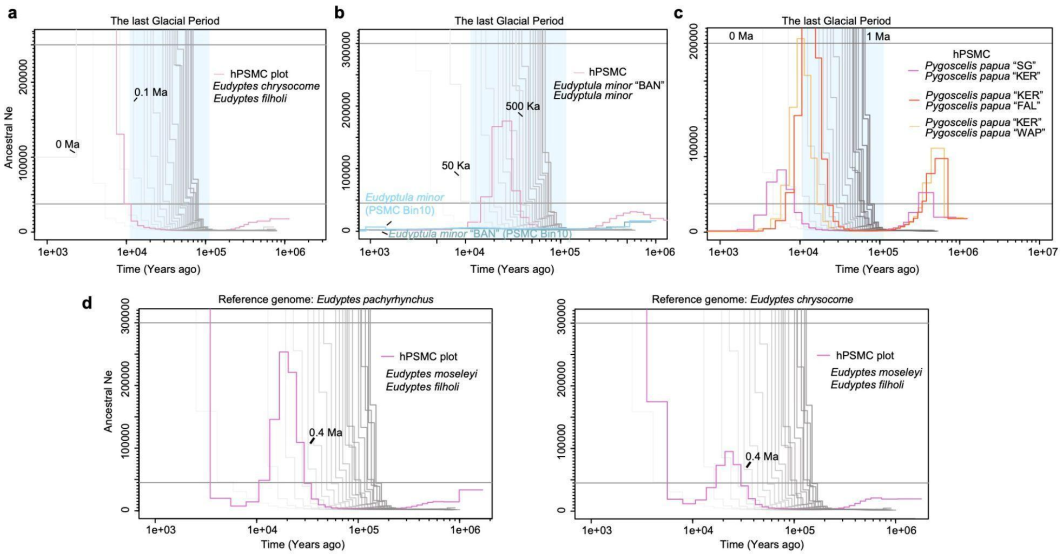

Supplementary Fig. 8 Determination of the cessation of introgression using hPSMC. **a**, Determination of the cessation of introgression for *Eudyptes chrysocome* and *Eudyptes filholi* using hPSMC. The end of the post-divergence introgression occurred within 0.1 Ma. The hPSMC plot generated for the artificially created hybrid genomes are compared with 21 simulated datasets spanning 0–1 Ma in 50,000-year intervals. The horizontal lines delineate the 1.5–10 times the pre-divergence effective population size (Pre-Ne). **b**, Determination of the cessation of introgression for *Eudyptula minor* and *E. minor* (Banks Peninsula: BAN) lineages using hPSMC. The hPSMC curve (pink) increased and then decreased during the Last Glacial Period (sky-blue background). The effective population size of the hybrid at the rising stage was much larger than the effective population size of the individuals (blue curves) as expected. The two lineages were likely isolated but then recontacted. Results suggest the post-divergence introgression still occurs between the two lineages. **c**, Determination of the cessation of introgression for *Pygoscelis papua* lineages (Kerguelen: KER, Falkland/Malvinas: FAL, South Georgia: SG, West Antarctic Peninsula: WAP) using hPSMC. Post-divergence introgression still occurs between the lineages. **d**, hPSMC results for *Eudyptes moseleyi* and *E. filholi*. The hPSMC plot generated for the artificially created hybrid genomes are compared with simulated datasets of different divergence times spanning 0–2 Ma in 100,000-year intervals. Two runs with different reference genomes resulted in an identical result for the cessation of introgression. Source data is provided as a Source Data file.

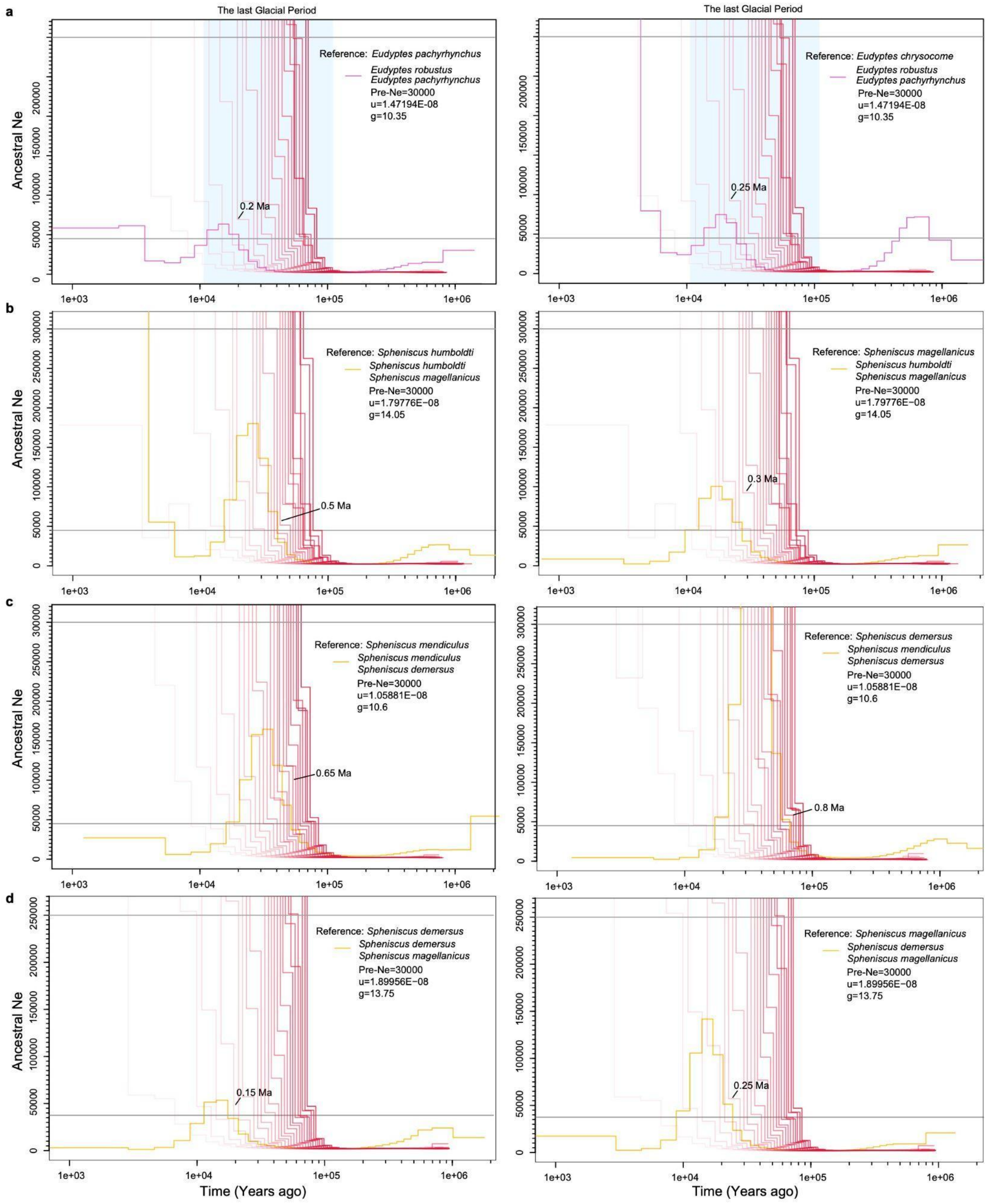

Supplementary Fig. 9 Divergence time estimation for five species pairs using hPSMC with different reference genomes. The hPSMC results for (a) *E. robustus* and *E. pachyrhynchus*, (b) *Spheniscus humboldti* and *S. magellanicus*, (c) *S. mendiculus* and *S. demersus,* and (d) *S. demersus* and *S. magellanicus*. The hPSMC plot generated for the artificially created hybrid genomes are compared with simulated datasets of different divergence times spanning 0–1 Ma in 50,000-year intervals. The horizontal lines delineate the 1.5–10 times the pre-divergence effective population size. The pre-divergence effective population size ("Pre-Ne" in short) was set as 30 k in simulation analyses (a)–(c), and 25 k for (d). For each pair, two runs with different reference genomes resulted in an identical result for the cessation of introgression. Source data is provided as a Source Data file.

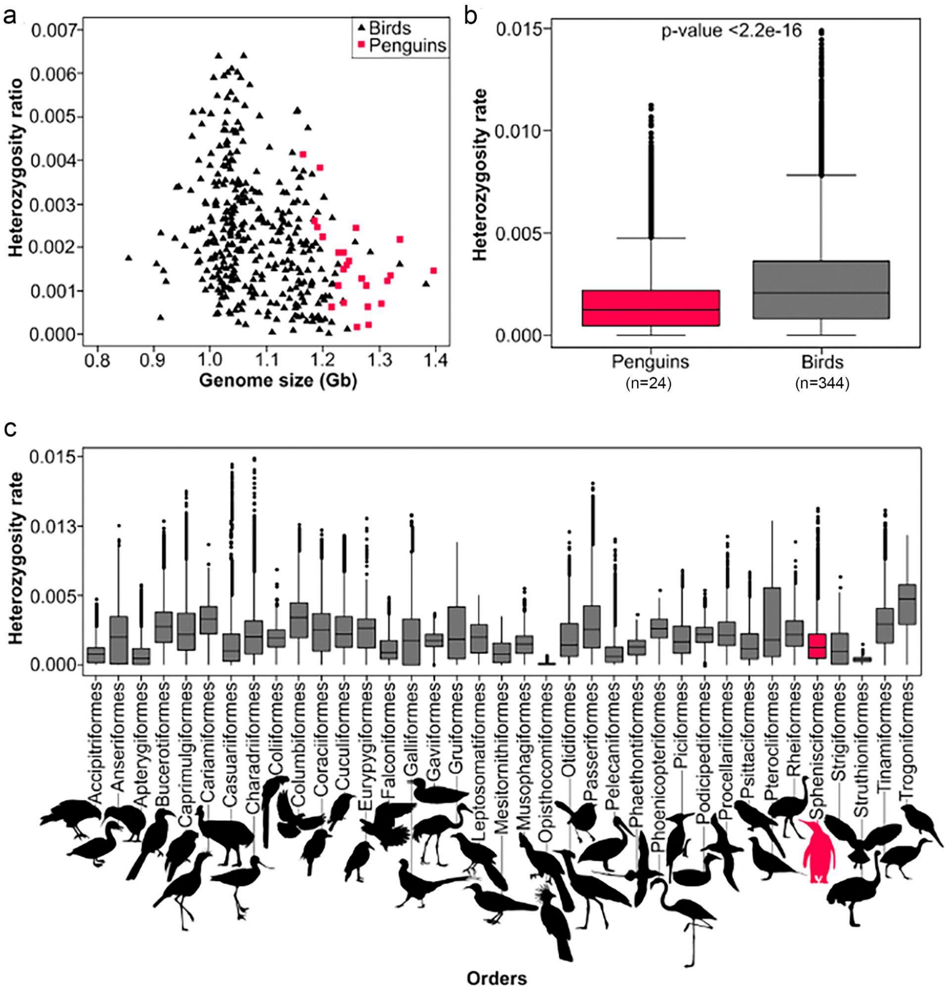

Supplementary Fig. 10 Heterozygosity. a, a scatter diagram for heterozygosity rate between penguins and >300 other birds. **b,** a box plot for the combined heterozygosity rate between penguins (n=24) and >300 other birds (one sided Wilcoxon Rank sum test, P-value < 2.2e-16). **c,** the combined heterozygosity rate of 100 kb slided windows was compared at order level. Boxplots show the median with hinges at the 25th and 75th percentile and whiskers extending 1.5 times the interquartile range. Source data is provided as a Source Data file.

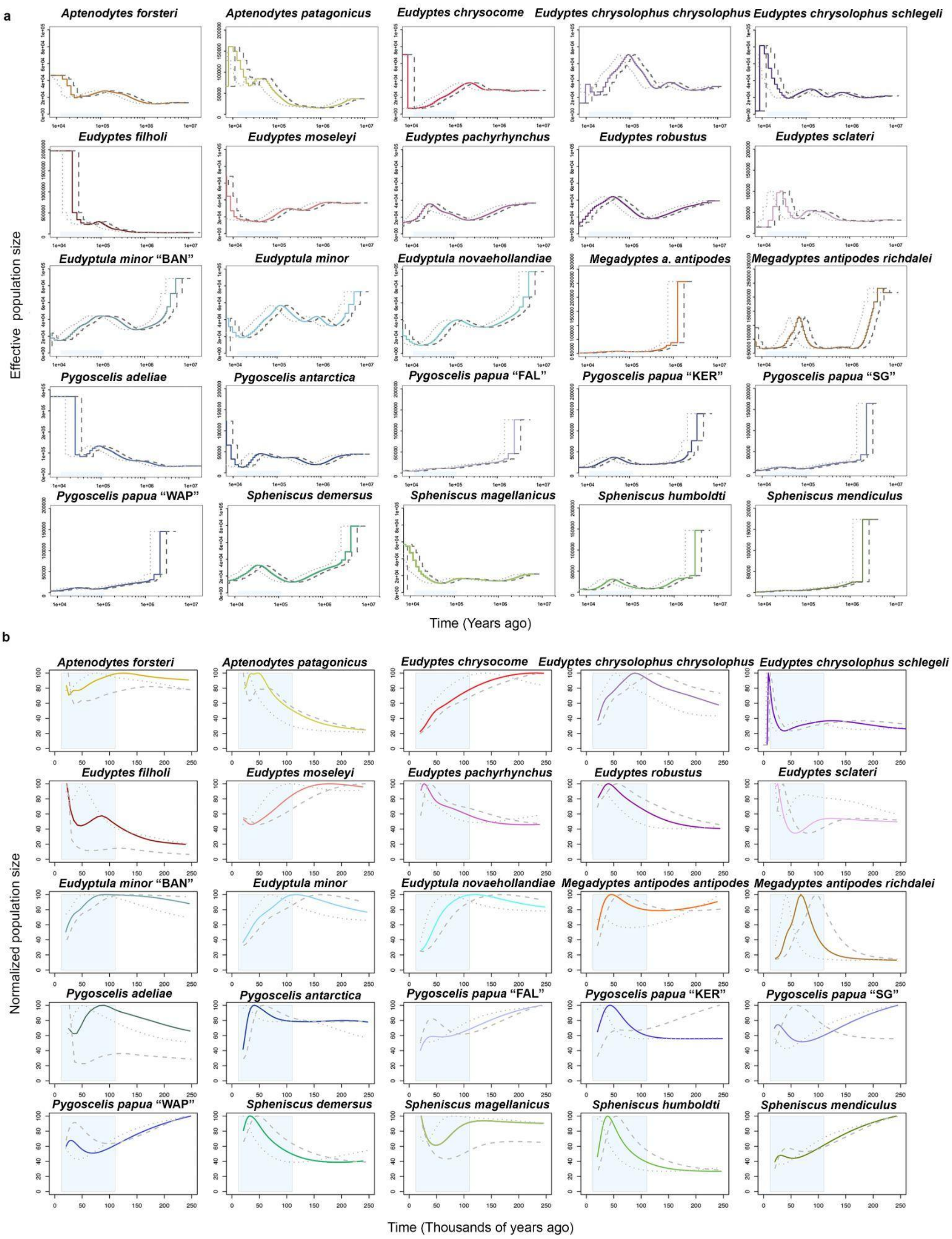

Supplementary Fig. 11 PSMC results with 40% error (pertaining to uncertainties in applied generation times). a, PSMC plots for all penguins with 40% error. The 40% error was included in grey dashed lines (remove 40% in small dotted lines, add 40% in wide dotted lines). **b**, Normalized PSMC plots for the 20–250 Ka with 40% error (remove 40% in small dotted lines, add 40% in wide dotted lines). The blue bar in the background in a, and b, is the Last Glacial Period. Source data is provided as a Source Data file.

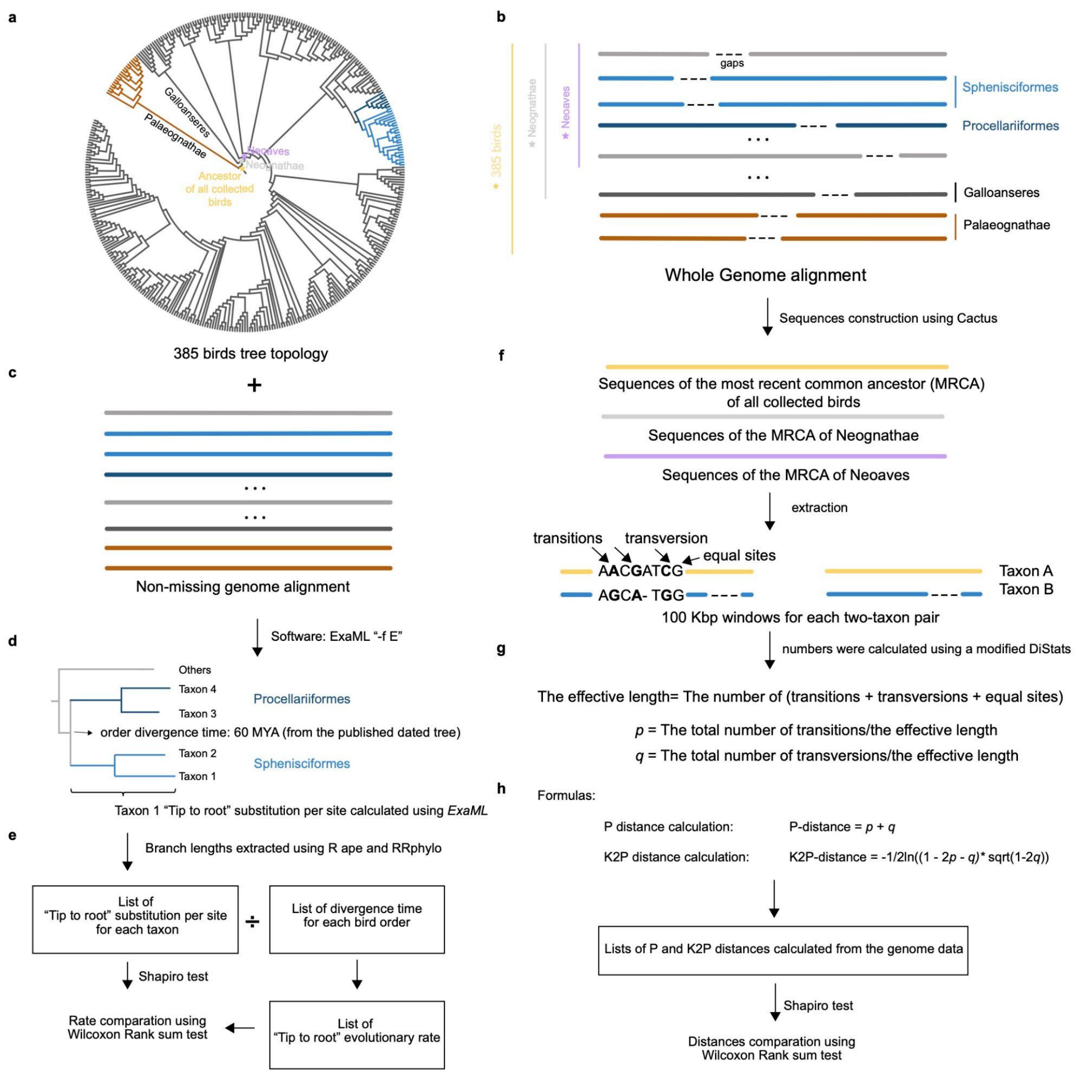

Supplementary Fig. 12 The flowchart of P distances, K2P distances, substitution per site and evolutionary rate calculation.

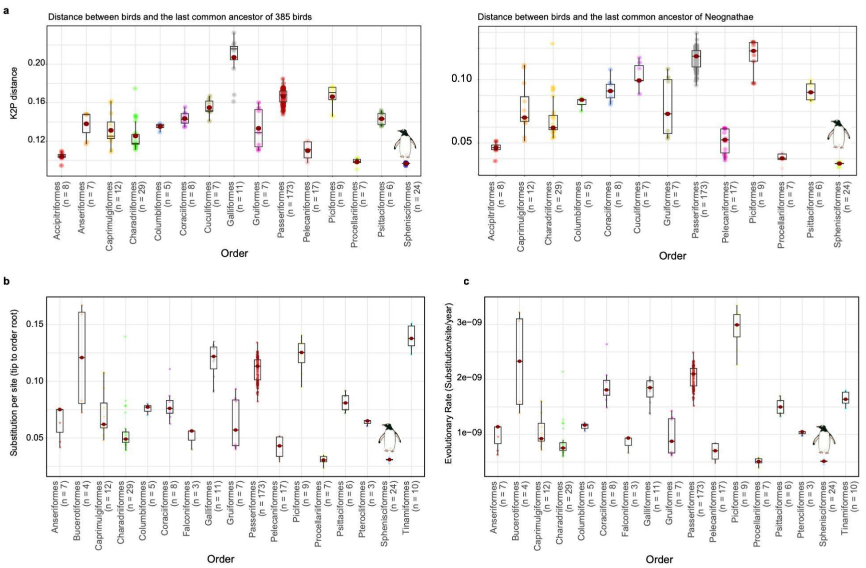

Supplementary Fig. 13 Evolutionary comparison. a, Distribution of k2p distance between birds and the last common ancestor of 385 birds or the last common ancestor of Neoaves. **b,** Distribution of substitution per site. c, Distribution of evolutionary rate. Source data is provided as a Source Data file.

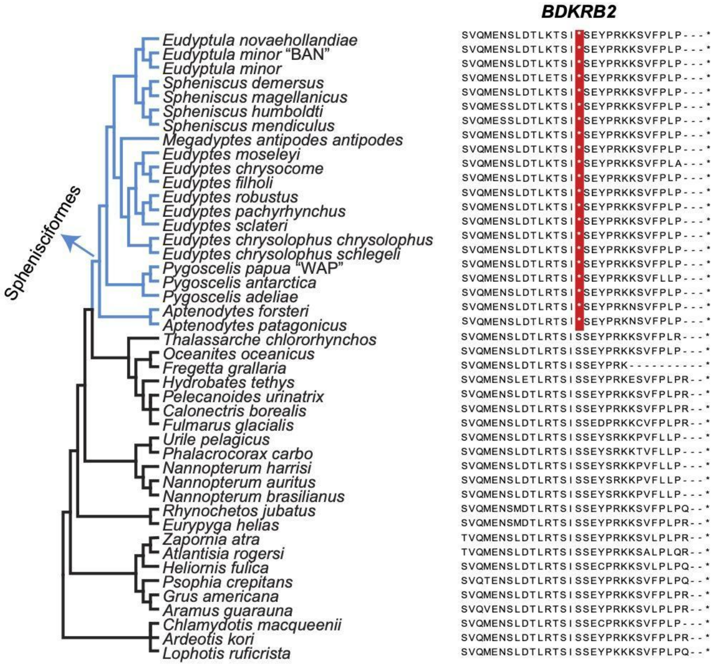

Supplementary Fig. 14 Pseudogene *BDKRB2*. The partial multiple sequence alignment of *BDKRB2*. Contrasting [31] who found *BDKRB2* to be positively selected among penguins, we detected a premature stop codon in exon 2, suggesting inactivation of this gene in penguins.

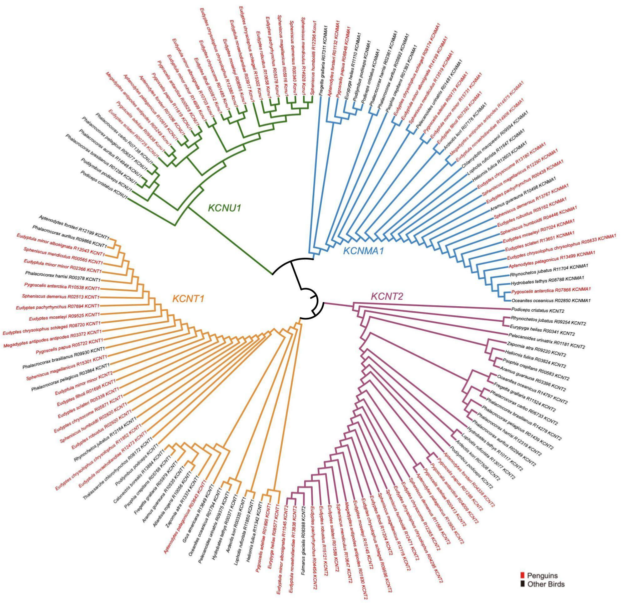

Supplementary Fig. 15 Gene expansion. A consensus tree for *KCNMA1*, *KCNT1*, *KCNT2* and *KCNU1* calcium channel genes, which we relate to bone density. Source data is provided as a Source Data file.

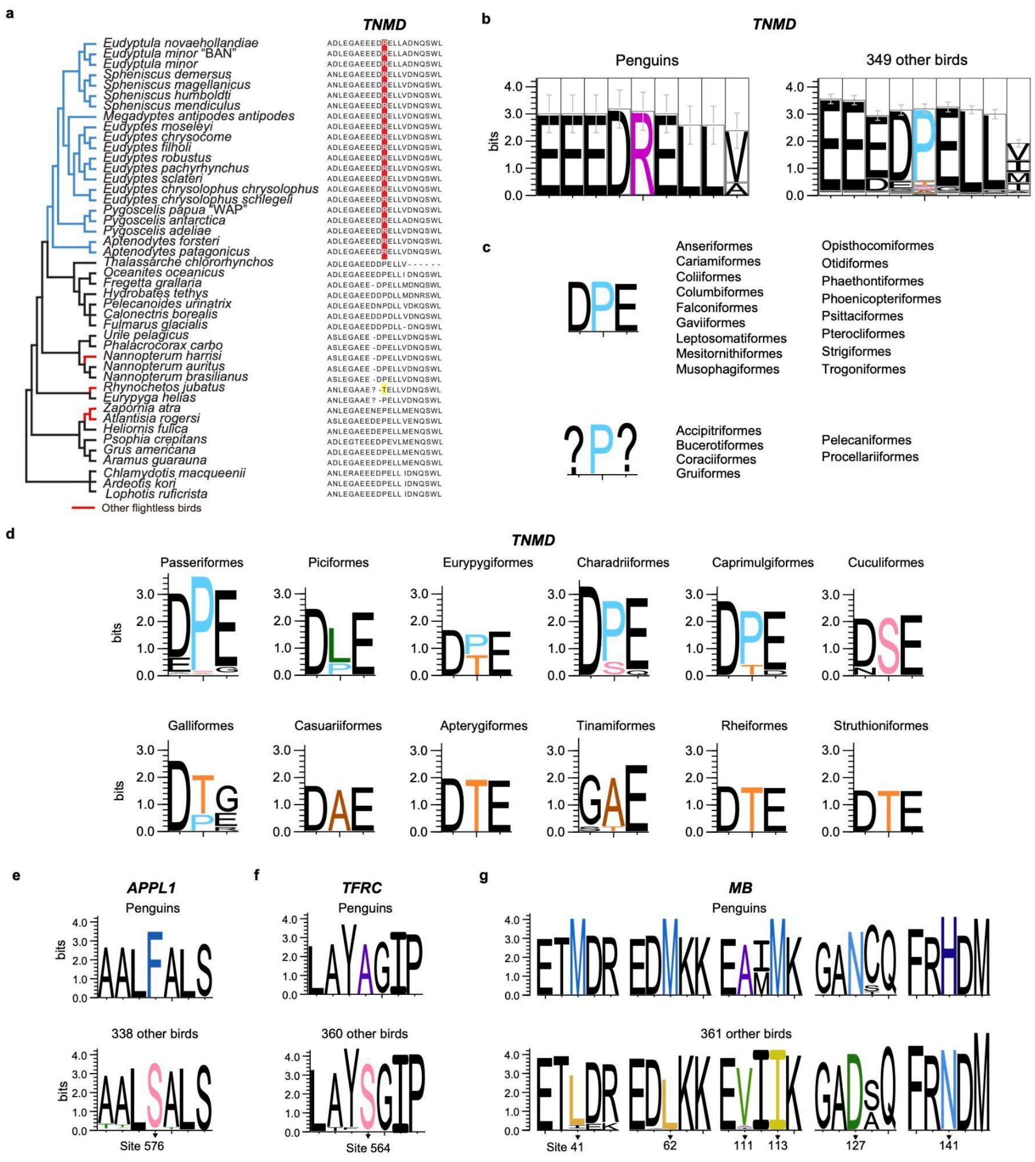

Supplementary Fig. 16. Genes involved in penguin specific adaptations. a, Topology of species used in flightlessness analysis (red branches were tested) and the partial multiple sequences alignment of *TNMD*. **b,** comparison of penguin-specific substitution in *TNMD* between penguins and other birds. **S**everal orders have a Pro **(c)** while other orders have more amino acids at that site (d). More comparisons of the penguin-specific substitutions in genes (e) *APPL1*, (f) *TFRC* and (g) *MB*. Source data is provided as a Source Data file.

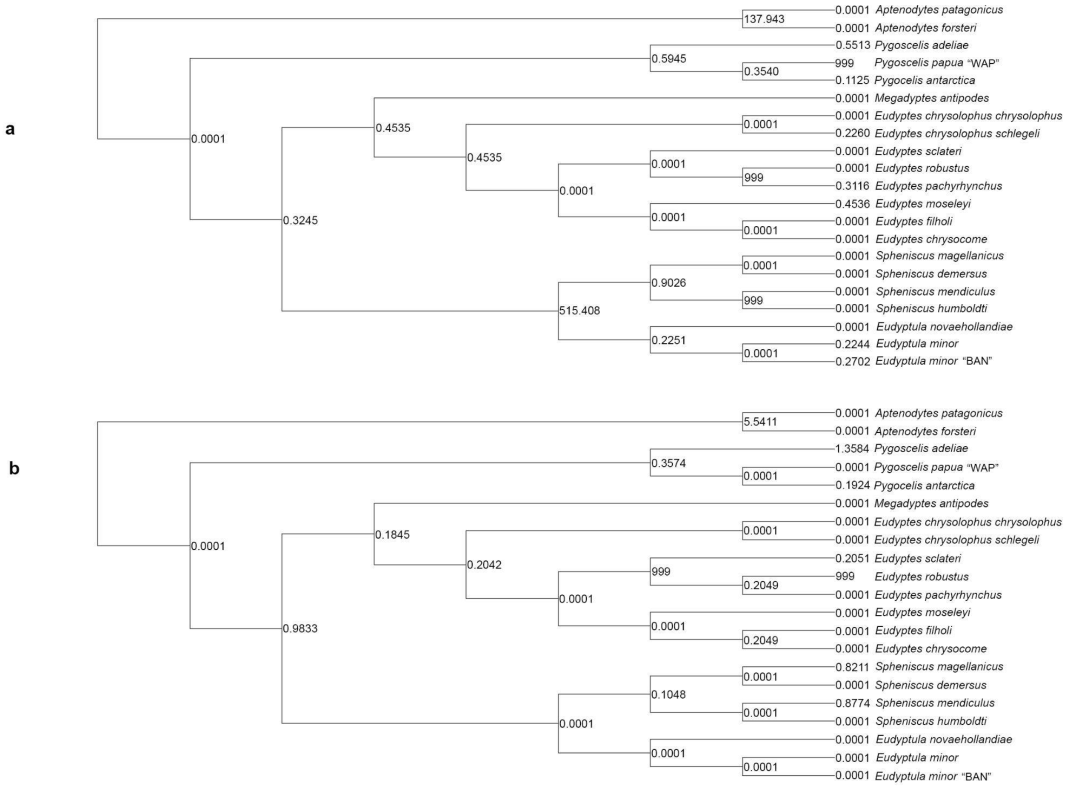

Supplementary Fig. 17 Free ratio test trees for *ANO6* and *FIBB*. (a) is *FIBB* and **(b)** is *ANO6*. Branch labels >1 indicate positive selection. Branch labels 999 indicate there is not enough data to determine the strength of selection. Source data is provided as a Source Data file.

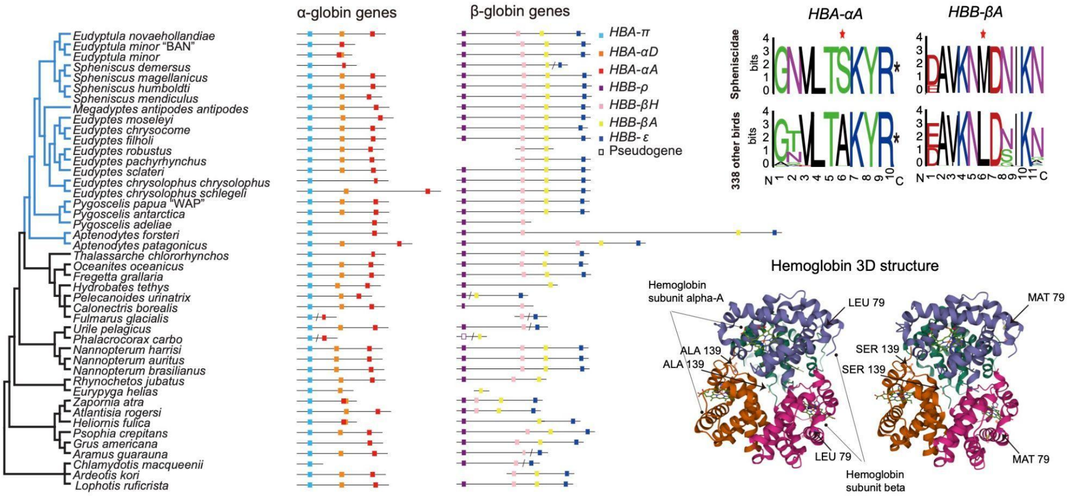

Supplementary Fig. 18 α and β-globin hemoglobin gene annotation, the mutation sites in *HBB*-βA and *HBA*-αA and inferred hemoglobin protein 3D structure. At site 139 of hemoglobin subunit alpha-A, penguins have a Ser while the chicken has an Ala, and this site creates intersubunit contact and increases the stabilization of the relaxed state (R-state) conformation [70]. At site 79 of hemoglobin subunit beta, penguins have a Met, and chicken have a Leu. Source data is provided as a Source Data file.

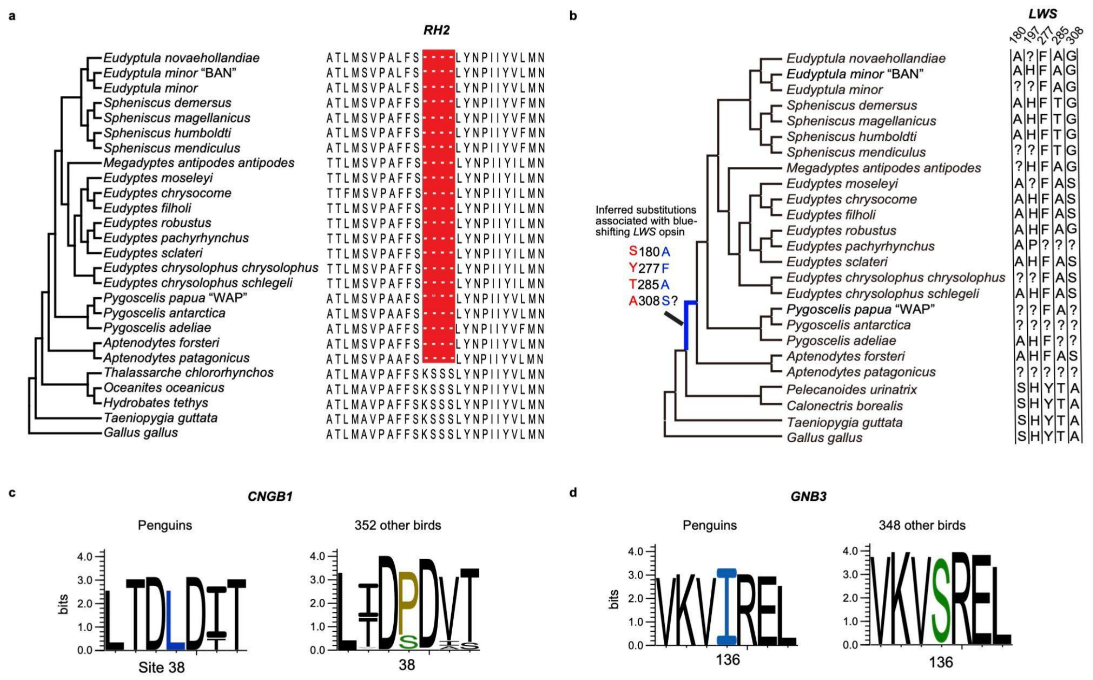

Supplementary Fig. 19 Genes involved in penguin vision adaptation. a, Deletion in the green cone opsin gene *(RH2)* of the crown penguin lineages. The inactivation of *RH2* in penguins is inferred from a 12 bp deletion. **b,** Phylogenetic distribution of LWS key tuning sites in penguins and outgroup species. Putative blue-shift substitutions on stem Sphenisciformes branch inferred by parsimony and derived from direct measurements of LWS opsin spectral sensitivity (Table 2 in [183]). **c,** Comparisons of the penguin-specific substitutions in genes **(c)** *CNGB1* and **(d)** *GNB3*. Source data is provided as a Source Data file.

Description of Additional Supplementary Files

Supplementary Data 1 The basic information for assembly, annotation, morphological characters, fossil calibrations, foraging distances, genetic distances and phylogenetic analyses. Biogeobears models for the evolution of penguins geographic ranges reconstruction were compared with Likelihood Ratio Test and Akaike information criterion, and the P values of the null hypothesis testing were shown in the table.

Supplementary Data 2 Introgression and ILS quantification results, penguin evolutionary rate and heterozygosity rate. One sided T-tests or Wilcoxon-tests were performed for heterozygosity rate comparison for penguins (n=24) and other avian orders (n>3) and P-values were shown in the table.

Supplementary Data 3 Evolutionary rate comparison and repeat elements.

Supplementary Data 4 Genes involved in penguin specific adaptations from all methods. Genes were identified under branch, branch-site or site models using PAML. P values were calculated using the chi-square test and P-values were adjusted by Benjamini and Hochberg method.

Supplementary Data 5 Phylogenomic dataset. S5.1 Morphological dataset. The full matrix contains 72 fossil and extant penguin taxa, two outgroup taxa, and 281 morphological characters. We expanded the morphological dataset of [24], including a morphological tree and the morphological matrix by incorporating several additional fossil penguin species, including *Crossvalia waiparensis*, *Crossvallia unienwillia*, *Kupoupou stilwelli*, and *Kaiika maxwelli,* and seven additional morphological characters. S5.2 Phylogenomic trees inferred from all methods. S5.3 Datasets for dating analyses. The configuration files for the node-dating and the Bayesian total-evidence dating analyses and the dating results from the dating analyses. S5.4 Randomly selected trees for densitree plot.

Supplementary Data 6 Introgression analyses dataset. S6.1 Introgression analyses Commands. S6.2 Results from hPSMC analysis.

Supplementary Data 7 Genes involved in penguin specific adaptations. Protein structure files of myoglobin proteins and hemoglobin proteins. S7.1 Comparison of penguin-specific substitutions between penguins and other birds. S7.2 Protein structure files of myoglobin proteins and hemoglobin proteins. The penguin-specific amino acid substitutions may be responsible for the stabilization in penguin myoglobin and hemoglobin proteins.

Supplementary Software. Codes used in this study.

Supplementary References

1. Cole, T. L. et al. Mitogenomes uncover extinct penguin taxa and reveal island formation as a key driver of speciation. *Mol. Biol. Evol* **36**, 784–797 (2019a).

2. Boessenkool, S. et al. Relict or colonizer? Extinction and range expansion of penguins in southern New Zealand. *P. Roy. Soc. B-Biol. Sci* **276,** 815–821 (2009).

3. Rawlence, N. J. et al. Radiocarbon-dating and ancient DNA reveal rapid replacement of extinct prehistoric penguins. *Quat. Sci. Rev* **112,** 59–65 (2015).

4. Rawlence, N. J., Tennyson, A. J. D., Cole, T. L., Verry, A. J. F. & Scofield, R. P. Evidence for breeding of *Megadyptes* penguins in the North Island at the time of human arrival. *New Zeal J. Zool* **46,** 165–173 (2019).

5. Li, C. et al. Two Antarctic penguin genomes reveal insights into their evolutionary history and molecular changes related to the Antarctic environment. *Gigascience* **3**, 27 (2014).

6. Pan, H. et al. High-coverage genomes to elucidate the evolution of penguins. *Gigascience* **8**, giz117 (2019).

7. Clucas, G. V. et al. Comparative population genomics reveals key barriers to dispersal in Southern Ocean penguins. *Mol. Ecol* **27,** 4680–4697 (2018).

8. Tyler, J., Bonfitto, M. T., Clucas, G. V., Reddy, S. & Younger, J. L. Morphometric and genetic evidence for four species of gentoo penguin. *Ecol. Evol* **10,** 13836–13846 (2020).

9. Vianna, J. A. et al. Marked phylogeographic structure of Gentoo penguin reveals an ongoing diversification process along the Southern Ocean. *Mol. Phylogenet. Evol* **107,** 486–498 (2017).

10. Seppey, M., Manni, M. & Zdobnov, E. M. BUSCO: assessing genome assembly and annotation completeness. *Methods Mol. Biol* **1962,** 227–245 (2019).

11. Stanke, M. et al. AUGUSTUS: *ab initio* prediction of alternative transcripts. *Nucleic Acids Res* **34,** W435–W439 (2006).

12. Weisenfeld, N. I., Kumar, V., Shah, P., Church, D. M. & Jaffe, D. B. Direct determination of diploid genome sequences. *Genome Res* **27,** 757–767 (2017).

13. Damgaard, P. B. et al. Improving access to endogenous DNA in ancient bones and teeth. *Sci. Rep* **5,** 11184 (2015).

14. Carøe, C. et al. Single-tube library preparation for degraded DNA. *Methods Ecol. Evol* **9,** 410–419 (2018).

15. Kircher, M., Sawyer, S. & Meyer, M. Double indexing overcomes inaccuracies in

multiplex sequencing on the Illumina platform. *Nucleic Acids Res* **40,** e3 (2012).

16. Schubert, M. et al. Characterization of ancient and modern genomes by SNP detection and phylogenomic and metagenomic analysis using PALEOMIX. *Nat. Protoc* **9,** 1056–1082 (2014).

17. Schubert, M., Lindgreen, S. & Orlando, L. AdapterRemoval v2: rapid adapter trimming, identification, and read merging. *BMC Res. Notes* **9,** 88 (2016).

18. Li, D. & Durbin, R. Fast and accurate short read alignment with Burrows-Wheeler transform. *Bioinformatics* **25,** 1754–1760 (2009).

19. Schubert, M. et al. Improving ancient DNA read mapping against modern reference genomes. *BMC Genomics* **13,** 178 (2012).

20. Jónsson, H., Ginolhac, A., Schubert, M., Johnson, P. L. F. & Orlando, L. mapDamage2.0: fast approximate Bayesian estimates of ancient DNA damage parameters. *Bioinformatics* **29,** 1682–1684 (2013).

21. Li, H. et al. The sequence alignment/map format and SAMtools. *Bioinformatics*, **25,** 2078–2079 (2009).

22. Danecek, P. & McCarthy, S. A. BCFtools/csq: haplotype-aware variant consequences. *Bioinformatics* **33,** 2037–2039 (2017).

23. Feng, S. et al. Dense sampling of bird diversity increases power of comparative genomics. *Nature* **587,** 252–257 (2020).

24. Thomas, D. et al. Ancient crested penguin constrains timing of recruitment into seabird hotspot. *P. Roy. Soc. B-Biol. Sci* **287**, 20201497 (2020).

25. Garcia Borboroglu, P. & Boersma, P. D. P*enguins: natural history and conservation* (Seattle: Washington, 2013).

26. Altschul, S. F., Gish, W., Miller, W., Myers, E. W. & Lipman, D. J. Basic local alignment search tool. *J. Mol. Biol* **215,** 403–410 (1990).

27. Birney, E., Clamp, M. & Durbin, R. GeneWise and genomewise. *Genome Res* **14,** 988–995 (2004).

28. Baker, A. J., Pereira, S. L., Haddrath, O. P. & Edge, K.-A. Multiple gene evidence for expansion of extant penguins out of Antarctica due to global cooling. *P. Roy. Soc. B-Biol. Sci* **273**, 1582 (2006).

29. Subramanian, S., Beans-Picón, G., Swaminathan, S. K., Millar, C. D. & Lambert, D. M. Evidence for a recent origin of penguins. *Biol. Lett* **9**, 20130748 (2013).

30. Gavryushkina, A. et al. Bayesian total-evidence dating reveals the recent crown radiation of penguins. *Syst. Biol* **66**, 57–73 (2017).

31. Vianna, J. A. et al. Genome-wide analysis reveals drivers of penguin diversification. *P. Natl. Acad. Sci. U.S.A* **36**, 22303–22310 (2020).

32. Bertelli, S. & Giannini, N. P. A phylogeny of extant penguins (Aves: Sphenisciformes) combining morphology and mitochondrial sequences. *Cladistics* **21**, 209–239 (2005).

33. Ksepka, D. T., Bertelli, S. & Giannini, N. P. The phylogeny of the living and fossil Sphenisciformes (penguins). *Cladistics* **22**, 412–441 (2006).

34. Cole, T. L. et al. Ancient DNA reveals that the 'extinct' Hunter Island penguin (*Tasidyptes hunteri*) is not a distinct taxon. *Zool. J. Linn. Soc. Lond* **182,** 459–464 (2018).

35. Cole, T. L. et al. Ancient DNA of crested penguins: Testing for temporal genetic shifts in the world's most diverse penguin clade. *Mol. Phylogenet. Evol* **131,** 72–79 (2019c).

36. Slack, K. E. et al. Early penguin fossils, plus mitochondrial genomes, calibrate avian evolution. *Mol. Biol. Evol* **23**, 1144–1155 (2006).

37. Ksepka, D. T. & Ando, T. *Penguins past, present, and future: trends in the evolution of the Sphenisciformes*. In *Living dinosaurs: the evolutionary history of modern birds* (Oxford, Wiley, 2011).

38. Paten, B. et al. Cactus: Algorithms for genome multiple sequence alignment. *Genome Res* **21,** 1512–1528 (2011).

39. Tarailo-Graovac, M. & Chen, N. Using RepeatMasker to identify repetitive elements in genomic sequences. *Curr. Protoc. Bioinformatics* **25,** 4–10 (2009).

40. Liu, L., Yu, L. & Edwards, S. V. A maximum pseudo-likelihood approach for estimating species trees under the coalescent model. *BMC Ecol. Evol* **10,** 302 (2010).

41. Zhang, C., Rabiee, M., Sayyari, E. & Mirarab, S. ASTRAL-III: Polynomial time species tree reconstruction from partially resolved gene trees. *BMC Bioinformatics* **19,** 153 (2018).

42. Kozlov, A. M., Aberer, A. J. & Stamatakis, A. ExaML version 3: A tool for phylogenomic analyses on supercomputers. *Bioinformatics* **31,** 2577–9 (2015).

43. Stamatakis, A. RAxML version 8: a tool for phylogenetic analysis and post-analysis of large phylogenies. *Bioinformatics* **30,** 1312–1313 (2014).

44. Nguyen, L.-T., Schmidt, H. A., von Haeseler, A. & Minh, B. Q. IQ-TREE: A fast and effective stochastic algorithm for estimating maximum-likelihood phylogenies. *Mol. Biol. Evol* **32,** 268–274 (2015).

45. Sackton, T. B. et al. Convergent regulatory evolution and loss of flight in paleognathous birds. *Science* **364,** 74–8 (2019).

46. Bouckaert, R. et al. BEAST 2: A Software Platform for Bayesian Evolutionary Analysis. *PLoS Comp. Biol* **10,** e1003537 (2014).

47. Bouckaert R. et al. BEAST 2.5: An advanced software platform for Bayesian evolutionary analysis. *PLoS Comp. Biol* **15,** e1006650 (2019).

48. Rambaut, A., Drummond, A. J., Xie, D., Baele, G. & Suchard, M. A. Posterior summarization in Bayesian phylogenetics using Tracer 1.7. Syst. Biol **67,** 901–904 (2018).

49. Stadler, T. Sampling-through-time in birth–death trees. *J. Theor, Biol* **267**, 396–404 (2010).

50. Heath, T. A., Huelsenbeck, J. P. & Stadler, T. The fossilized birth-death process for coherent calibration of divergence-time estimates. *P. Natl. Acad. Sci. U.S.A* **111**, E2957–E2966 (2014).

51. Lewis, P. O. A likelihood approach to estimating phylogeny from discrete morphological character data. *Syst. Biol.* **50,** 913–925 (2001).

52. Paradis, E., Claude, J. & Strimmer, K. APE: Analyses of Phylogenetics and Evolution in R language. *Bioinformatics* **20,** 289–290 (2004).

53. Yu, G., Smith, D. K., Zhu, H., Guan, Y. & Tsan-Yuk Lam, T. ggtree: an r package for visualization and annotation of phylogenetic trees with their covariates and other associated data. *Methods Ecol. Evol* **8.** 28–36 (2016).

54. Zachos, J., Pagani, M., Sloan, L., Thomas, E. & Billups, K. Trends, rhythms, and aberrations in global climate 65 Ma to present. *Science* **292**, 686–693 (2001).

55. Ksepka, D. T., Fordyce, R. E., Ando, T. & Jones, C. M. New fossil penguins (Aves, Sphenisciformes) from the Oligocene of New Zealand reveal the skeletal plan of stem penguins. *J. Vert. Paleo.* **32.** 235–254 (2012).

56. Astrin, J. J. et al. Towards a DNA barcode reference database for spiders and harvestmen of Germany. *Plos One* **11,** e0162624 (2016).

57. Matzke, N. J. BioGeoBEARS: BioGeography with Bayesian (and likelihood) evolutionary analysis in R Scripts. R package, version 0.2, 2013, 1. (2013).

58. Seton, M. et al. Global continental and ocean basin reconstructions since 200 Ma. *Earth-Sci. Rev* **113,** 212–270 (2012).

59. Livermore, R., Hillenbrand, C.-D., Meredith, M. & Eagles, G. Drake passage and Cenozoic climate: an open and shut case? *Geochem. Geophy. Geosy* **8** (2007).

60. Gómez-Rubio, V. ggplot2-elegant graphics for data analysis. *J. Stat. Softw* **77,** 1–3 (2017).

61. McIlroy, D. mapproj: Map Projections. R package version 1.1-8.3. http://CRAN.R-project.org/package=mapproj. (2011).

62. Morrison, K. W. & Sagar, P. M. First record of interbreeding between a Snares crested (*Eudyptes robustus*) and erect-crested penguin (*E. sclateri*). *Notornis* **61**, 109–112 (2014).

63. Grosser, S., Burridge, C. P., Peucker, A. J. & Waters, J. M. Coalescent modelling suggests recent secondary-contact of cryptic penguin species. *Plos One* **10**, e0144966 (2015).

64. Hibbets, E. M., Schumacher, K. I., Scheppler, H. B., Boersma, P. D. & Bouzat, J. L. Genetic evidence of hybridization between Magellanic (*Sphensicus magellanicus*) and Humboldt (*Spheniscus humboldti*) penguins in the wild. *Genetica* **148**, 215–228 (2020).

65. Frugone, M.-J. et al. More than the eye can see: Genomic insights into the drivers of genetic differentiation in Royal/Macaroni penguins across the Southern Ocean. *Mol. Phylogenet. Evol* **139,** 106563 (2019).

66. Frugone, M.-J. et al. Contrasting phylogeographic pattern among *Eudyptes* penguins around the Southern Ocean. *Sci. Rep* **8,** 17481 (2018).

67. Mays, H. L. et al. Phylogeography, population structure, and species delimitation in rockhopper penguins (*Eudyptes chrysocome* and *Eudyptes moseleyi*). *J. Hered* **110,** 801–817 (2019).

68. Frugone, M.-J. et al. Taxonomy based on limited genomic markers may underestimate species diversity of rockhopper penguins and threaten their conservation. *Divers. Distrib.* (2021).

69. Banks, J., Van Buren, A., Cherel, Y. & Whitfield, J. B. Genetic evidence for three species of rockhopper penguins, *Eudyptes chrycosome*. *Polar Biol* **30,** 61–67 (2006).

70. Jouventin, P., Cuthbert, R. J. & Ottvall, R. Genetic isolation and divergence in sexual traits: evidence for the northern rockhopper penguin *Eudyptes moseleyi* being a sibling species. *Mol. Ecol* **15,** 3413–3423 (2014).

71. Grosser, S. et al. Invader or resident? Ancient-DNA reveals rapid species turnover in New Zealand little penguins. *P. Roy. Soc. B-Biol. Sci* **283,** 20152879 (2016).

72. Grosser, S., Scofield, R. P. & Waters, J. M. Multivariate skeletal analyses support a taxonomic distinction between New Zealand and Australian *Eudyptula* penguins (Sphenisciformes: Spheniscidae). *Emu* **117,** 276–283 (2017).

73. Sayyari, E., Whitfield, J. B. & Mirarab, S. DiscoVista: Interpretable visualizations

of gene tree discordance. *Mol. Phylogenet. Evol* **122,** 110–115 (2018).

74. Bouckaert, R. R. DensiTree: making sense of sets of phylogenetic trees. *Bioinformatics* **26,** 1372–1373 (2010).

75. Edelman, N. B. et al. Genomic architecture and introgression shape a butterfly radiation. *Science* **366**, 594–599 (2019).

76. Zhang, D. et al. Most genomic loci misrepresent the phylogeny of an avian radiation because of ancient gene flow. *Syst. Biol* syab024 (2021).

77. Pulido-Santacruz, P., Aleixo, A. & Weir, J. T. Genomic data reveal a protracted window of introgression during the diversification of a neotropical woodcreeper radiation. *Evolution.* **74,** 842–858 (2019).

78. Pease, J. B. & Hahn, M. W. Detection and polarization of introgression in a five-taxon phylogeny. *Syst. Biol* **64,** 651–662 (2015).

79. Cahill, J. A., Soares, A. E. R., Green, R. E. & Shapiro, B. Inferring species divergence times using pairwise sequential Markovian coalescent modelling and low-coverage genomic data. *Philos. T. Roy. Soc. B* **371**, 20150138 (2016).

80. Kendig, K. I. et al. Sentieon DNASeq variant calling workflow demonstrates strong computational performance and accuracy. *Front. Genet* **10,** 736 (2019).

81. Ropert-Coudert, Y. et al. Happy feet in a hostile world? The future of penguins depends on proactive management of current and expected threats. *Front. Mar. Sci* **6,** 248 (2019).

82. Trathan, P. N. et al. Pollution, habitat loss, fishing, and climate change as critical threats to penguins. *Cons. Biol* **29,** 31–41 (2014).

83. Boersma, P. D. et al. Applying science to pressing conservation needs for penguins. *Conserv. Biol* **34,** 103–112 (2019).

84. Trucchi, E. et al. King penguin demography since the last glaciation inferred from genome-wide data. *P. Roy. Soc. B-Biol. Sci* **281,** 20140528 (2014).

85. Péron, C., Weimerskirch, H. & Bost, C. A. Predicting king penguins (*Aptenodytes patagonicus*) foraging distribution over the next century. *P. R. Soc. B-Biol. Sci* **279,** 2515–2523 (2012).

86. Fretwell, P. T. & Trathan, P. N. Emperors on thin ice: Three years of breeding failure at Halley Bay. *Antarct. Sci* **31,** 133–138 (2019).

87. Cristofari, R. et al. Full circumpolar migration ensures evolutionary unity in the Emperor penguin. *Nat. Commun* **7,** 11842 (2016).

88. Jenouvrier, S. et al. Projected continent-wide declines of the emperor penguin under

climate change. *Nature Clim Change* **4,** 715–718 (2014).

89. Boersma, P. D. Penguins as marine sentinels. *BioScience* **58**, 597–607 (2008).

90. Younger, J., Emmerson, L., Southwell, C., Lelliott, P. & Miller, K. Proliferation of East Antarctic Adélie penguins in response to historical deglaciation. *BMC Ecol. Evol* **15,** 236 (2015).

91. Younger, J., Van den Hoff, J., Wienecke, B., Hindell, M. & Miller, K. J. Contrasting responses to a climate regime change by sympatric, ice-dependent predators. *BMC Evol Biol* **16,** 61 (2016).

92. Lynch, H. J., Naveen, R., Trathan, P. N. & Fagan, W. F. Spatially integrated assessment reveals widespread changes in penguin populations on the Antarctic Peninsula. *Ecology* **93** (2012).

93. Bost, C. A. et al. Large-scale climatic anomalies affect marine predator foraging behaviour and demography. *Nat. Commun* **6,** 8220 (2015).

94. Croxall, J. P. et al. Seabird conservation status, threats and priority actions: A global assessment. *Bird. Conserv. Int* **22,** 1–34 (2012).

95. Li, H. & Durbin, R. Inference of human population history from individual whole-genome sequences. *Nature* **475,** 493–496 (2011).

96. McKenna, A. et al. The genome analysis toolkit: a MapReduce framework for analyzing next-generation DNA sequencing data. *Genome Res* **20,** 1297–1303 (2010).

97. Palkopoulou, E. et al. A comprehensive genomic history of extinct and living elephants. *P. Natl. Acad. Sci. U.S.A* **115,** E2566–E2574 (2018).

98. Chen, Y. et al. SOAPnuke: a MapReduce acceleration-supported software for integrated quality control and preprocessing of high-throughput sequencing data. *Gigascience* **7,** p.gix120 (2018).

99. Cristofari, R. et al. Climate-driven range shifts of the king penguin in a fragmented ecosystem. *Nat. Clim. Change* **8,** 245 (2018).

100. Cole, T. L. et al. Receding ice drove parallel expansions in Southern Ocean penguins. *P. Natl. Acad. Sci. U.S.A* **116,** 26690–26696 (2019b).

101. De Bruyn, M. et al. Rapid response of a marine mammal species to Holocene climate and habitat change. *Plos Genet* **5,** e1000554 (2009).

102. Fraser, C. I., Nikula, R., Spencer, H. G. & Waters, J. M. Kelp genes reveal effects of subantarctic sea ice during the Last Glacial Maximum. *P. Natl. Acad. Sci. U.S.A* **106,** 3249–3253 (2009).

103. Gillman, L. N. & Wright, S. D. Species richness and evolutionary speed: the

influence of temperature, water and area. *J. Biogeogr* **41,** 39–51 (2014).

104. Quillfeldt, P. Body mass is less important than bird order in determining the molecular rate for bird mitochondrial DNA. *Mol. Ecol* **26,** 2426–2429 (2017).

105. Estandía, A. et al. Substitution rate variation in a robust Procellariiform seabird phylogeny is not solely explained by body mass, flight efficiency, population size or life history traits. bioRxiv (2021).

106. Botero-Castro, F., Figuet, E., Tilak, M.-K., Nabholz, B. & Galtier, N. Avian genomes revisited: Hidden genes uncovered and the rates versus traits paradox in birds. *Mol. Biol. Evol* **34,** 3123–3131 (2017).

107. Rensch, B. *Evolution above the species level* (Methven: London, 1959).

108. Castiglione, S. et al. A new method for testing evolutionary rate variation and shifts in phenotypic evolution. *Methods Ecol. Evol* **9,** 974–983 (2017).

109. Jarvis, E. D. et al. Whole-genome analyses resolve early branches in the tree of life of modern birds. *Science* **346,** 1320–1331 (2014).

110. Tung Ho, L. & Ané, C. A linear-time algorithm for Gaussian and non-Gaussian trait evolution models. *Syst. Biol* **63,** 397–408 (2014).

111. Clavel, J. & Morlon, H. Accelerated body size evolution during cold climatic periods in the Cenozoic. *P. Natl. Acad. Sci. U.S.A* **114,** 4183–4188 (2018).

112. Benson, G. Tandem repeats finder: a program to analyze DNA sequences. *Nucleic Acids Res* **27,** 573–580 (1999).

113. Flynn, J. M. et al. RepeatModeler2 for automated genomic discovery of transposable element families. *P. Natl. Acad. Sci. U.S.A* **117,** 9451–9457 (2020).

114. Davis, L. S. & Renner, M. *Penguins* (London: T & A D Poyser, 2003).

115. Stonehouse, B. The general biology and thermal balances of penguins. *Adv. Ecol. Res* **4**, 131–196 (1967).

116. Zhao, H., Li, J. & Zhang, J. Molecular evidence for the loss of three basic tastes in penguins. *Curr. Biol* **25,** R141–R142 (2015).

117. Yang, Z. PAML: a program package for phylogenetic analysis by maximum likelihood. *Bioinformatics* **13,** 555–556 (1997).

118. Yang, Z. PAML 4: a program package for phylogenetic analysis by maximum likelihood. *Mol. Biol. Evol* **24,** 1586–1591 (2007).

119. Löytynoja, A. *Phylogeny-aware alignment with PRANK*. In *Multiple sequence alignment methods* (Totowa, NJ: Humana Press, 2014).

120. Sela, I., Ashkenazy, H., Katoh, K. & Pupko, T. GUIDANCE2: accurate detection of unreliable alignment regions accounting for the uncertainty of multiple parameters. *Nucleic Acids Res* **43,** W7–W14 (2015).

121. Choi, Y., Sims, G. E., Murphy, S., Miller, J. R. & Chan, A. P. Predicting the functional effect of amino acid substitutions and indels. *PLoS ONE* **7,** e46688 (2012).

122. Kramer, R. & Molokanova, E. Modulation of cyclic-nucleotide-gated channels and regulation of vertebrate phototransduction. *J. Exp. Biol* **204,** 2921–31 (2001).

123. Liu, C. et al. A role for the mesenchymal T-box gene Brachyury in AER formation during limb development. *Development*. **130,** 1327–1337 (2003).

124. Adams, K. L., Rousso, D. L., Umbach, J. A. & Novitch, B. G. *Foxp1*-mediated programming of limb-innervating motor neurons from mouse and human embryonic stem cells. *Nat. Commun* **6,** 6778 (2015).

125. Le Goff, C. et al. Heterozygous mutations in *MAP3K7*, encoding TGF-β-activated kinase 1, cause cardiospondylocarpofacial syndrome. *Am. J. Hum. Genet* **99,** 407–413 (2016).

126. Yang, N. & Meng, Q.-J. Circadian clocks in articular cartilage and bone: A compass in the sea of matrices. *J. Biol. Rhythms* **31,** 415–427 (2016).

127. Li, H. et al. *FOXP1* controls mesenchymal stem cell commitment and senescence during skeletal aging. *J. Clin. Invest* **127,** 1241–1253 (2017).

128. Singh, P. N. P. et al. *NFIA* and *GATA3* are crucial regulators of embryonic articular cartilage differentiation. *Development* **145,** dev156554 (2018).

129. Xu, H. & Liu, F. Downregulation of *FOXP1* correlates with tendon stem/progenitor cells aging. *Biochem. Biophys. Res. Commun* **504,** 96–102 (2018).

130. Kitami, M. et al. *IFT20* is required for the maintenance of cartilaginous matrix in condylar cartilage. *Biochem. Biophys. Res. Commun* **509,** 222–226 (2019).

131. Finnson, K. W., Chi, Y., Bou-Gharios, G., Leask, A. & Philip, A. TGF-b signaling in cartilage homeostasis and osteoarthritis. *Front Biosci*. (Schol. Ed.) **4,** 251–268 (2012).

132. Docheva, D., Hunziker, E. B., Fässler, R. & Brandau, O. Tenomodulin is necessary for tenocyte proliferation and tendon maturation. *Mol. Cell. Biol* **25,** 699–705 (2005).

133. Kishimoto, Y. et al. Wnt/β-catenin signaling suppresses expressions of *Scx*, *Mkx*, and *Tnmd* in tendon-derived cells. *Plos One* **12,** e0182051 (2017).

134. Li, L., Stoeckert, C. J. & Roos, D. S. OrthoMCL: identification of ortholog groups for eukaryotic genomes. *Genome Res* **13,** 2178–2189 (2003).

135. Guindon, S. et al. New algorithms and methods to estimate maximum-likelihood phylogenies: assessing the performance of PhyML 3.0. *Syst. Biol* **59,** 307–321 (2010).

136. Fish, F. E. Secondary evolution of aquatic propulsion in higher vertebrates: validation and prospect. *Integr. Comp. Biol* **56,** 1285–1297 (2016).

137. Raccurt, M. et al. Growing in Antarctica, a challenge for white adipose tissue development in Adelie penguin chicks (*Pygoscelis adeliae*). *Am. J. Physiol* **295,** R1671–R1679 (2008).

138. Deepa, S. S. & Dong, L. Q. APPL1: role in adiponectin signaling and beyond. *Am. J. Physiol. Endocrinol. Metab* **296,** E22–E36 (2009).

139. Sukumar, P. et al. Constitutively active *TRPC* channels of adipocytes confer a mechanism for sensing dietary fatty acids and regulating adiponectin. *Circ. Res* **111,** 191–200 (2012).

140. Tamburrini, M., Romano, M., Giardina, B. & di Prisco, G. The myoglobin of Emperor penguin (*Aptenodytes forsteri*): amino acid sequence and functional adaptation to extreme conditions. *Comp. Biochem. Physiol. B. Biochem. Mol. Biol* **122,** 235–40 (1999).

141. Kooyman, G. L. & Ponganis, P. J. The physiological basis of diving to depth: Birds and mammals. *Annu. Rev. Physiol* **60,** 19–32 (1998).

142. Meir, J. U. & Ponganis, P. J. High-affinity hemoglobin and blood oxygen saturation in diving emperor penguins. *J. Exp. Biol* **212,** 3330–8 (2009).

143. Wienecke, B., Robertson, G., Kirkwood, R. & Lawton, K. Extreme dives by free-ranging emperor penguins. *Polar Biol* **30,** 133–142 (2007).

144. Nery, M. F., Arroyo, J. I. & Opazo, J. C. Accelerated evolutionary rate of the myoglobin gene in long-diving whales. *J. Mol. Evol* **76,** 380–387 (2013).

145. Jumper, J. et al. Highly accurate protein structure prediction with AlphaFold. *Nature* (2021).

146. Guex, N. & Peitsch, M. C. SWISS-MODEL and the Swiss-Pdb Viewer: an environment for comparative protein modeling. *Electrophoresis* **18,** 2714–2723 (1997).

147. Crooks, G. E., Hon, G., Chandonia, J. M. & Brenner, S. E. WebLogo: A sequence logo generator. *Genome Res* **14,** 1188–1190 (2004).

148. Naito, Y. et al. Transferrin Receptor 1 in Chronic Hypoxia-Induced Pulmonary Vascular Remodeling. *Am. J. Hypertens* **29,** 713–718 (2016).

149. Rolfs, A., Kvietikova, I., Gassmann, M. & Wenger, R. H. Oxygen-regulated transferrin expression is mediated by hypoxia-inducible factor-1. *J. Biol. Chem* **8,**

0055–62 (1997).

150. Friedrich, J. & Wiener, P. Selection signatures for high-altitude adaptation in ruminants. *Anim. Genet* **51,** 157–65 (2020).

151. Dasmeh, P., Serohijos, A. W. R., Kepp, K. P. & Shakhnovich, E. I. Positively selected sites in cetacean myoglobins contribute to protein stability. *PLoS Comput. Biol* **9,** e1002929 (2013).

152. Freichel, M., Philipp, S., Cavalié, A. & Flockerzi, V. *TRPC4* and *TRPC4*-deficient mice. *Novartis Found Symp* **258,** 189–199 (2014).

153. Bowmaker, J. K. & Martin, G. R. Visual pigments and oil droplets in the penguin, *Spheniscus humboldti*. *J. Comp. Physiol* **156,** 71–77 (1985).

154. Gondo, M. & Ando, H. Comparative histophysiological study of oil droplets in the avian retina. *Jpn. J. Ornithol* **44,** 81–91 (1995).

155. Martin, G. R. Extreme pupillary response Eye structure and foraging in King Penguins *Aptenodytes patagonicus*. *Ibis*. **141,** 444–450 (1999).

156. Suburo, A. M. & Scolaro, J. A. Environmental adaptations in the retina of the magellanic penguin: photoreceptors and outer plexiform layer. *Waterbirds* **22,** 111–119 (1999).

157. Hölter, P. et al. The retinal clock drives the expression of *Kcnv2*, a channel essential for visual function and cone survival. *Invest. Ophthalmol. Vis. Sci* **53,** 6947–6954 (2012).

158. Vincent, A. et al. Biallelic mutations in *GNB3* cause a unique form of autosomal-recessive congenital stationary night blindness. *Am. J. Hum. Genet* **98,** 1011–1019 (2016).

159. Thomas, D. B., McGoverin, C. M., McGraw, K. J., James, H. F. & Madden, O. Vibrational spectroscopic analyses of unique yellow feather pigments (spheniscins) in penguins. *J. Roy. Soc. Interface* **10** (2013).

160. Murakami, T. et al. Signalling mediated by the endoplasmic reticulum stress transducer OASIS is involved in bone formation. *Nat. Cell Biol* **11,** 1205–1211 (2009).

161. Schoor, M., Schuster-Gossler, K., Roopenian, D. & Gossler, A. Skeletal dysplasias, growth retardation, reduced postnatal survival, and impaired fertility in mice lacking the SNF2/SWI2 family member ETL1. *Mech. Dev* **85,** 73–83 (1999).

162. Doolittle, R. F. Fibrinogen and fibrin. *Ann. Rev. Biochem* **53,** 195–229 (1984).

163. Yang, H. et al. *TMEM16F* forms a Ca2+-activated cation channel required for lipid scrambling in platelets during blood coagulation, *Cell* **151,** 111–122 (2012).

164. Levy, H. et al. Evidence of pathogen-induced immunogenetic selection across the large geographic range of a wild seabird. *Mol. Biol. Evol* **37,** 1708–1726 (2020).

165. Velová, H., Gutowska-Ding, M. W., Burt, D. W. & Vinkler, M. Toll-like receptor evolution in birds: gene duplication, pseudogenization, and diversifying selection. *Mol. Biol. Evol* **35,** 2170–2184 (2018).

166. Yoon, S. I. et al Structural basis of *TLR5*-flagellin recognition and signaling. *Science* **335,** 859–864 (2012).

167. Zhang, B., Liu, X., Chen, W. & Chen, L. *IFIT5* potentiates anti-viral response through enhancing innate immune signaling pathways. *Acta Biochimic. Biophys. Sin* **45,** 867–874 (2013).

168. Katibah, G. E. et al. tRNA binding, structure, and localization of the human interferon-induced protein IFIT5. *Mol. Cell* **49,** 743–750 (2013).

169. Katibah, G. E. et al. Broad and adaptable RNA structure recognition by the human interferon-induced tetratricopeptide repeat protein IFIT5. *P. Natl. Acad. Sci. U.S.A* **111,** 12025–12030 (2014).

170. Zhang, J. et al. *CD81* is required for hepatitis C virus glycoprotein-mediated viral infection. *J. Virol* **78,** 1448–55 (2004).

171. Forni, D. et al. Evolutionary analysis provides insight into the origin and adaptation of HCV. *Front. Microbiol* **9,** 854 (2018).

172. Cassat, J. E. & Skaar, E. P. Iron in infection and immunity. *Cell Host Microbe* **13,** 509–519 (2013).

173. Saunderson, S. C. et al. Phylogenomic characterization of a novel corynebacterium species associated with fatal diphtheritic stomatitis in endangered yellow-eyed penguins. *mSystems* **6,** e0032021 (2021).

174. Mattern, T. et al. Straight line foraging in yellow-eyed penguins: new insights into cascading fisheries effects and orientation capabilities of marine predators. *PLoS ONE* **8,** e84381 (2013).

175. Barber, M. F. & Elde, N. C. Escape from bacterial iron piracy through rapid evolution of transferrin. *Science* **346,** 1362–1366 (2014).

176. Yang, L. & Gui, J. F. Positive selection on multiple antique allelic lineages of transferrin in the polyploid *Carassius auratus*. *Mol. Biol. Evol* **21,** 1264–1277 (2004).

177. Ford, M. J. Molecular evolution of transferrin: Evidence for positive selection in salmonids. *Mol. Biol. Evol* **18,** 639–647 (2001).

178. Edgar, R. C. MUSCLE: Multiple sequence alignment with high accuracy and high throughput. *Nucleic Acids Res* **32,** 1792–1797 (2004).

179. Kearse, M. et al. Geneious Basic: An integrated and extendable desktop software platform for the organization and analysis of sequence data. *Bioinformatics* **28,** 1647–1649 (2012).

180. Borges, R. et al. Gene loss, adaptive evolution and the co-evolution of plumage coloration genes with opsins in birds. *BMC Genomics* **16,** 751 (2015).

181. Feuda, R., Hamilton, S. C., McInerney, J. O. & Pisani, D. Metazoan opsin evolution reveals a simple route to animal vision. *P. Natl. Acad. Sci. U.S.A* **109,** 18868–18872 (2012).

182. Meredith, R. W., Gatesy, J., Emerling, C. A., York, V. M. & Springer, M. S. Rod monochromacy and the coevolution of cetacean retinal opsins. *Plos Genet* **9,** e1003432 (2013).

183. Yokoyama, S., Yang, H. & Starmer, W. T. Molecular basis of spectral tuning in the red- and green-sensitive (M/LWS) pigments in vertebrates. *Genetics* **179,** 2037–2043 (2008).

184. Toomey, M. B. et al. A complex carotenoid palette tunes avian colour vision. *J. Roy. Soc. Interface* **12** (2015).

185. Emerling, C. A. Independent pseudogenization of *CYP2J19* in penguins, owls and kiwis implicates gene in red carotenoid synthesis. *Mol. Phylogenet. Evol* **118,** 47–53 (2018).

186. Yarmolinsky, D. A., Zuker, C. S. & Ryba, N. J. P. Common sense about taste: from mammals to insects. *Cell* **139,** 234–244 (2009).

187. Janiak, M. C., Chaney, M. E. & Tosi, A. J. Evolution of acidic mammalian chitinase genes (*CHIA*) is related to body mass and insectivory in primates. *Mol. Biol. Evol* **35,** 607–622 (2018).

188. Chen, Y. H. & Zhao, H. Evolution of digestive enzymes and dietary diversification in birds. *PeerJ* **25,** e6840 (2019).

189. Jackson, S., Place, A. R. & Seiderer, L. J. Chitin digestion and assimilation by seabirds. *The Auk* **109,** 758–770 (1992).
