# Supplementary material for: Genomic insights into the secondary aquatic transition of penguins
Source: Nat Commun. 2022 Jul 19;13:3912. doi: 10.1038/s41467-022-31508-9 (PMC9296559; doi:10.1038/s41467-022-31508-9)
Supplement: Supplementary file 2 — Description of Additional Supplementary Files [file 41467_2022_31508_MOESM2_ESM.pdf]

Description of Additional Supplementary Files

Supplementary Movie 1 Penguin vision experiment. A *Pygoscelis papua* penguin can be observed following the projection of a UV torch onto the snow at SEALIFE Kelly Tarlton's Aquarium, Auckland, a projection which was not visible to the human observers. The torch's output was maximal at approximately 365 nm, tapering to no output by 390 nm.

Supplementary Data 1 The basic information for assembly, annotation, morphological characters, fossil calibrations, foraging distances, genetic distances and phylogenetic analyses. Biogeobears models for the evolution of penguins geographic ranges reconstruction were compared with Likelihood Ratio Test and Akaike information criterion, and the P values of the null hypothesis testing were shown in the table.

Supplementary Data 2 Introgression and ILS quantification results, penguin evolutionary rate and heterozygosity rate. One sided T-tests or Wilcoxon-tests were performed for heterozygosity rate comparison for penguins (n=24) and other avian orders (n>3) and P-values were shown in the table.

Supplementary Data 3 Evolutionary rate comparison and repeat elements.

Supplementary Data 4 Genes involved in penguin specific adaptations from all methods. Genes were identified under branch, branch-site or site models using PAML. P values were calculated using the chi-square test and P-values were adjusted by Benjamini and Hochberg method.

Supplementary Data 5 Phylogenomic dataset. S5.1 Morphological dataset. The full matrix contains 72 fossil and extant penguin taxa, two outgroup taxa, and 281 morphological characters. We expanded the morphological dataset of [24], including a morphological tree and the morphological matrix by incorporating several additional fossil penguin species, including *Crossvalia waiparensis*, *Crossvallia unienwillia*, *Kupoupou stilwelli*, and *Kaiika maxwelli,* and seven additional morphological characters. S5.2 Phylogenomic trees inferred from all methods. S5.3 Datasets for dating analyses. The configuration files for the node-dating and the Bayesian total-evidence dating analyses and the dating results from the dating analyses. S5.4 Randomly selected trees for densitree plot.

Supplementary Data 6 Introgression analyses dataset. S6.1 Introgression analyses Commands. S6.2 Results from hPSMC analysis.

Supplementary Data 7 Genes involved in penguin specific adaptations. Protein structure files of myoglobin proteins and hemoglobin proteins. S7.1 Comparison of penguin-specific substitutions between penguins and other birds. S7.2 Protein structure files of myoglobin proteins and hemoglobin proteins. The penguin-specific amino acid substitutions may be responsible for the stabilization in penguin myoglobin and hemoglobin proteins.

Supplementary Software. Codes used in this stud
