# Supplementary material for: Genomic insights into the secondary aquatic transition of penguins
Source: Nat Commun. 2022 Jul 19;13:3912. doi: 10.1038/s41467-022-31508-9 (PMC9296559; doi:10.1038/s41467-022-31508-9)
Supplement: Supplementary file 12 — Reporting summary [file 41467_2022_31508_MOESM12_ESM.pdf]

nature portfolio

Corresponding author(s): Theresa L. Cole

Last updated by author(s): May 18, 2022

Reporting Summary

Nature Portfolio wishes to improve the reproducibility of the work that we publish. This form provides structure for consistency and transparency in reporting. For further information on Nature Portfolio policies, see our Editorial Policies and the Editorial Policy Checklist.

Statistics

For all statistical analyses, confirm that the following items are present in the figure legend, table legend, main text, or Methods section.

n/a	Confirmed	
☐	☒	The exact sample size (*n*) for each experimental group/condition, given as a discrete number and unit of measurement
☐	☒	A statement on whether measurements were taken from distinct samples or whether the same sample was measured repeatedly
☐	☒	The statistical test(s) used AND whether they are one- or two-sided<br>*Only common tests should be described solely by name; describe more complex techniques in the Methods section.*
☒	☐	A description of all covariates tested
☐	☒	A description of any assumptions or corrections, such as tests of normality and adjustment for multiple comparisons
☐	☒	A full description of the statistical parameters including central tendency (e.g. means) or other basic estimates (e.g. regression coefficient) AND variation (e.g. standard deviation) or associated estimates of uncertainty (e.g. confidence intervals)
☐	☒	For null hypothesis testing, the test statistic (e.g. *F*, *t*, *r*) with confidence intervals, effect sizes, degrees of freedom and *P* value noted<br>*Give P values as exact values whenever suitable.*
☐	☒	For Bayesian analysis, information on the choice of priors and Markov chain Monte Carlo settings
☒	☐	For hierarchical and complex designs, identification of the appropriate level for tests and full reporting of outcomes
☒	☐	Estimates of effect sizes (e.g. Cohen's *d*, Pearson's *r*), indicating how they were calculated

Our web collection on statistics for biologists contains articles on many of the points above.

Software and code

Policy information about availability of computer code

Data collection	No software was used for data collection
Data analysis	SOAPnuke v1.5.6 https://github.com/BGI-flexlab/SOAPnuke<br>Supernova v2.0.0 https://github.com/10XGenomics/supernova<br>PALEOMIX pipeline v1.2.12 https://paleomix.readthedocs.io/en/v1.2.12/index.html<br>AdapterRemoval2 v2.2.0 https://github.com/MikkelSchubert/adapterremoval<br>BWA v0.7.17 https://github.com/lh3/bwa<br>MapDamage v2.0.9 https://ginolhac.github.io/mapDamage/<br>SAMtools v1.4.1, v1.8 https://github.com/samtools/samtools<br>BCFtools v1.8 http://www.htslib.org/doc/bcftools.html<br>GATK v4.0.5.2 https://github.com/broadinstitute/gatk<br>Picard v1.115 http://broadinstitute.github.io/picard/<br>BLAST v2.2.26 https://blast.ncbi.nlm.nih.gov/<br>Genewise v2.4.1 https://www.ebi.ac.uk/Tools/psa/genewise/<br>PSMC v0.6.4-r49 https://github.com/lh3/psmc<br>Augustus v3.3.1 https://github.com/Gaius-Augustus/Augustus<br>BUSCO v3.0.2 https://gitlab.com/ezlab/busco<br>Cactus v49e80082 https://github.com/ComparativeGenomicsToolkit/cactus<br>Tandem Repeats Finder v4.07b https://github.com/Benson-Genomics-Lab/TRF<br>Repeat-Masker v4.0.7 https://www.repeatmasker.org/<br>RepeatModeler v1-0-8 https://www.repeatmasker.org/RepeatModeler/<br>MP-EST v2.0 http://faculty.franklin.uga.edu/lliu/mp-est

ASTRAL-III https://github.com/smirarab/ASTRAL
ExaML v3.0.21 https://cme.h-its.org/exelixis/web/software/examl/index.html
RAxML v8.2.12 https://cme.h-its.org/exelixis/web/software/raxml/
IQtree v1.6.12 http://www.iqtree.org/
BEAST2 https://www.beast2.org/
Tracer v1.7.1 http://beast.community/tracer
PRANK v170427 http://wasabiapp.org/software/prank/
GUIDANCE v2.02 http://guidance.tau.ac.il/source.php
PAML v4.9i http://abacus.gene.ucl.ac.uk/software/paml.html
PROVEAN v.1.1.5 http://provean.jcvi.org/index.php
DiStats v1.0 https://github.com/mptrsen/distats
DiscoVista v1.0 https://github.com/esayyari/DiscoVista
QuIBL v1.0 https://github.com/miriammiyagi/QuIBL
DFOIL v1.0 https://github.com/jbpease/dfoil
hPSMC v1.0 https://github.com/jacahill/hPSMC
Sentieon v0.2.0 https://github.com/Sentieon
R v3.6.3 https://www.r-project.org/
BioGeoBEARS v1.1.1 http://phylo.wikidot.com/biogeobears
maproj v1.2 http://CRAN.R-project.org/package=mapproj
ggtree v2.4.2 https://github.com/YuLab-SMU/ggtree
ape v5.5 http://ape-package.ird.fr/
RRphylo v2.5.8 https://github.com/pasraia/RRphylo
phylolm v2.6.2 https://github.com/lamho86/phylolm
ggplot2 v3.3.5 https://ggplot2.tidyverse.org/
AlphaFold2 https://github.com/deepmind/alphafold
SWISS-MODEL v2021.5 https://swissmodel.expasy.org/
OrthoMCL v1.4 https://orthomcl.org/orthomcl/app
PhyML v3.0 http://www.atgc-montpellier.fr/phyml
Geneious v2019.2.3 https://www.geneious.com/
MUSCLE v3.5 https://www.ebi.ac.uk/Tools/msa/muscle/
The custom scripts used in this study have been deposited on Figshare [DOI: 10.6084/m9.figshare.c.5535243]

For manuscripts utilizing custom algorithms or software that are central to the research but not yet described in published literature, software must be made available to editors and reviewers. We strongly encourage code deposition in a community repository (e.g. GitHub). See the Nature Portfolio guidelines for submitting code & software for further information.

Data

Policy information about availability of data

All manuscripts must include a data availability statement. This statement should provide the following information, where applicable:

- Accession codes, unique identifiers, or web links for publicly available datasets
- A description of any restrictions on data availability
- For clinical datasets or third party data, please ensure that the statement adheres to our policy

The sequencing data and genome assemblies generated in this study have been deposited in the NCBI database under BioProject PRJNA722815 [https://www.ncbi.nlm.nih.gov/bioproject/PRJNA722815] and PRJNA556735 [https://www.ncbi.nlm.nih.gov/bioproject/?term=PRJNA556735], as well as the CNSA of the CNGBdb database under the accession number CNP0000605 [https://db.cngb.org/search/project/CNP0000605/].
Supplementary datasets have been deposited on Figshare [DOI: 10.6084/m9.figshare.c.5535243].
The following datasets were also used in this study: CNSA accession number CNP0000505 [https://db.cngb.org/search/project/CNP0000505/], and NCBI Genbank accession number NP_990272 [https://www.ncbi.nlm.nih.gov/protein/NP_990272.2/], NP_001071646 [https://www.ncbi.nlm.nih.gov/protein/NP_001071646], NP_001071647 [https://www.ncbi.nlm.nih.gov/protein/NP_001071647].

Field-specific reporting

Please select the one below that is the best fit for your research. If you are not sure, read the appropriate sections before making your selection.

☐ Life sciences ☐ Behavioural & social sciences ☒ Ecological, evolutionary & environmental sciences

For a reference copy of the document with all sections, see nature.com/documents/nr-reporting-summary-flat.pdf

Ecological, evolutionary & environmental sciences study design

All studies must disclose on these points even when the disclosure is negative.

Study description	Within the framework of a genome-scale, fossil-inclusive phylogeny, we identify key geological events that shaped penguin diversification and genomic signatures consistent with widespread refugia/recolonization during major climate oscillations. We identify a suite of genes underpinning adaptations related to thermoregulation, oxygenation, diving, vision, diet, immunity and body size. We also document that penguins and their sister group (Procellariiformes) have the lowest evolutionary rates yet detected in birds. Together, these findings help resolve the enigma of how penguins have transitioned to the marine environment, successfully

colonizing some of the most extreme environments on Earth.

Research sample	We used genomes from all extant and recently extinct penguin lineages (27 taxa), stratigraphic data from fossil penguins (47 taxa), and morphological and biogeographic data from all species (extant and extinct). 21 of the genomes have previously been published by members of our consortium: the emperor (Aptenodytes forsteri) and Adélie (Pygoscelis adeliae) penguin genomes have been available since 2011, and 19 genomes encompassing all extant species and subspecies were published as an early-release dataset for this project in 2019, spanning: king (Aptenodytes patagonicus), chinstrap (Pygoscelis antarctica), gentoo (P. papua West Antarctic Peninsula “WAP” lineage), macaroni (Eudyptes chrysolophus chrysolophus), royal (E. c. schlegeli), northern rockhopper (Eudyptes moseleyi), eastern rockhopper (Eudyptes filholi), southern rockhopper (Eudyptes chrysocome), Fiordland (Eudyptes pachyrhynchus), Snares (Eudyptes robustus), erect-crested (Eudyptes sclateri), yellow-eyed (Megadyptes antipodes antipodes), Australian fairy (Eudyptula novaehollandiae), New Zealand little (Eudyptula minor), New Zealand little Banks Peninsula/white-flippered lineage (“BAN”), African (Spheniscus demersus), Magellanic (Spheniscus magellanicus), Humboldt (Spheniscus humboldti) and Galápagos (Spheniscus mendiculus) penguins. To supplement our dataset, we also sequenced three high-coverage genomes from the remaining major gentoo penguin lineages, comprising the Falkland Islands/Malvinas (“FAL”), Kerguelen Island (“KER”) and South Georgia (“SG”) as well as three partial genomes for all known recently extinct taxa, Chatham Islands crested penguin, Chatham Islands Megadyptes penguin, and the Waitaha penguin. To compare our penguin genomes to other bird genomes, we obtained the 361 bird genomes recently released as part of the B10K project (https://b10k.genomics.cn). These genomes represent 36 bird orders and 218 bird families.
Sampling strategy	Our manuscript is focused on the species-level evolutionary history of penguins. For this reason, we analysed a single genome per species/major lineage, rather than multiple individuals/populations per species/major lineage. The questions addressed and analyses undertaken in this study requires just a single genome per species/major lineage, and multiple samples per species/major lineage is beyond the scope of this study and would address different questions than those asked in this study.
Data collection	The data used in our study includes genome data, which was extracted directly from blood/tissue samples of penguins collected in the wild, zoo or museums by Theresa L. Cole, Alan J. D. Tennyson, Charles-André Bost, Yves Cherel, Thomas Mattern, Tom Hart, Lara D. Shepherd, Richard A. Phillips, Petra Quillfeldt, Juan F. Masello, Juan L. Bouzat, Peter G. Ryan, David R. Thompson, Ursula Ellenberg, Peter Dann, Gary Miller, P. Dee Boersma or was obtained from early-release data by our consortium (see Pan H, Cole TL et al.(2019). High-coverage genomes to elucidate the evolution of penguins. GigaScience 8: 9, giz117). The genome data was generated and analysed by Chengran Zhou, Miaoquan Fang, Hailin Pan, Daniel T. Ksepka, Steven R. Fiddaman, Christopher A. Emerling, Daniel B. Thomas, Xupeng Bi, Qi Fang, Martin R. Ellegaard, Shaohong Feng, Tracy A. Heath, Jamie R. Wood, Mikkel-Holger S. Sinding, Lara D. Shepherd and Theresa L. Cole. Additional genomic data includes comparison with other bird genomes from recently released by the B10K project (https://b10k.genomics.cn). These genomes represent 36 bird orders and 218 bird families (see Feng, S. et al. Dense sampling of bird diversity increases power of comparative genomics. Nature 587, 252–257 (2020).<br><br>We also analysed and expanded on the morphological data from Thomas, D. et al. Ancient crested penguin constrains timing of recruitment into seabird hotspot. P. Roy. Soc. B-Biol. Sci 287, 20201497 (2020), by incorporating several additional fossil penguin species (undertaken by Daniel T. Ksepka), including Crossvalia waiparensis, Crossvallia unienwillia, Kupoupou stilwelli, and Kaiika maxwelli, and seven additional morphological characters.<br><br>We extracted average sea surface temperatures (calculated from a maximum of 20 spot locations obtained from near breeding colonies during both summer and winter seasons) for penguin lineages from https://earth.nullschool.net.<br><br>Generation time of each extant penguin lineage was obtained from the IUCN, defined as the mean age of reproduction. For Megadyptes antipodes richdalei we use the same generation time as Megadyptes antipodes antipodes as it is the closest represented lineage.
Timing and spatial scale	The spatial scale of our genomes and associated data encompasses all penguin species/lineages. The samples and relevant data were obtained opportunistically from sites across the entire Southern Ocean.
Data exclusions	NA
Reproducibility	All attempts to repeat the experiment on the data obtained were successful: 500 bootstrap replicates for each locus were used for RAxML analysis, 1000 ultrafast bootstrap replicates was used for each locus for IQTREE analysis. We also verified the reproducibility of gene selection analysis by adding the qualitative comparisons of the identified penguin genes with over 300 other avian species.
Randomization	Our data consisted of genomes from single representative individuals and therefore did not require randomization.
Blinding	No blinding needed, as we were using genome data from single representative individuals and we didn't have any a priori expectation regarding our results.

Did the study involve field work? ☒ Yes ☐ No

Field work, collection and transport

Field conditions	NA
Location	New Island, Falkland Islands/Malvinas<br>Pointe Morne, Kerguelen Island<br>Bird Island, South Georgia<br><br>Exact latitutde/longitude locations not available as these are historical samples.

Access & import/export	New Island, Falkland Islands/Malvinas: Research License No: R15/2017; Date: 2018/2019 season; Pointe Morne, Kerguelen Island: Collection permit and animal ethics permissions provided by the TAAF: Arrêté n° 2012-119 du 29 Octobre 2012 Autorisant la réalisation du programme 394, Oiseaux Plongeurs, Date: 2012-2013; Bird Island, South Georgia (n = 2; Permit: GSGSSI permit, BAS 2001-02; Date: 2005/2006 season.
Disturbance	NA - The samples used in this study were collected as part of a monitoring programme/other research on penguins, and were only integrated in this study several years after they had been collected. All blood samples were collected by trained professionals using sterile equipment, and the live birds were only physically handled for short (>15 minute) periods to avoid disturbance, with care being taken when entering the colony. All animal ethics/permits were adhered to.

Reporting for specific materials, systems and methods

We require information from authors about some types of materials, experimental systems and methods used in many studies. Here, indicate whether each material, system or method listed is relevant to your study. If you are not sure if a list item applies to your research, read the appropriate section before selecting a response.

Materials & experimental systems

n/a	Involved in the study
☒	☐ Antibodies
☒	☐ Eukaryotic cell lines
☐	☒ Palaeontology and archaeology
☐	☒ Animals and other organisms
☒	☐ Human research participants
☒	☐ Clinical data
☒	☐ Dual use research of concern

Methods

n/a	Involved in the study
☒	☐ ChIP-seq
☒	☐ Flow cytometry
☒	☐ MRI-based neuroimaging

Palaeontology and Archaeology

Specimen provenance	We took small subsamples (Chatham Islands Megadyptes penguin: S.29384.35; Waitaha penguin: S.42156.6; Chatham Islands crested penguin: S.29384.37) of bone from specimens in the Museum of New Zealand Te Papa Tongarewa collection. To minimise damage to the collection, the samples chosen were already damaged and were not type specimens. A permit to export the samples permanently was obtained from the New Zealand Ministry for Culture and Heritage. Samples were sent to the GLOBE Institute at the University of Copenhagen for ancient DNA extraction.
Specimen deposition	The specimens have been destroyed following DNA extraction, but the genome data has been uploaded to NCBI database under BioProject PRJNA722815 and PRJNA556735, as well as the CNSA (https://db.cngb.org/cnsa/) of the CNGBdb database under the accession number CNP0000605 and are publically available as of the date of publication.
Dating methods	NA

☐ Tick this box to confirm that the raw and calibrated dates are available in the paper or in Supplementary Information.

Ethics oversight	Museum of New Zealand Te Papa Tongarewa<br>New Zealand Ministry for Culture and Heritage

Note that full information on the approval of the study protocol must also be provided in the manuscript.

Animals and other organisms

Policy information about studies involving animals; ARRIVE guidelines recommended for reporting animal research

Laboratory animals	NA
Wild animals	Gentoo penguin (Pygoscelis papua) blood was obtained from New Island, Falkland Islands/Malvinas (n = 2), Pointe Morne, Kerguelen Island (n = 1) and Bird Island, South Georgia (n = 2). The samples used in this study were collected as part of a monitoring programme/other research on penguins, and were only integrated in this study several years after they had been collected. All blood samples were collected by trained professionals using sterile equipment, and the live birds were only physically handled for short (>15 minute) periods to avoid disturbance (and were released immediately after), with care being taken when entering the colony to not disturb other birds.
Field-collected samples	NA
Ethics oversight	Samples were collected by Falkland Islands/Malvinas Research License No: R15/2017; Date: 2018/2019 season, Kerguelen Island Collection permit and animal ethics permissions provided by the TAAF: Arrêté n° 2012-119 du 29 Octobre 2012 Autorisant la réalisation du programme 394, Oiseaux Plongeurs; Date: 2012-2013 and South Georgia Permit: GSGSSI permit, BAS (British Antarctic Survey) 2001-02; Date: 2005/2006 season.

Note that full information on the approval of the study protocol must also be provided in the manuscript.
